# Clinical efficacy and safety of acupuncture in the treatment for chronic spontaneous urticaria: a systematic review and meta-analysis

**DOI:** 10.3389/fmed.2025.1498795

**Published:** 2025-05-30

**Authors:** Wang Wei, Yuxiang Wu, Shengyan Zhang, Bushuang Li, Zhengda Cheng, Wenjie Zhao

**Affiliations:** ^1^Beijing University of Traditional Chinese Medicine Xiamen Hospital, Xiamen, China; ^2^Fujian University of Traditional Chinese Medicine, Fuzhou, China; ^3^Department of Traditional Chinese Medicine, Qinghai University Medical College, Xining, China; ^4^Jinjiang Traditional Chinese Medicine Hospital, Quanzhou, China

**Keywords:** acupuncture, chronic spontaneous urticaria, urticaria, meta-analysis, systematic review

## Abstract

**Objective:**

This study aims to systematically evaluate the clinical efficacy and safety of acupuncture in treatment for chronic spontaneous urticaria (CSU) and provide evidence to inform clinical decision-making.

**Methods:**

A comprehensive search of eight Chinese and English databases was carried out. The search period spanned from the inception of the database up to 20 August 2024, and the search included randomized controlled trials (RCTs) on acupuncture for CSU, without language restrictions. Two independent researchers screened the resulting studies, evaluated their quality, and cross-checked their results. The extracted data were subjected to meta-analysis using RevMan 5.4 and Stata 15.

**Results:**

A total of 22 RCTs involving 1,867 patients were included. Meta-analysis showed that acupuncture significantly improved the overall response rate, reduced the recurrence rate, decreased the urticaria activity score, and improved the Dermatology Life Quality Index, Hamilton Depression Scale, VAS itching score, and the Chronic Urticaria Quality of Life Questionnaire scores. Acupuncture also resulted in a reduced number and size of wheals, shortened duration of flare-ups, and reduced serum IgE, IFN-*γ*, and IL-4 levels. In addition, it led to significantly reduced traditional Chinese medicine syndrome scores, all with statistical significance. Furthermore, acupuncture did not significantly increase the incidence of adverse events, which indicates good safety. However, moderate to high bias and heterogeneity were observed in the included RCTs. Based on the Grading of Recommendations, Assessment, Development, and Evaluation evidence, this study provides a moderate to low recommendation for acupuncture in the treatment for CSU although the results remain promising.

**Conclusion:**

Acupuncture appears to be an effective and safe treatment for CSU. However, further high-quality RCTs are needed to confirm its clinical efficacy and safety.

## 1 Introduction

Urticaria (1) is a common dermatological condition that is primarily driven by mast cells (MCs) and characterized by wheals of varying sizes and associated itching. If these wheals appear intermittently or daily for more than 6 weeks, the condition is diagnosed as chronic spontaneous urticaria (CSU). The prevalence of CSU in China is approximately 0.75%, with women being affected twice as likely as men (2). The pathogenesis of CSU is complex, with frequent relapses, severely impairing the quality of life of patients. Unlike other forms of urticaria, CSU occurs spontaneously without requiring physical or inducible triggers such as temperature changes, pressure, and exercise (3). According to national and international guidelines (1, 2), first-line treatment for CSU involves initiating standard doses of second-generation non-sedating H1 antihistamines (sgAH), with recommendations for dose escalation if an inadequate response is observed. However, despite the overall safety profile of sgAH (e.g., loratadine and cetirizine), mild fatigue or drowsiness may still occur in some patients during long-term management of CSU (4). To improve treatment adherence and address individualized needs, acupuncture therapy and other traditional Chinese medicine (TCM) interventions have emerged as valuable complementary methods in recent years. Recent studies suggest that acupuncture may modulate humoral and cellular immunity, regulate multiple signaling pathways, inhibit MC activation, and modulate gene expression and resting-state brain function, thus reducing allergic responses and alleviating itching symptoms (5, 6). Thus, acupuncture is considered a potential alternative therapy for CSU. However, the efficacy and safety of acupuncture in the treatment for CSU remain unclear, which warrants an updated and comprehensive systematic review. This review aims to systematically analyze the existing literature and conduct a meta-analysis to evaluate the efficacy and safety of acupuncture in the treatment for CSU and provide reliable evidence for clinical practice.

## 2 Materials and methods

This study adhered to the Preferred Reporting Items for Systematic Reviews and Meta-Analyses (PRISMA) guidelines. The study protocol was registered on PROSPERO (CRD42024557552), titled “The efficacy and safety of acupuncture for chronic spontaneous urticaria: A systematic review and meta-analysis of randomized clinical trials.”

### 2.1 Literature search

A comprehensive search of both Chinese and English databases was conducted to identify randomized clinical trials (RCTs) investigating acupuncture treatment for CSU. Chinese databases included CNKI, VIP, WanFang, and CBMDisc, whereas English databases included PubMed, Cochrane Library, Embase, and Web of Science. The search period spanned from the inception of each database until 20 August 2024, with no language restrictions. Additional searches of reference lists and grey literature were carried out to avoid missing relevant studies. Chinese search terms included “针刺” (acupuncture), “针灸” (acupuncture), “皮肤针” (dermal needling), “荨麻疹” (urticaria), and “随机对照试验” (randomized controlled trial). English search terms included “Acupuncture,” “Urticarias,” and “Urticarial Wheals.” Both subject terms and free-text terms were used in the search strategies, which were adapted to the characteristics of each database. A cross-search was performed to avoid omissions. For example, the search strategy for PubMed is shown in Table 1, with additional search strategies provided in Supplementary material 1.

**Table 1:** PubMed search strategy.

Steps	Retrieval formula
#1	“Acupuncture”[Mesh] OR “Pharmacopuncture”[All Fields]
#2	“Urticaria”[Mesh] OR “Urticarias”[All Fields] OR “Hives”[All Fields] OR “Urticarial Wheals”[All Fields] OR “Urticarial Wheal”[All Fields] OR “Wheals, Urticarial”[All Fields] OR “Wheal, Urticarial” [All Fields]
#3	“Randomized Controlled Trial” [Publication Type] OR “Controlled Clinical Trial” [All Fields] OR “Clinical Trial” [All Fields] OR “Clinical Study” [All Fields] OR “Randomized” [All Fields]
#4	#1 AND #2 AND #3

### 2.2 Inclusion and exclusion criteria (PICOS principle)

The inclusion and exclusion criteria for this study were based on the PICOS principle:

1. Study type: This systematic review and meta-analysis included RCTs in which acupuncture was the sole intervention. Non-randomized trials, observational studies, clinical protocols, animal experiments, case reports, and expert opinions were excluded. In the case of duplicate publications, only one study was included.
2. Participants: The participants were required to be diagnosed with CSU according to established international or domestic guidelines or expert consensus. There were no restrictions on nationality, ethnicity, gender, and age. However, patients in the acute phase or with severe liver, kidney, cardiovascular, cerebrovascular, or immune system diseases were excluded.
3. Interventions: The intervention in the treatment group was acupuncture as the primary therapy, with no restrictions on acupuncture type, course of treatment, acupoint selection, and frequency. Studies involving acupuncture, together with point injection, thread embedding, or non-acupuncture treatments such as oral medications, were excluded. Control group interventions included standard clinical medications, sham acupuncture, or placebo.
4. Outcome measures: The primary outcome measures included clinical efficacy, recurrence rate, TCM symptom scores, clinical symptom improvement, immune indicator changes, and adverse events, with the aim of evaluating the effectiveness and safety of acupuncture in the treatment for CSU.

### 2.3 Literature screening

All studies retrieved from Chinese and English databases were input into EndNote 20 for automatic deduplication. Two researchers independently screened the deduplicated studies. Initially, titles and abstracts were reviewed, and then, the full text of potentially eligible studies was further assessed. Reasons for exclusion were recorded, and any disagreements were resolved through discussion or adjudicated by a senior researcher. The screening results were cross-checked.

### 2.4 Data extraction

Two researchers independently extracted data from the included studies using a predefined extraction form. The extracted data included: (1) basic information: first author and publication year; (2) participant characteristics; (3) intervention details and treatment duration; (4) key factors related to the risk of bias; and (5) primary outcome data. If the required data were missing or unclear, the researchers attempted to contact the original authors or retrieve Supplementary information from the original studies.

### 2.5 Risk of Bias assessment (ROB2 tool)

The Risk of Bias 2 (ROB2) tool provided by the Cochrane Collaboration was used to assess the risk of bias in the included studies. The following six domains were evaluated: (1) bias in the randomization process; (2) bias due to deviations from intended interventions; (3) bias in outcome measurement; (4) bias due to missing outcome data; (5) bias in the selection of reported results; and (6) overall bias. Each study was categorized as having low, moderate, or high risk of bias based on these criteria.

### 2.6 Grading of Recommendations, Assessment, Development, and Evaluation

The Grading of Recommendations, Assessment, Development, and Evaluation (GRADE) framework was used to assess the quality of evidence for each outcome. The evidence was rated as high, moderate, low, or very low. Factors considered in the GRADE assessment included risk of bias, imprecision, inconsistency, indirectness, and publication bias.

### 2.7 Statistical methods

Meta-analysis was performed using RevMan 5.4 and Stata 15. To ensure broader applicability of acupuncture in various populations, heterogeneity was tested using the I^2^ statistic. An I^2^ value of less than 50% indicated low heterogeneity, in which case a fixed-effects model was used. For I^2^ values greater than 50%, indicating high heterogeneity, a more conservative random-effects model was applied. If there was significant clinical heterogeneity, sensitivity analyses and subgroup analyses were conducted to assess the stability of the results. Dichotomous outcomes used relative risk (RR) as the effect measure, whereas continuous outcomes used weighted mean difference. If different measurement scales were used, standardized mean difference (SMD) was used, all reported with 95% confidence intervals (CI). A *p*-value of less than 0.05 was considered statistically significant. Publication bias was assessed using funnel plots and Egger’s test in Stata 15.

## 3 Results

### 3.1 Characteristics of the included studies

**Figure 1:**
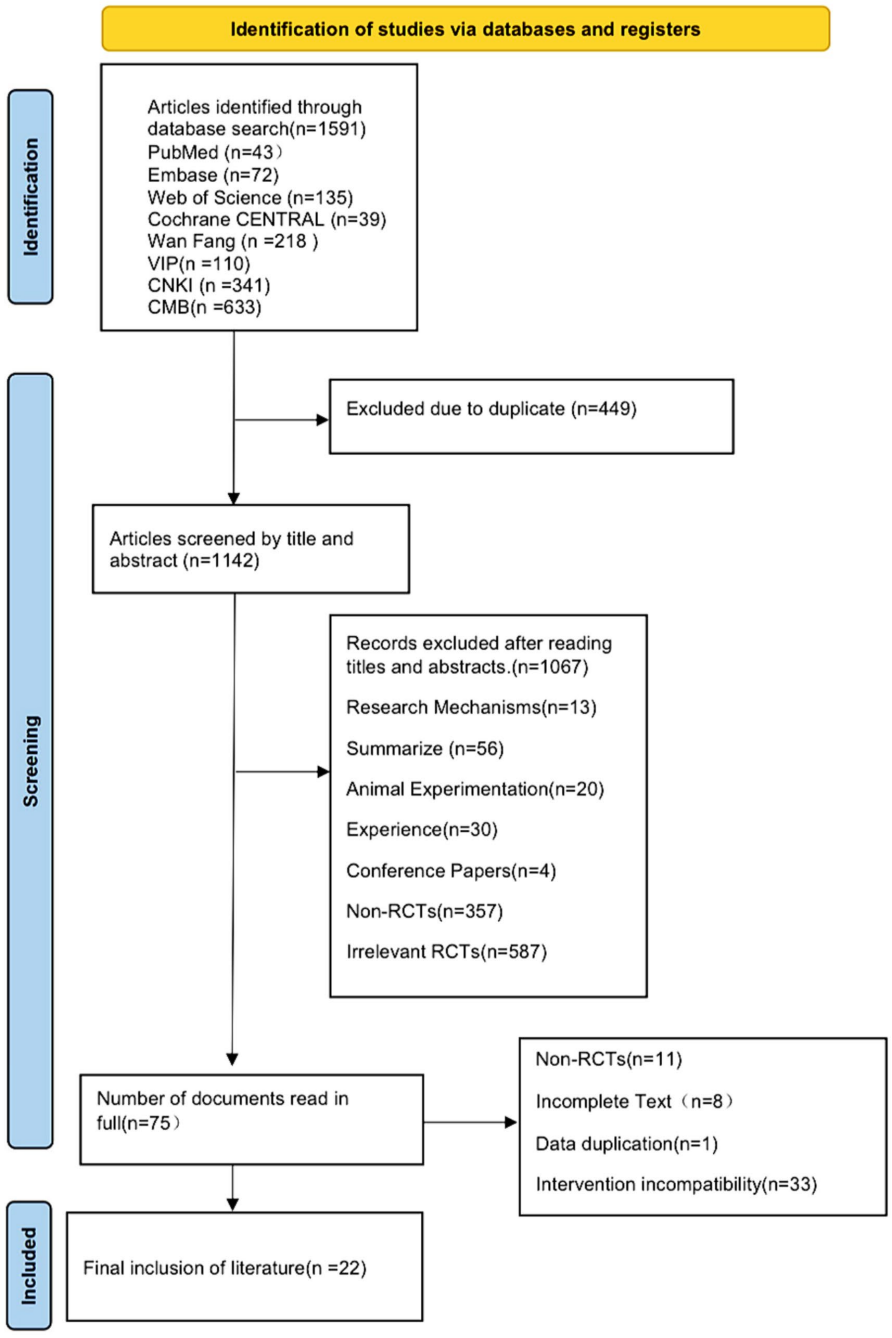
Flowchart of literature.

A total of 1,591 articles were initially identified, of which 449 duplicates were excluded. After screening titles and abstracts, 1,067 studies were further excluded as they were case reports, experimental studies (e.g., animal or cell studies), reviews, or meta-analyses, which did not align with the study interventions. After full-text review, 53 articles were further excluded as they were non-RCTs, lacked full text, or did not meet the inclusion criteria. A total of 1,569 articles were excluded. Finally, 22 studies (7–28) were included, with 20 published in Chinese (7–26) and 2 in English (27, 28). The detailed information on the literature search and selection process is presented in Figure 1, with the PRISMA flow diagram being provided in Supplementary material 2.

A total of 1,867 participants were included in the final statistical analysis, with 948 in the treatment group and 919 in the control group. The age range was 14–72 years, and the ratio of men to women was approximately 1:1.54. The overall mean age was 38.27 ± 3.56 years, with a mean age of 38.15 ± 3.71 years in the treatment group and 38.15 ± 3.71 years in the control group. All studies reported no statistically significant differences in baseline characteristics between the treatment and control groups (all *p* > 0.05), indicating that the groups were comparable. The diagnostic criteria in all studies adhered to the EAACI/GA2LEN/EDF/WAO Guideline for the Diagnosis and Management of Urticaria and the Chinese Guideline for the Diagnosis and Treatment of Urticaria (1).

### 3.2 Results of literature quality assessment

The included studies were subjected to the risk of bias assessment. Regarding bias in the randomization process, all 22 studies were RCTs. Four studies (7, 11, 17, 23) used random envelope allocation, eight studies (16, 18–21, 26–28) generated random sequences using a random number table, nine studies (9, 10, 12, 14, 15, 22, 24, 25, 29) mentioned randomization without specifying the method used, and one study (8) did not mention the randomization method. In terms of allocation concealment and blinding, due to the nature of the intervention, it was nearly impossible to blind the practitioners. However, five studies (11, 25–28) followed blinding of participants and assessors and concealed the allocation scheme.

**Figure 2:**
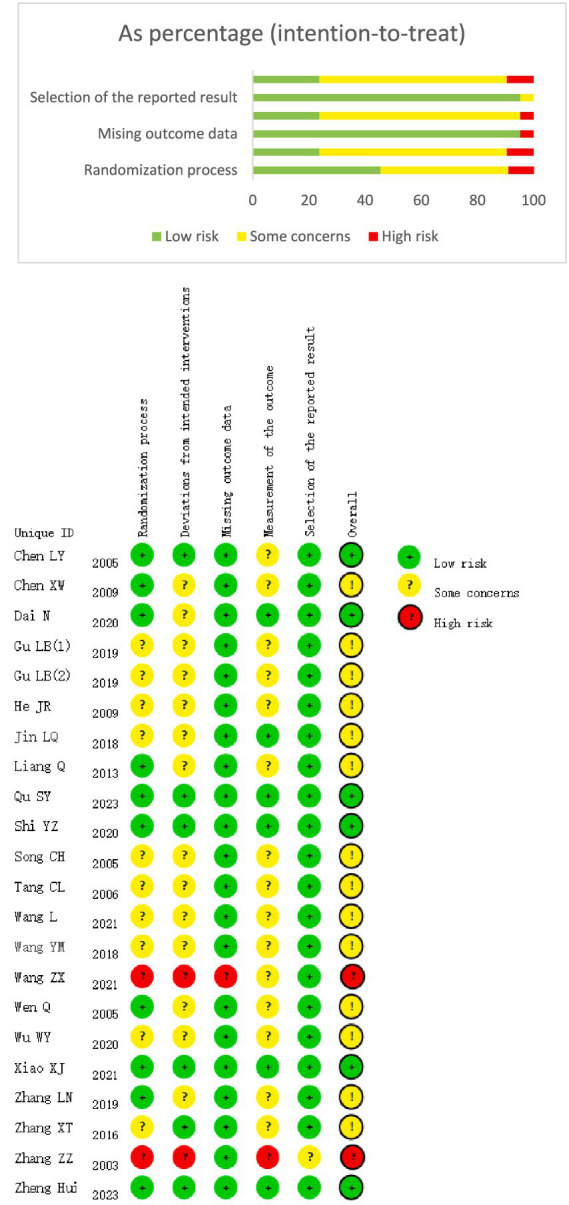
**(A)** Quality of detailed articles. **(B)** Quality of overall articles.

Regarding incomplete outcome data, 13 studies (7, 10, 12–17, 19–22, 24) reported no loss to follow-up, with complete data sets, whereas nine studies (8, 9, 11, 18, 23, 25–28) reported a total of 22 dropouts in the experimental group and 44 dropouts in the control group (Figure 2).

### 3.3 Meta-analysis results

#### 3.3.1 Efficacy rate

**Figure 3:**
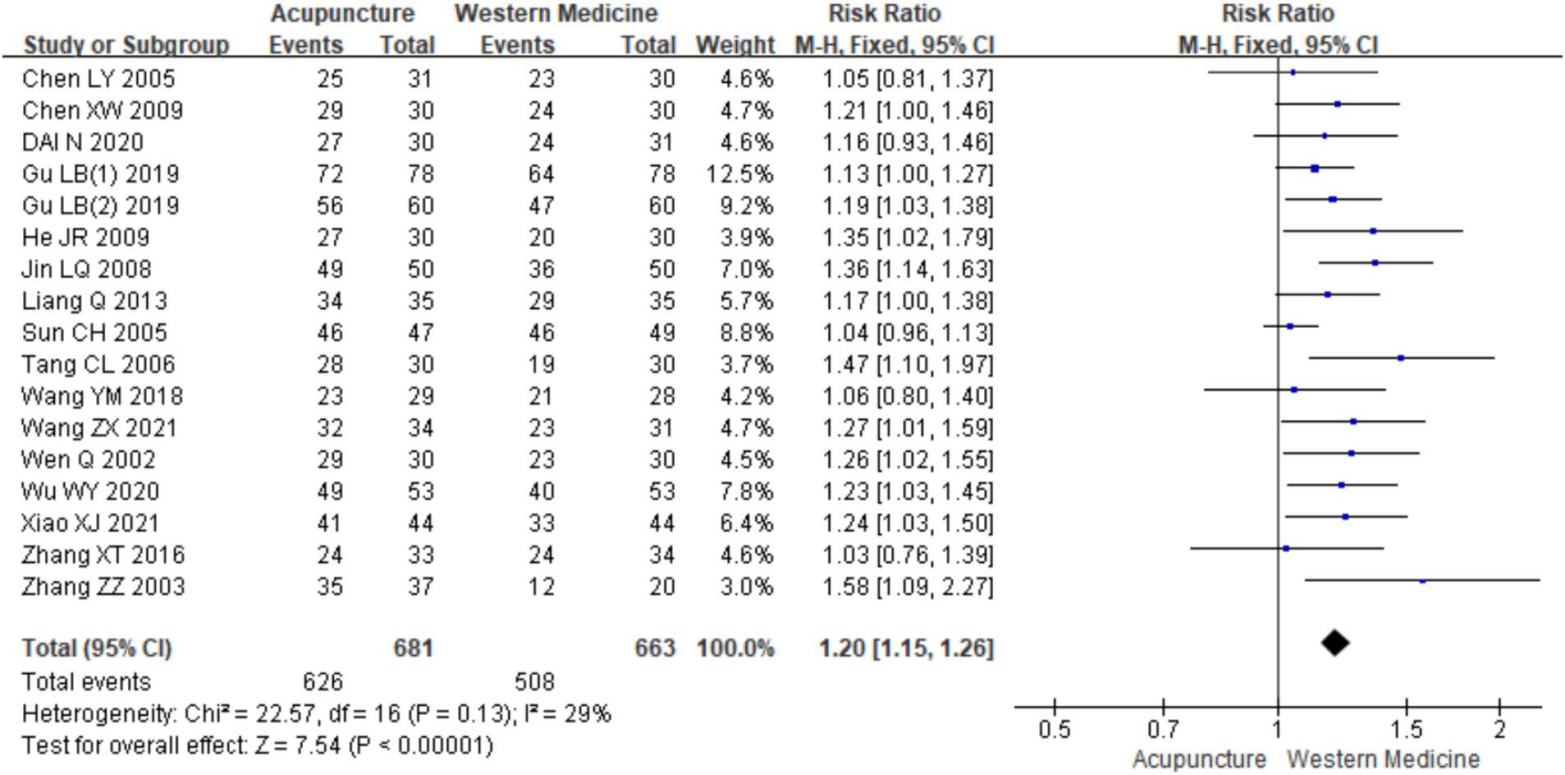
Forest plot of efficiency.

**Figure 4:**
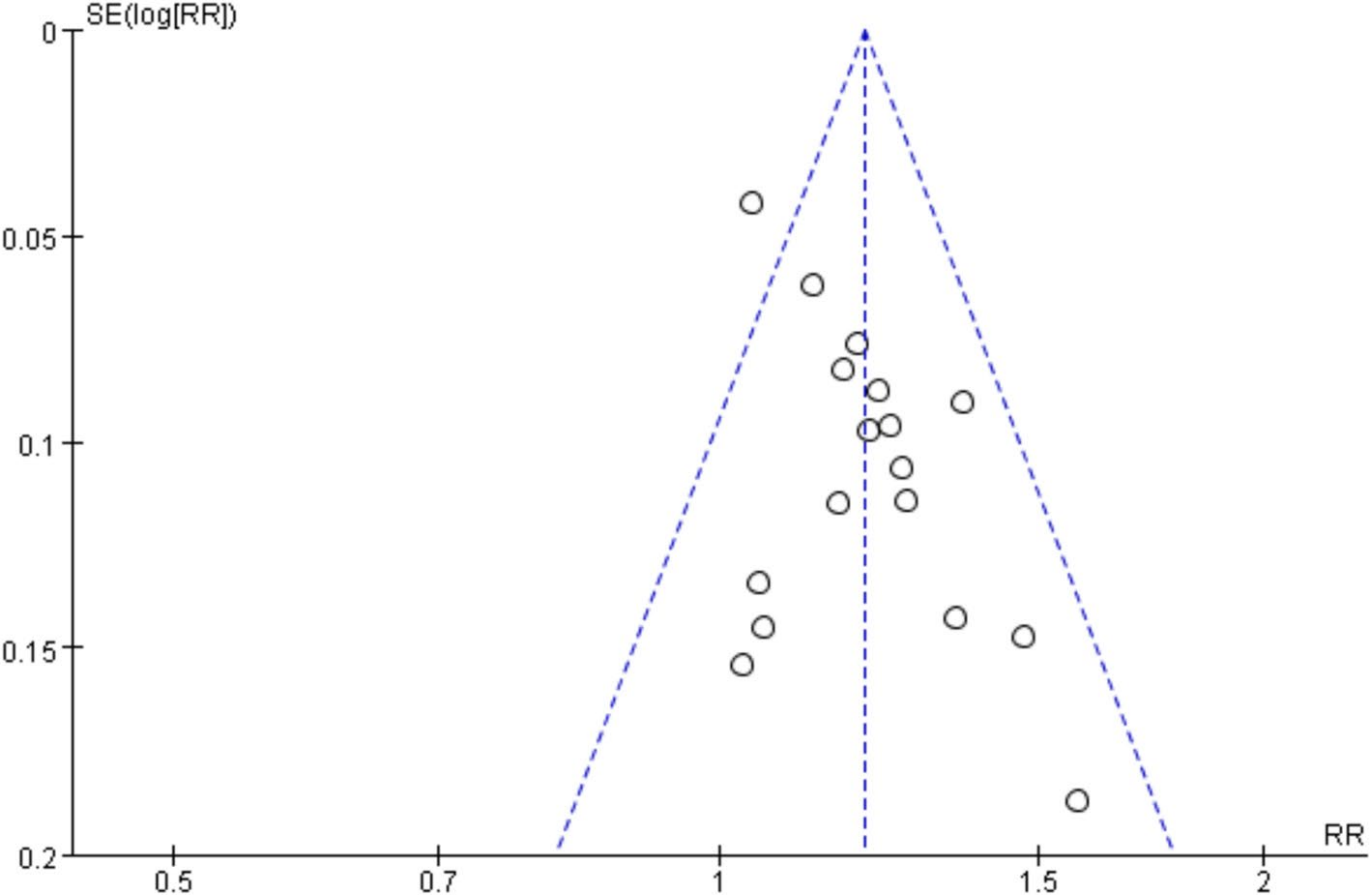
Funnel plot of efficiency.

**Figure 5:**
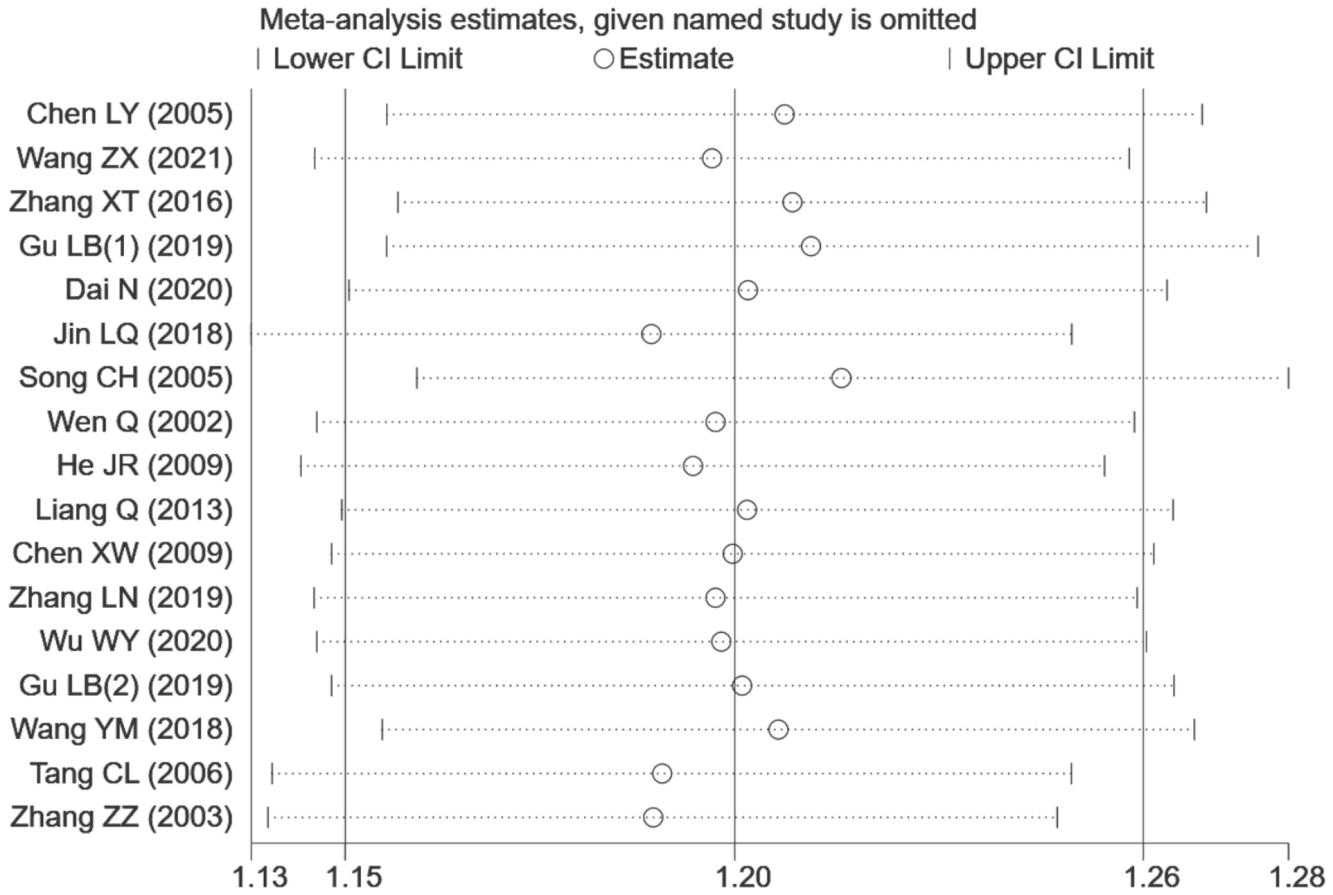
Sensitivity analysis of efficiency.

Seventeen RCTs (7–17, 19–22, 24, 26) involving a total of 1,344 patients were included in the analysis of efficacy rate. All studies followed the “Guideline for the Diagnosis and Treatment of Urticaria” from the Chinese Society of Dermatology (30). A heterogeneity test was conducted (chi^2^ = 22.57, *p* = 0.13, and I^2^ = 29%), which indicated low heterogeneity. Thus, a fixed-effects model was used for meta-analysis. The results showed a statistically significant difference in the efficacy rate favoring acupuncture over conventional treatment (oral Western medicine) and sham acupuncture groups [RR = 1.20, 95% CI (1.15, 1.26), *p* < 0.001], suggesting that acupuncture was more effective in treating CSU (Figure 3). Publication bias was evaluated using a funnel plot, which appeared roughly symmetrical on both sides (Figure 4). Egger’s test was conducted for further quantification (*t* = 3.11, *p* = 0.007), which indicated the presence of some publication bias (for details on Egger’s test for efficacy rate data, refer to Supplementary material 3, Egger’s test 1.2). Sensitivity analysis (Figure 5) showed that the structure of the included studies remained stable (Table 2).

**Table 2:** Basic characteristics of the included articles.

Author	Years	Total_N	Experimental Group				Control Group				Intervene	Course of treatment	Outcome
			Acupuncture_N	Genders (men/women)	Age	Course	Control_N	Genders (men/women)	Age	Course			
Chen LY (7)	2005	61	31	11/20	33.5 ± 12.6	1.54 ± 1.12	30	13/17	32.9 ± 10.8	1.49 ± 1.16	Acupuncture VS Loratadine	4 weeks	1
Chen XW (17)	2009	60	30	12/18	-	-	30	15/15	-	-	Acupuncture VS Loratadine	4 weeks	1
Dai N (11)	2020	61	30	11/19	38.33 ± 10.41	12.00(6.75,21.00)	31	11/20	38.61 ± 10.43	15.00(7.00,27.00)	Acupuncture VS Loratadine	4 weeks	1.2.3.5.9.11.16
Gu LB(1) (10)	2019	156	78	48/30	37.14 ± 11.37	3.49 ± 1.18	78	50/28	37.28 ± 11.91	3.41 ± 1.09	Acupuncture VS Loratadine	4 weeks	1.3.5.12.13.14
Gu LB(2) (19)	2019	120	60	26/34	40 ± 12	16.35 ± 12.08	60	28/32	41 ± 12	17.01 ± 13.11	Acupuncture VS Loratadine	6 weeks	1.12.13.14
He JR (15)	2009	60	30	13/17	35.9 ± 9.1	6.0 ± 4.2	30	16/14	39.9 ± 10.6	6.8 ± 5.0	Acupuncture VS Loratadine	4 weeks	1
Jin LQ (12)	2018	100	50	28/22	43.43 ± 14.84	6.8 ± 1.9	50	30/20	43.60 ± 14.33	5.1 ± 2.1	Acupuncture VS Loratadine	30 days	1.3.5.6.
Liang Q (16)	2013	70	35	17/18	-	-	35	15/20	-	-	Acupuncture VS Loratadine	8 weeks	1.2.3.4.5.6.7.11
Qu SY (23)	2023	60	32	11/21	45.01 ± 11.76	40.50(12.00, 120.00)	28	11/17	43.57 + 11.66	48.00(20.00, 99.50)	Acupuncture VS Sham Acupuncture	4 weeks	5.9.11
Shi YZ (28)	2023	80	41	14/27	38.2 ± 12.5	38.4 ± 60.0	39	11/28	39.7 ± 13.0	39.5 ± 44.9	Acupuncture VS Sham Acupuncture	2 weeks	5.7.8.11
Song CH (13)	2005	96	47	19/28	35.5 ± 12.6	-	49	14/35	37.8 ± 13.4	-	Acupuncture VS Cimetidine	4 weeks	1.3.4.5.
Tang CL (22)	2006	60	30	-	-	-	30	-	-	-	Acupuncture VS Loratadine	2 weeks	1
Wang L (25)	2021	49	24	6/18	31.83 ± 8.26	3.04 ± 3.22	25	7/18	32.36 ± 10.78	3.28 ± 3.86	Acupuncture VS Sham Acupuncture	4 weeks	5.7.8.11
Wang YM (21)	2018	57	29	5/24	43.5 ± 10.66	12.18 ± 8.87	28	6/22	43.61 ± 12.59	5.21 ± 0.67	Acupuncture VS Loratadine	6 weeks	1.2.11
Wang ZX (8)	2021	65	34	-	-	-	31	-	-	-	Acupuncture VS Cimetidine	-	1.7.8.11
Wen Q (14)	2002	60	30	12/18	38.5 ± 2.007	29.10 ± 2.307	30	15/15	39.10 ± 1.830	32.70 ± 3.048	Acupuncture VS Loratadine	2 weeks	1.2.8.9.11
Wu WY (19)	2020	106	53	25/28	42 ± 5	17 ± 5	53	27/26	42 ± 6	18 ± 6	Acupuncture VS Loratadine	8 weeks	1.3.4.5.6.7.12.13.14
Xiao XJ (26)	2021	134	67	15/52	33(28,48)	24(6,61)	67	18/49	32(27,47)	28(8,62)	Acupuncture VS Sham Acupuncture	4 weeks	5.7.8.11
Zhang LN (18)	2019	88	44	-	40.76 ± 8.86	22.54 ± 6.57	44	-	41.79 ± 7.49	23.21 ± 7.70	Acupuncture VS Loratadine	6 weeks	1.2.12.13.14.16
Zhang XT (9)	2016	67	33	4/29	35.42 ± 9.53	14.12 ± 1.41	34	10/24	31.82 ± 8.97	14.06 ± 1.39	Acupuncture VS Loratadine	-	1.14.15
Zhang ZZ (24)	2003	57	37	15/22	-	-	20	13/7	-	-	Acupuncture VS Cimetidine and Chlorphenamine Maleate	10 days	1
Zheng Hui (27)	2023	200	103	25/85	38.4(36.0 ± 40.9)	-	97	31/79	39.2(36.7–41.7)	-	Acupuncture VS Sham Acupuncture	4 weeks	5.7.8.11

Outcome: 1. Total effective rate. 2. Recurrence rate. 3. Number of wheals. 4. Wheal diameter [size]. 5. Itching severity. 6. Duration of attacks. 7. Dermatology Life Quality Index [DLQI]. 8. Hamilton Depression Rating Scale [HAMD]. 9. Chronic Urticaria Quality of Life. Questionnaire [CU-Q2oL] 10.Itching Visual Analogue Scale [VAS] 11.Urticaria Activity Score [UAS] 12.Serum IgE 13.IFN-γ [Interferon-gamma] 14.IL-4 levels 15.European MILOR score 16.Traditional Chinese Medicine syndrome score.

**Figure 6:**
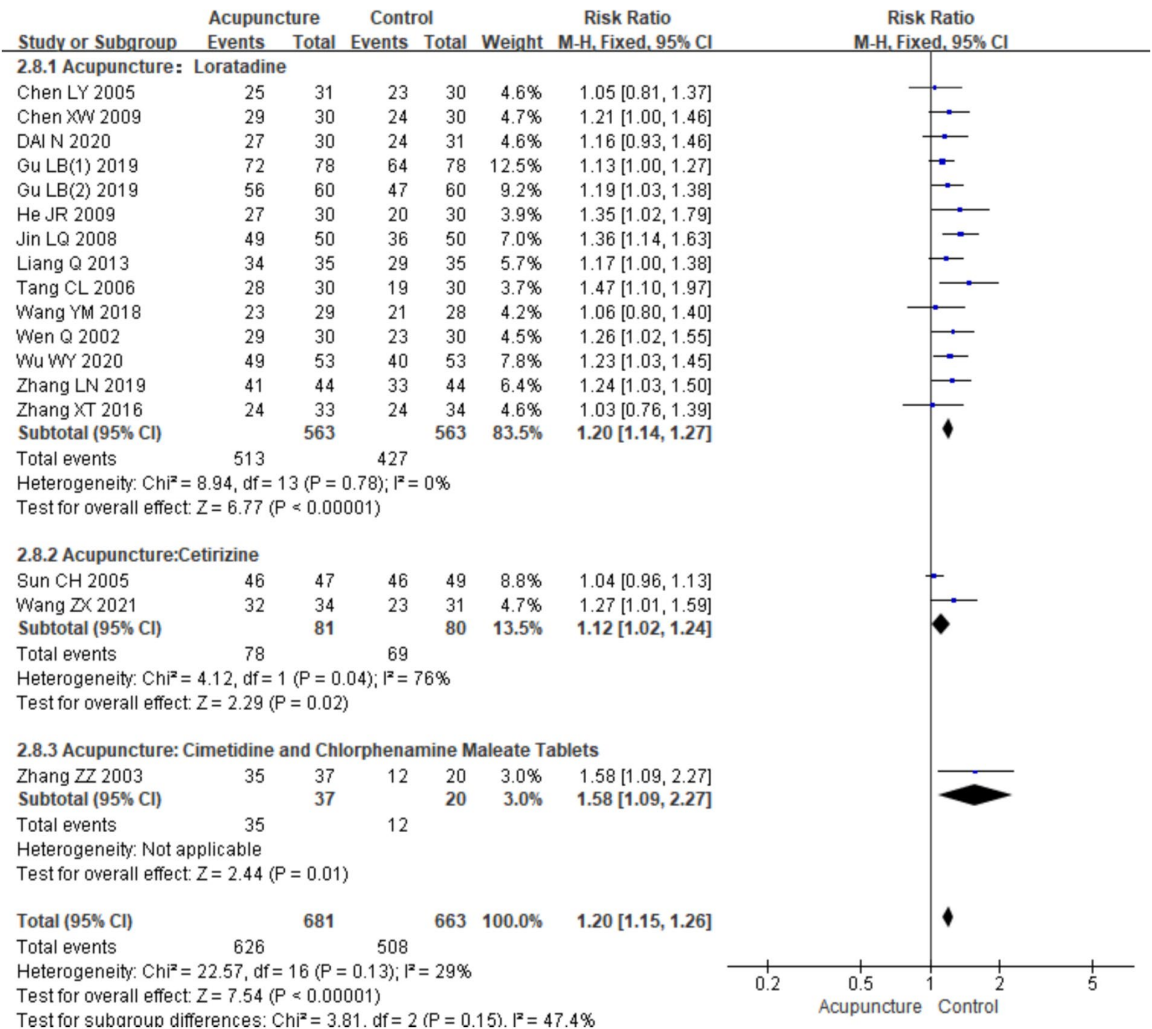
Forest plot of subgroup analyses of the efficacy of different intervention methods.

In the subgroup meta-analysis based on different control interventions, efficacy rate data were categorized into three subgroups: acupuncture versus loratadine, acupuncture versus cetirizine, and acupuncture versus cimetidine and chlorpheniramine. Acupuncture vs. loratadine: This subgroup included 14 studies (7, 9–12, 14–22). No heterogeneity was observed in these studies (chi^2^ = 8.49, *p* = 0.78,I^2^ = 0%). The combined results indicated a statistically significant difference in favor of acupuncture [RR = 1.20, 95% CI (1.14, 1.27), *p* < 0.001]. Acupuncture vs. cetirizine: This subgroup included only two studies (8, 13). Heterogeneity was observed (chi^2^ = 4.12, *p* = 0.04, I^2^ = 76%). The combined results also showed a statistically significant difference in favor of acupuncture [RR = 1.20, 95% CI (1.14, 1.27), p < 0.001]. Acupuncture vs. cimetidine and chlorpheniramine: This subgroup had only one study (24), and a qualitative analysis was conducted. The results showed a statistically significant difference [RR = 1.58, 95% CI (1.09, 2.27), *p* = 0.01] (Figure 6). These results indicate that acupuncture is more effective than these conventional treatments across all subgroups although the level of heterogeneity varied between them (Table 3).

**Table 3:** Acupoint frequency.

Author	Year	Acupoints	Frequency Descriptions
Chen LY (7)	2005	Primary Acupoints:RN12(Zhongwan), RN10(Xiawan), RN6(Qihai), BL26(Guanyuan), Supplementary Acupoints: ST24(Sanyinjiao), ST26(Wailing), SP15(Daheng)	5 times per week
Chen XW (17)	2009	LI11(Quchi), SP10(Xuehai), ST36(Zusanli) and SP06(Sanyinjiao); Wind-Heat Affecting the Exterior:Add DU14(Dazhui); Wind-Cold Constraining the Exterior: Add BL13(Feishu); Stomach and Intestines Excess Heat: Add LI4(Hegu), ST44(Neiting); Blood Deficiency with Wind-Dryness: Add (Geshu), BL20(Pishu).	2 times per week
Dai N (11)	2020	Root-Cutting Therapy Acupoints.	2 times per week
Gu LB(1) (10)	2019	LI15(Jianyu), LI5(Yangxi)	
Gu LB(2) (19)	2019	LI11(Quchi), LI15(Jianyu), LI10(Shousanli)	5 times per week
He JR (15)	2009	Encircling Needling; Rash on the Upper Body: Add LI11(Quchi), LI4(Hegu) Rash on the Lower Body: Add SP10(Xuehai), ST36(Zusanli), SP06(Sanyinjiao); Rash on the Whole Body: Add GB20(Fengchi), DU14(Dazhui).	5 times per week
Jin LQ (12)	2018	Four Needles around the Navel Combined with Abdominal Acupoints. To Guide Qi Back to Its Origin. Abdominal Four Gates, ST25(Tianshu), SP15(Daheng).	Once daily for the first 3 days, then once every 2 days
Liang Q (16)	2013	LI11(Quchi), SJ5(Waiguan), LI4(Hegu), ST36(Zusanli), SP10(Xuehai), SP06(Sanyinjiao), LR3(Taichong); Wind-Heat Affecting the Exterior: Add GB20(Fengchi), DU16(Fengfu); Wind-Cold Constraining the Exterior: Add DU14(Dazhui), BL12(Fengmen); Blood Deficiency with Wind-Dryness: Add BL17(Geshu), LR5(Ligou).	2 times per week
Qu SY (23)	2023	both sides:HT7(Shenmen), PC6(Neiguan), LI4(Hegu), LI11(Quchi), ST36(Zusanli), SP06(Sanyinjiao).	3 times per week
Shi YZ (28)	2023	DU24(Shenting), DU20(Baihui), LI11(Quchi), RN12(Zhongwan), ST25(Tianshu), SP10(Xuehai), ST36(Zusanli), SP06(Sanyinjiao).	5 times per week
Song CH (13)	2005	LI11(Quchi).	6 times per week
Tang CL (22)	2006	Primary Acupoints: BL13(Feishu), LI4(Hegu), SP10(Xuehai), ST36(Zusanli), BL20(Pishu), BL18(Ganshu). Wind-Cold Syndrome: Add DU14(Dazhui), LI11(Quchi), GB20(Fengchi), LU7(Lieque); Wind-Heat Syndrome: Add DU14(Dazhui), LI11(Quchi), LU6(Kongzui), BL11(Dashu); Stomach and Intestines Excess Heat Syndrome: Add ST34(Liangqiu), ST44(Neiting); Qi and Blood Deficiency Syndrome: Add DU14(Dazhui), LI11(Quchi), BL26(Guanyuan), RN6(Qihai), RN3(Zhongji); Disharmony of the Chong and Ren Channels: DU14(Dazhui), LI11(Quchi), ST32(Futu), SP06(Sanyinjiao).	Once daily
Wang L (25)	2021	LI11(Quchi), SP10(Xuehai), SP06(Sanyinjiao), ST36(Zusanli), ST25(Tianshu), RN12(Zhongwan), HT7(Shenmen).	5 times per week for the first 2 weeks, 3 times per week for the 3rd and 4th weeks
Wang YM (21)	2018	HT8(Shaofu), HT7(Shenmen), HT5(Tongli), PC7(Daling), LI11(Quchi), LI4(Hegu), SP10(Xuehai), ST36(Zusanli), SP06(Sanyinjiao).	Once every 2 days, 3 times per week
Wang ZX (8)	2021	GB20(Fengchi), Auricular Points (Heart, Lung, Shenmen), HT7(Shenmen), RN12(Zhongwan), ST25(Tianshu), BL26(Guanyuan), LI11(Quchi), SP10(Xuehai), ST36(Zusanli), SP06(Sanyinjiao)	Once every 2 days, 3 times per week
Wen Q (14)	2002	Primary Acupoints: Qihai, Guanyuan, both sides of RN6(Qihai)(Guanyuan)(Guanyuan), BL26(Guanyuan)(Sanyinjiao). Supplementary Acupoints: Wind-Heat Affecting the Exterior: Add DU14(Dazhui), using the dispersing method. Wind-Cold Constraining the Exterior: Add BL12(Fengmen), using the dispersing method. Stomach and Intestines Damp-Heat: Add ST44(Neiting), using the dispersing method. Qi and Blood Deficiency: Add BL20(Pishu), BL23(Shenshu), using the tonifying method.	6 times per week
Wu WY (19)	2020	GB20(Fengchi), BL12(Fengmen), DU16(Fengfu), SI12(Bingfeng), SJ17(Yifeng), GB31(Fengshi).	5 times per week
Xiao XJ (26)	2021	RN12(Zhongwan), both sides of LI11(Quchi), HT7(Shenmen), ST25(Tianshu), SP10(Xuehai), ST36(Zusanli), SP06(Sanyinjiao)	5 times per week for the first 2 weeks, 3 times per week for the 3rd and 4th weeks
Zhang LN (18)	2019	GB20(Fengchi), BL12(Fengmen), DU16(Fengfu), SI12(Bingfeng), SJ17(Yifeng), GB31(Fengshi)	5 times per week
Zhang XT (9)	2016	Wood Acupoints <Dong’s Extraordinary Points>	Once every 3 days
Zhang ZZ (24)	2003	LI15(Jianyu), LI11(Quchi), SP10(Xuehai), SP06(Sanyinjiao), ST36(Zusanli)	Once daily
Zheng Hui (27)	2023	LI11(Quchi), SP10(Xuehai), ST36(Zusanli), ST25(Tianshu), SP06(Sanyinjiao), HT7(Shenmen), RN12(Zhongwan).	10 times in the first 2 weeks, 6 times in the next 2 weeks

**Figure 7:**
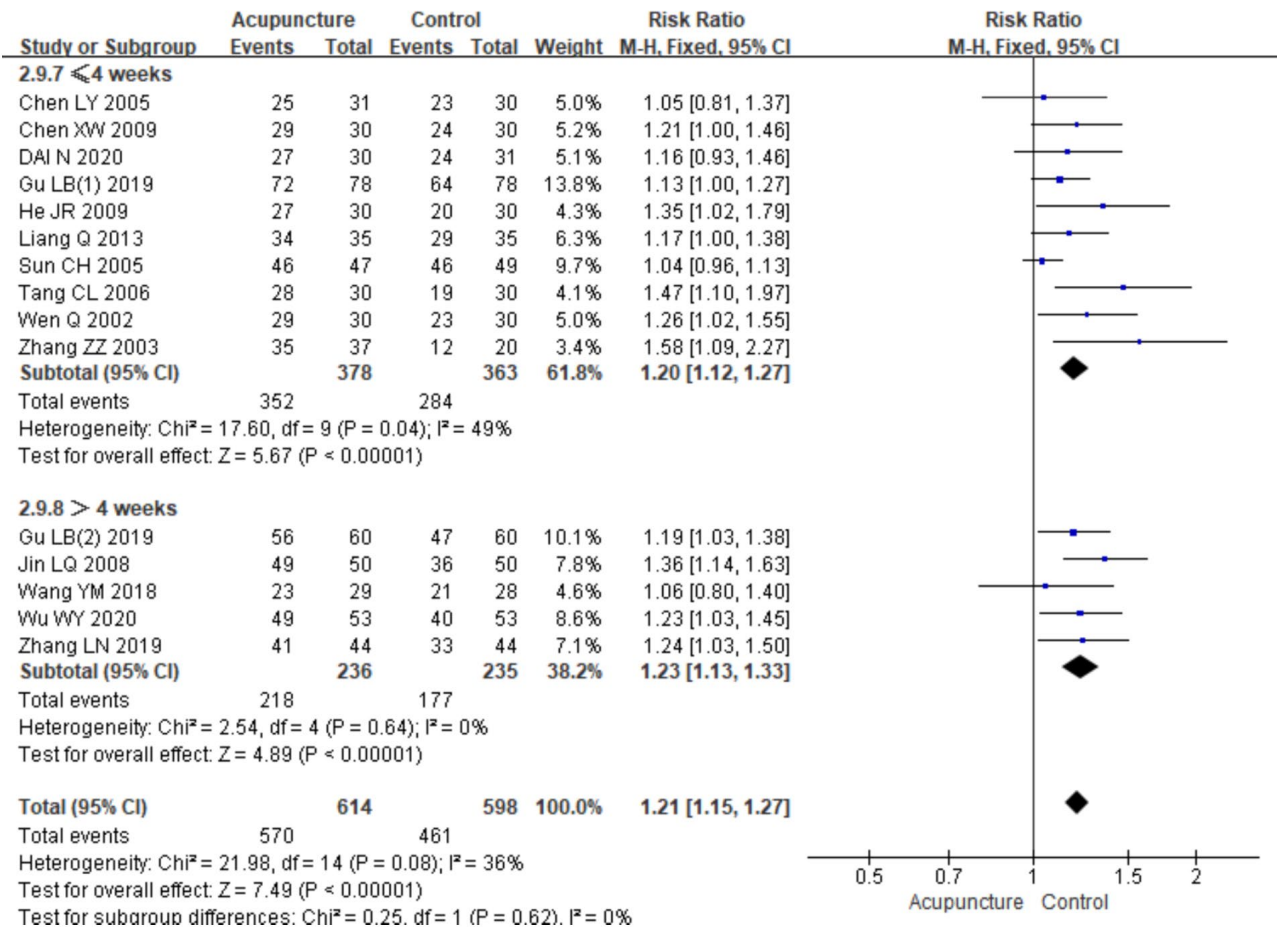
Forest plots of subgroup analyses of efficacy across treatment cycles.

A subgroup analysis based on the duration of treatment was also conducted. A total of 15 RCTs were included, with 10 studies (7, 10, 11, 13–17, 22, 24) having a treatment duration of ≤4 weeks and five studies (12, 18–21) with a treatment duration of >4 weeks. In the subgroup with a treatment duration of ≤4 weeks, low heterogeneity was observed (chi^2^ = 17.60, *p* = 0.04, I^2^ = 49%). The combined analysis showed a statistically significant difference favoring acupuncture [RR = 1.20, 95% CI (1.12, 1.27), *p* < 0.001]. In the subgroup with a treatment duration of >4 weeks, no heterogeneity was observed (chi^2^ = 2.54, *p* = 0.64, I^2^ = 0%). The combined analysis also showed a statistically significant difference favoring acupuncture [RR = 1.23, 95% CI (1.13, 1.33), *p* < 0.001] (Figure 7). These results indicate that acupuncture is effective regardless of the treatment duration, with slightly better outcomes observed in the subgroup with a treatment duration of >4 weeks.

#### 3.3.2 Recurrence rate

**Figure 8:**
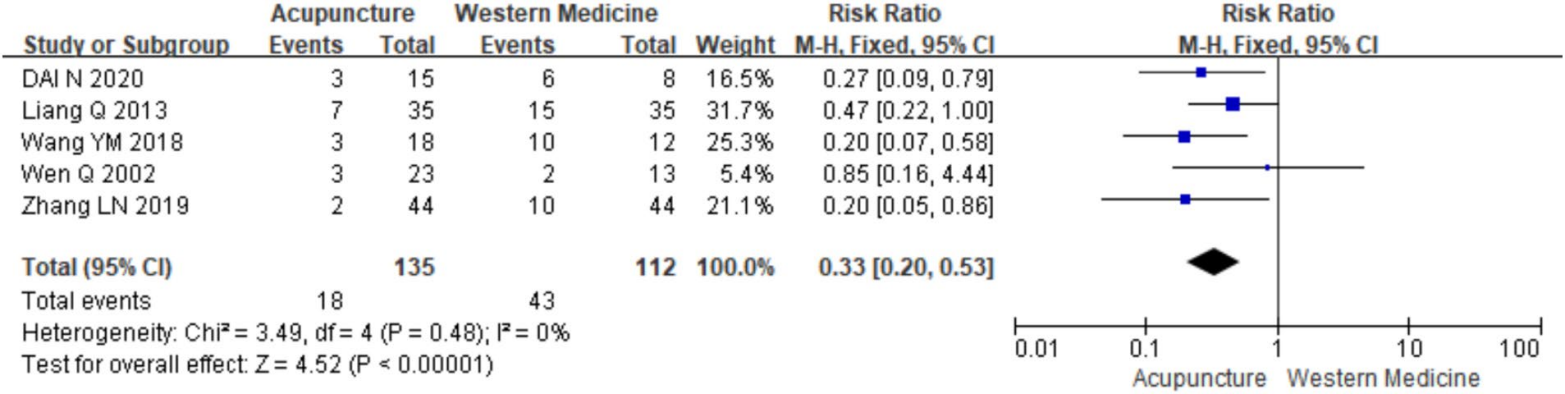
Forest plot of recurrence rates.

Five studies (11, 14, 16, 18, 21) involving 247 patients were included in the analysis of recurrence rates (urticaria relapse post-intervention withdrawal). A heterogeneity test was conducted (chi^2^ = 3.49, *p* = 0.48, I^2^ = 0%), which showed low heterogeneity. Therefore, a fixed-effects model was used for meta-analysis. The results showed a statistically significant difference favoring acupuncture in reducing recurrence rates compared with the control group treated with loratadine [RR = 0.33, 95% CI (0.20,0.53), *p* < 0.001], suggesting that acupuncture is associated with a lower recurrence rate for CSU (Figure 8). The funnel plot appeared symmetrical, and Egger’s test showed no significant publication bias (*t* = −0.21, *p* = 0.845) (for details on the funnel plot and Egger’s test, refer to Supplementary material 3).

#### 3.3.3 Urticaria activity score

**Figure 9:**
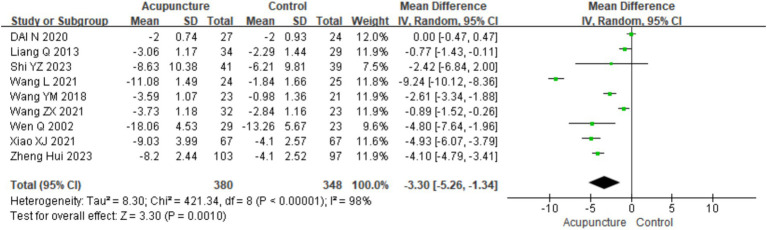
Forest plot of UAS7.

A total of nine studies (8, 11, 14, 16, 21, 25–28) involving 728 patients reported urticaria activity score (UAS7) scores. A heterogeneity test was conducted (chi^2^ = 421.34, *p* < 0.001, I^2^ = 98%), which indicated significant heterogeneity. Therefore, a random-effects model was used for meta-analysis. The combined results showed a statistically significant difference favoring acupuncture [mean difference (MD) = −3.30, 95% CI (−5.26, −1.34), *p* = 0.001], indicating that acupuncture resulted in a higher reduction in UAS7 scores than oral loratadine, cetirizine, or sham acupuncture, which indicated the better efficacy of acupuncture in reducing urticaria activity (Figure 9). The funnel plot appeared symmetrical, suggesting no significant publication bias, and Egger’s test further confirmed the same (t = −1.28, *p* = 0.243) (for details on the funnel plot and Egger’s test, refer to Supplementary material 3, UAS7 Funnel plot 1 and Egger’s test 1.2).

**Figure 10:**
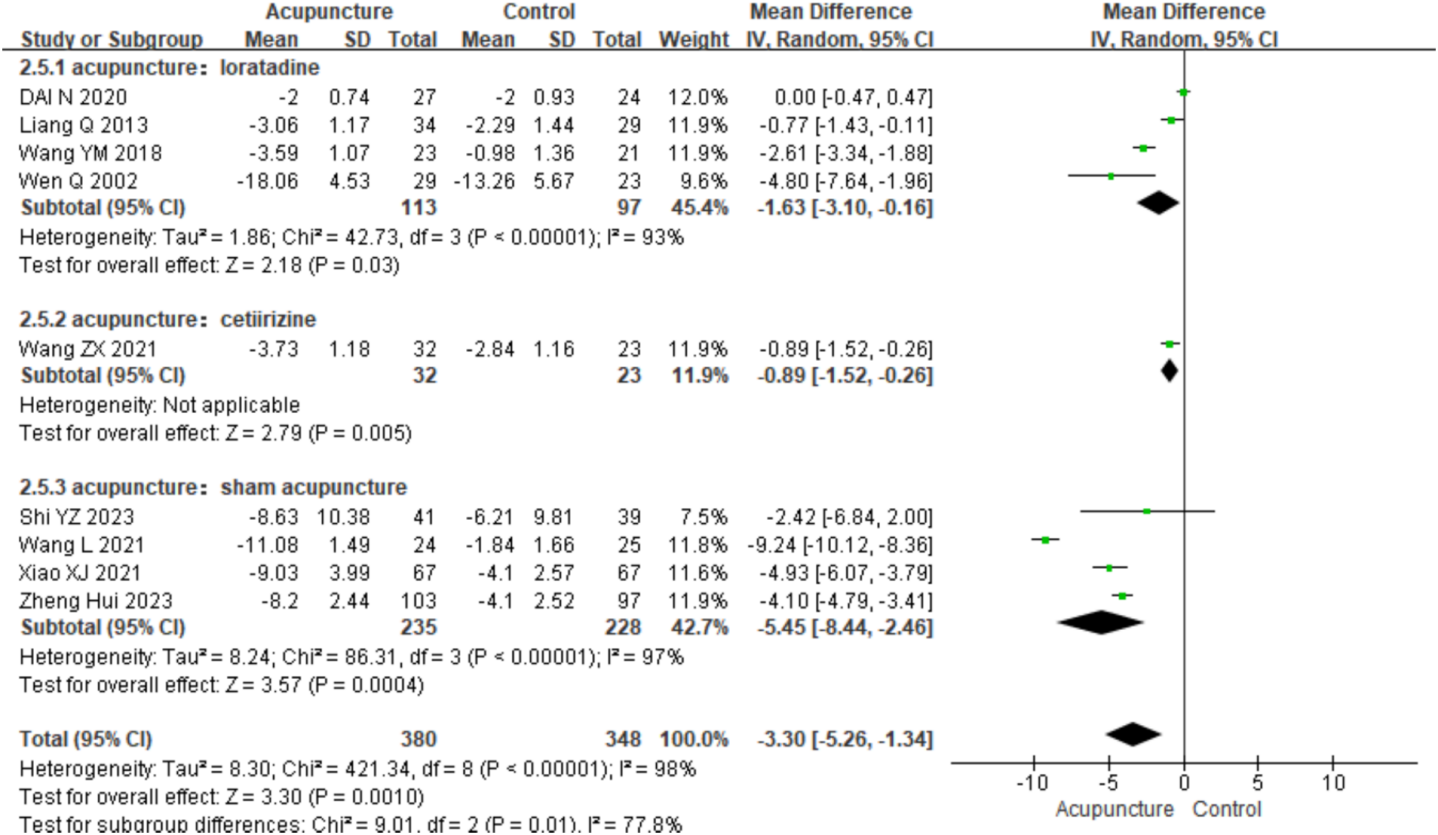
Forest plot of subgroup analyses of UAS7 of different intervention methods.

Using UAS7 as an indicator, a subgroup analysis was conducted based on different control interventions: acupuncture vs. loratadine, acupuncture vs. cetirizine, and acupuncture vs. sham acupuncture. Acupuncture vs. loratadine: Four studies (11, 14, 16, 21) were included. This subgroup showed significant heterogeneity (chi^2^ = 42.73, *p* < 0.001, I^2^ = 93%). The combined analysis indicated a statistically significant difference favoring acupuncture [MD = −1.63, 95% CI (−3.10, −0.16), *p* = 0.03]. Acupuncture vs. cetirizine: Only one study (8) was included in this subgroup. A qualitative analysis was performed, showing a statistically significant difference favoring acupuncture [MD = −0.89, 95% CI (−1.52, −0.26), *p* < 0.001]. Acupuncture vs. sham acupuncture: Four studies (25–28)were included in this subgroup, which also showed significant heterogeneity (chi^2^ = 42.73, *p* < 0.001, I^2^ = 93%). The combined results revealed a statistically significant difference favoring acupuncture [MD = −5.45, 95% CI (−8.44, −2.46), *p* < 0.001] (Figure 10). Despite the positive findings, high heterogeneity persisted after subgroup analysis, indicating that other factors may contribute to the variability in results. Further investigation is needed to identify the sources of this heterogeneity.

#### 3.3.4 Dermatology life quality index

**Figure 11:**
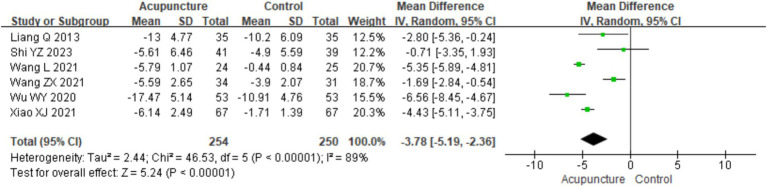
Forest plot of DLQI.

A total of six studies (8, 16, 19, 25, 26, 28) involving 504 patients reported Dermatology Life Quality Index (DLQI) scores. Heterogeneity testing showed significant heterogeneity (chi^2^ = 46.53, *p* < 0.001, I^2^ = 89%). Therefore, a random-effects model was used for meta-analysis. The combined results revealed a statistically significant difference favoring acupuncture, with acupuncture showing lower DLQI scores than loratadine, cetirizine, and sham acupuncture [MD = −3.78, 95% CI(−5.19, −2.36), *p* < 0.001], indicating that acupuncture resulted in a greater improvement in quality of life in patients with CSU (Figure 11). A funnel plot assessment for publication bias showed symmetry on both sides. Further analysis using Egger’s test indicated no significant publication bias (*t* = 1.12, *p* = 0.293) (refer to Supplementary material 3 for DLQI funnel plot 1 and Egger’s test 1.2).

**Figure 12:**
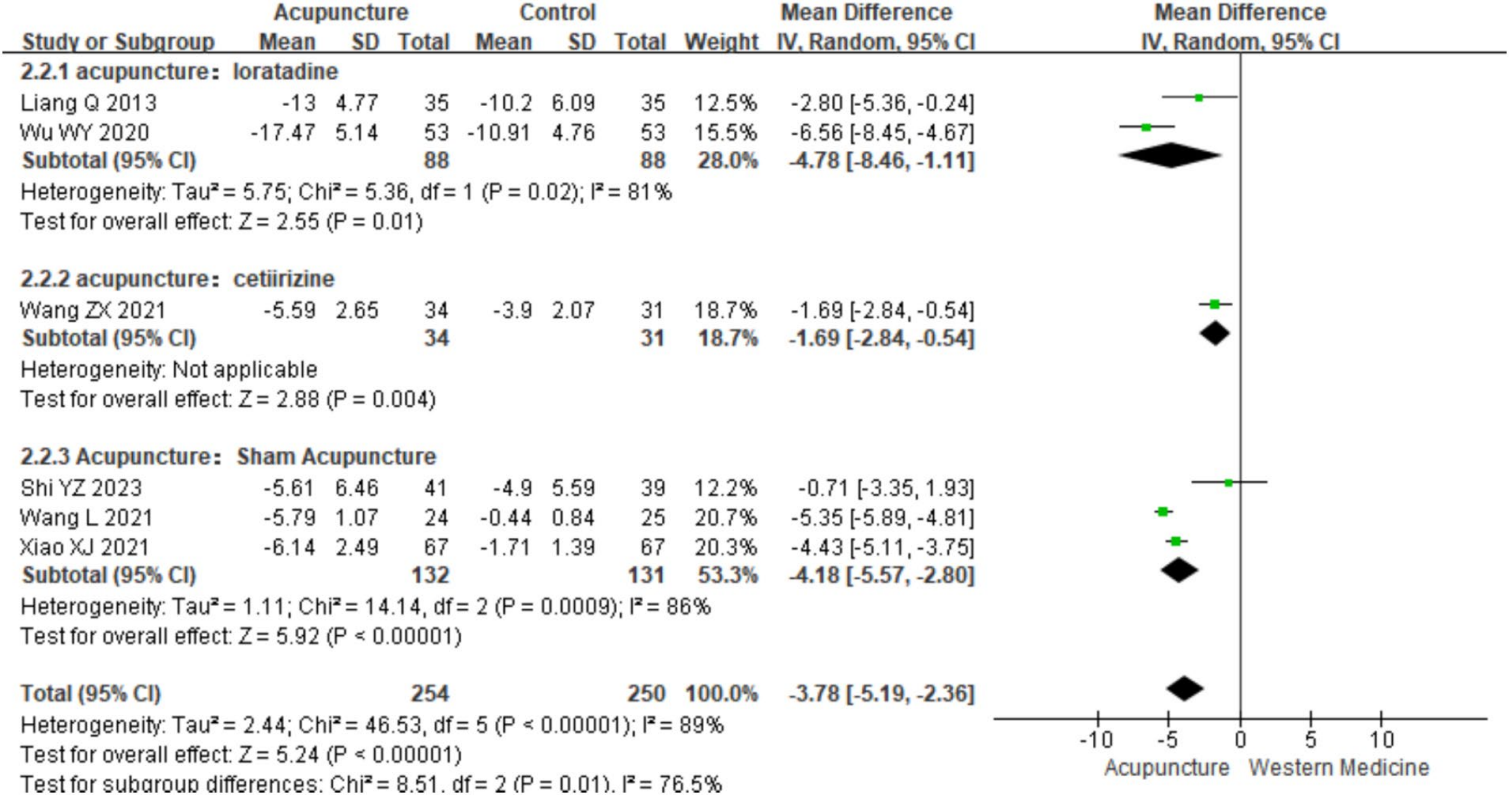
Forest plot of subgroup analyses of DLOI of different intervention methods.

Based on control group interventions, a subgroup analysis was conducted, categorizing the studies into three groups: acupuncture vs. loratadine, acupuncture vs. cetirizine, and acupuncture vs. sham acupuncture. Acupuncture vs. loratadine: This subgroup included two studies (16, 19). Significant heterogeneity was observed in this subgroup (chi^2^ = 5.36, *p* = 0.02, I^2^ = 81%). The combined analysis showed a statistically significant difference favoring acupuncture, with lower DLQI scores [MD = −4.78, 95% CI (−8.46, −1.11), *p* = 0.01]. Acupuncture vs. cetirizine: Only one study (8) was included in this subgroup. A qualitative analysis was performed, which showed a statistically significant difference favoring acupuncture [MD = −1.69, 95% CI (−2.84, −0.54), *p* < 0.001]. Acupuncture vs. sham acupuncture: This subgroup included three studies (25, 26, 28). Significant heterogeneity was observed (chi^2^ = 14.14, *p* < 0.001, I^2^ = 86%). The combined results revealed a statistically significant difference favoring acupuncture [MD = −3.78, 95% CI (−5.19, −2.36), *p* = 0.03] (Figure 12). These findings suggest that acupuncture significantly improves the quality of life in patients with CSU compared with loratadine, cetirizine, and sham acupuncture though heterogeneity remains high in some subgroups.

#### 3.3.5 Hamilton Depression Scale score

A total of five studies (8, 14, 25, 26, 28) involving 388 patients reported Hamilton Depression Scale (HAMD) scores in patients with CSU. The combined results showed a statistically significant improvement in HAMD scores in patients treated with acupuncture compared with those treated with loratadine, cetirizine, and sham acupuncture, indicating that acupuncture more effectively reduced depression symptoms in CSU patients (refer to Supplementary material 3 for DLQI funnel plot 1 and Egger’s test 1.2 and Supplementary material 4).

#### 3.3.6 Chronic Urticaria Quality of Life Questionnaire

**Figure 13:**
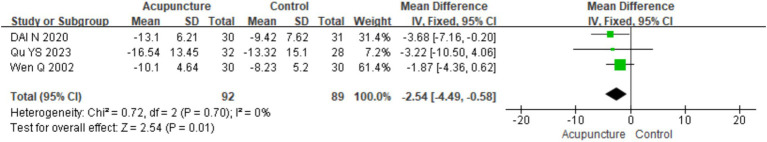
Forest plot of CU-Q2oL.

A total of three RCTs (11, 14, 23) involving 388 patients were included. Heterogeneity testing of the three studies showed no heterogeneity (chi^2^ = 0.72, *p* = 0.70, I^2^ = 0%). A fixed-effects model was used for meta-analysis. The combined results of meta-analysis indicated a statistically significant difference favoring acupuncture over loratadine in improving the Chronic Urticaria Quality of Life Questionnaire (CU-Q2oL) score in patients with CSU [MD = −2.54, 95% CI (−4.49,-0.58), *p* = 0.01] (Figure 13). The funnel plot showed symmetry on both sides, and Egger’s test for publication bias indicated no significant bias (t = −0.68, *p* = 0.618). Due to the limited number of studies (only three), funnel plot analysis, Egger’s test, and sensitivity analysis were not performed.

#### 3.3.7 Number of urticaria wheals

**Figure 14:**
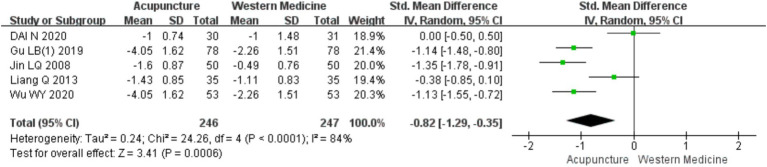
Forest plot of the number of urticaria lesions.

A total of five RCTs (10–12, 16, 19) involving 483 patients were included in the analysis of wheal numbers. Heterogeneity testing showed significant heterogeneity (chi^2^ = 24.26, *p* < 0.0001, I^2^ = 84%). Therefore, a random-effects model was used for meta-analysis. The combined results indicated a statistically significant difference favoring acupuncture over loratadine in reducing the number of wheals in CSU [SMD = −0.82, 95% CI (−1.29, −0.35), *p* < 0.05] (Figure 14). A funnel plot assessment showed symmetry on both sides, and Egger’s test for publication bias indicated no significant bias (t = −0.16, *p* = 0.885) (refer to Supplementary material 3 for the funnel plot and Egger’s test results related to the number of wheals, funnel plot 1 and Egger’s test 1.2).

#### 3.3.8 Size of urticaria wheals

A total of three RCTs (12, 16, 19) involving 276 patients were included. Heterogeneity testing was conducted, which showed that acupuncture had a greater advantage over cetirizine in reducing wheal size in CSU (refer to Supplementary materials 3, 4).

#### 3.3.9 Itch severity

**Figure 15:**
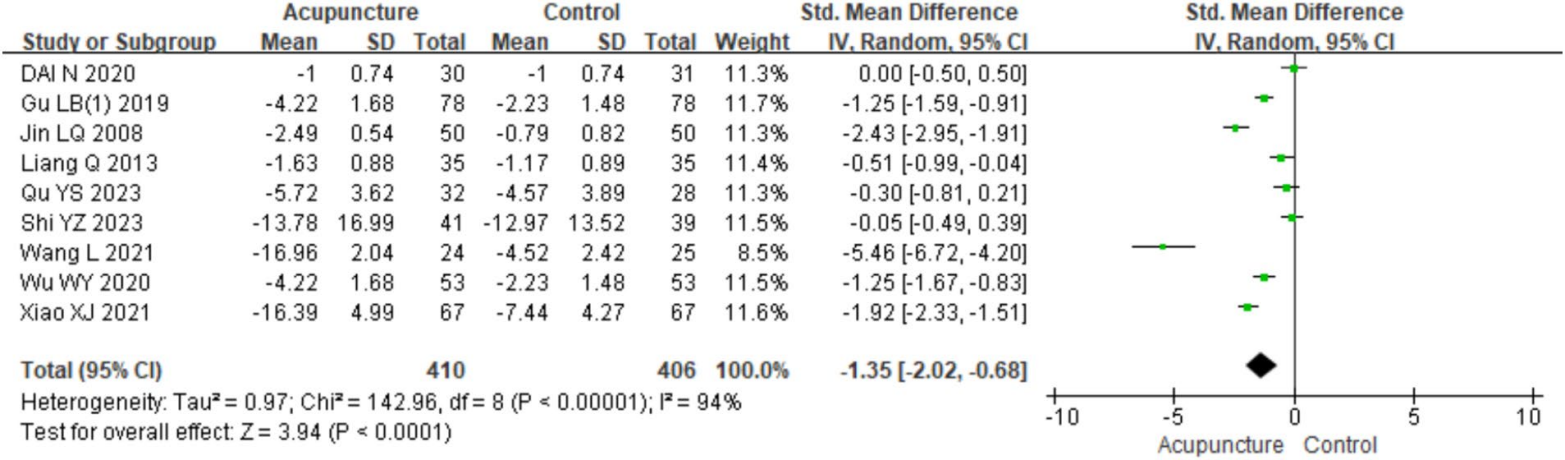
Forest plot of degree of itchiness.

A total of nine RCTs (10–12, 16, 19, 23, 25, 26, 28) involving 816 patients were included. Heterogeneity testing of these studies showed significant heterogeneity (chi^2^ = 142.96, *p* < 0.001, I^2^ = 94%), so a random-effects model was used for meta-analysis. In assessing itch severity, seven studies (10, 11, 19, 23, 25, 26, 28) used the visual analog scale (VAS), whereas two studies (12, 16) used the numeric rating scale (NRS). Therefore, SMD was used for pooling. The combined results indicated a statistically significant difference favoring acupuncture over loratadine and sham acupuncture in improving itch severity in CSU patients [SMD = −1.35, 95% CI (−2.02, −0.68), *p* < 0.001] (Figure 15). The funnel plot showed symmetry on both sides, and Egger’s test for publication bias indicated no significant bias (t = −0.156, *p* = 0.163) (refer to Supplementary material 3 for the funnel plot and Egger’s test results related to itch severity, funnel plot 1 and Egger’s test 1.2).

**Figure 16:**
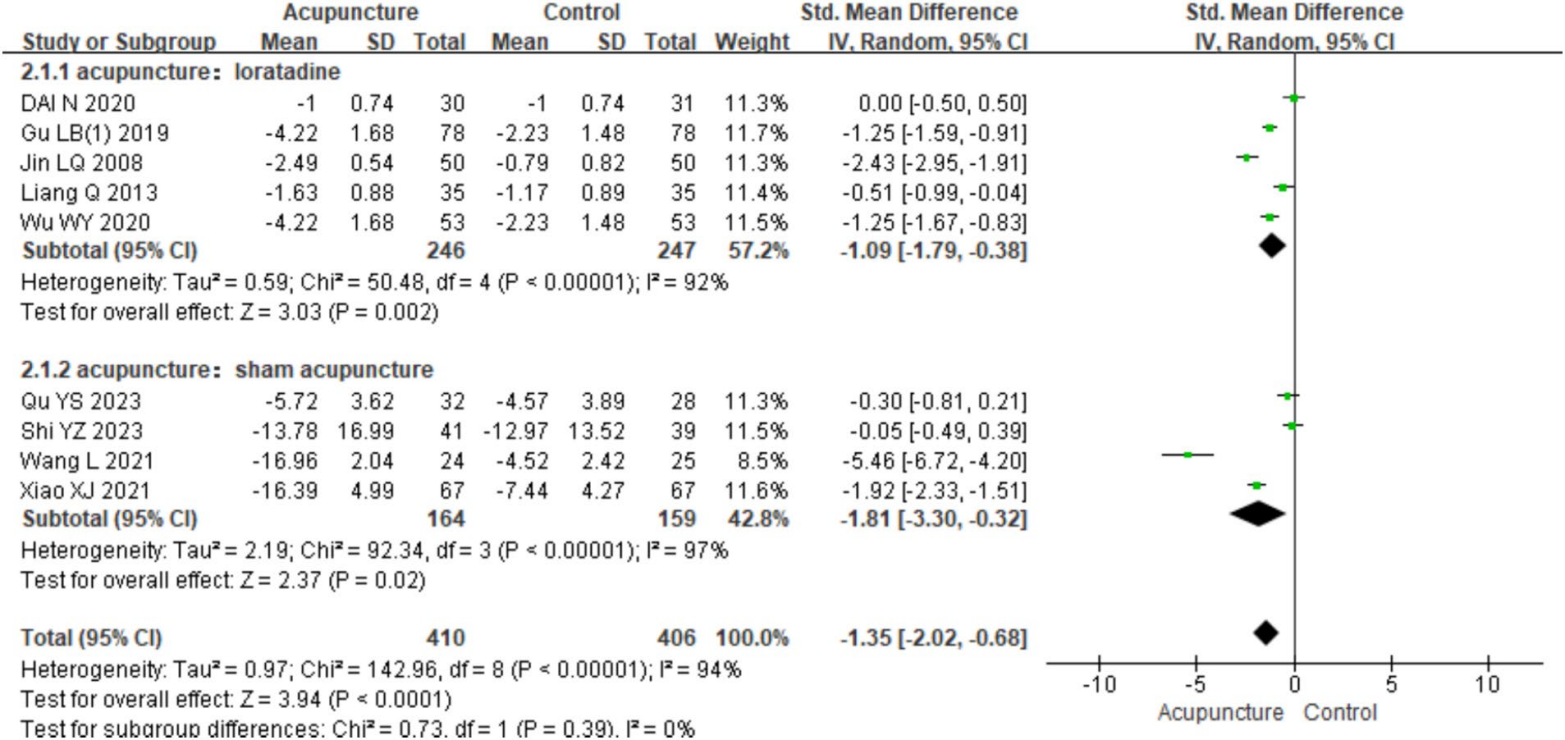
Forest plot of subgroup analyses of degree of itchiness of different intervention methods.

In the subgroup analysis based on different control interventions, the studies were categorized into two subgroups: acupuncture vs. loratadine and acupuncture vs. sham acupuncture. Acupuncture vs. loratadine: This subgroup included five studies (7–10, 31). Significant heterogeneity was observed (chi^2^ = 50.48, *p* < 0.001, I^2^ = 92%). The combined results indicated a statistically significant difference favoring acupuncture [SMD = −1.09, 95% CI (−1.79, −0.38), *p* = 0.002], demonstrating that acupuncture was more effective than loratadine in reducing urticaria wheals. Acupuncture vs. sham acupuncture: This subgroup included four studies (2, 3, 6, 11). Similarly, significant heterogeneity was observed (chi^2^ = 92.34, *p* < 0.001, I^2^ = 97%). The combined results also showed a statistically significant difference favoring acupuncture [SMD = −1.81,95% CI (−3.30, −0.32), *p* = 0.02], indicating that acupuncture was more effective than sham acupuncture in reducing wheal size (Figure 16). Despite the subgroup analysis, significant heterogeneity persisted in both subgroups. This suggests that other factors may contribute to the heterogeneity, which warrants further investigation.

#### 3.3.10 Duration of urticaria flare-ups

**Figure 17:**
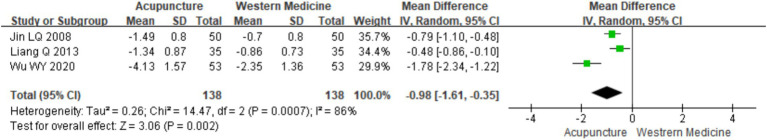
Forest plot of seizure duration.

A total of three RCTs (12, 16, 19) involving 276 patients were included in the analysis of flare-up duration. Heterogeneity testing indicated significant heterogeneity (chi^2^ = 14.47, p < 0.001, I^2^ = 86%), and thus, a random-effects model was applied for meta-analysis. The combined results showed a statistically significant difference favoring acupuncture over loratadine in reducing the duration of CSU flare-ups [MD = −0.98, 95% CI (−1.61, −0.35), *p* = 0.002] (Figure 17). Due to the limited number of included studies (only three), a funnel plot and Egger’s test for publication bias were not performed.

#### 3.3.11 Serum IgE levels

**Figure 18:**
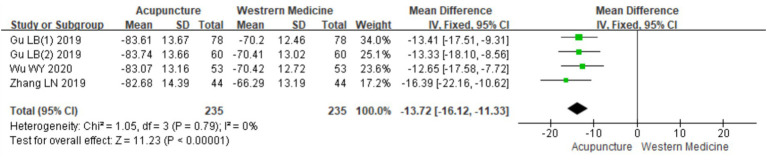
Forest plot of IgE levels.

A total of four RCTs (10, 18–20) involving 470 patients were included in the analysis of serum IgE levels. Heterogeneity testing showed no significant heterogeneity (chi^2^ = 1.05, *p* = 0.79, I^2^ = 0%), indicating consistency across the studies. A fixed-effects model was therefore used for meta-analysis. The combined results showed a statistically significant reduction in serum IgE levels favoring acupuncture over loratadine [MD = −13.72, 95% CI (−16.12, −11.33), *p* < 0.001], indicating that acupuncture was more effective in reducing serum IgE levels in patients with CSU (Figure 18). Due to the limited number of included studies (only four), a funnel plot and Egger’s test for publication bias were not conducted.

#### 3.3.12 IFN-*γ* levels

**Figure 19:**
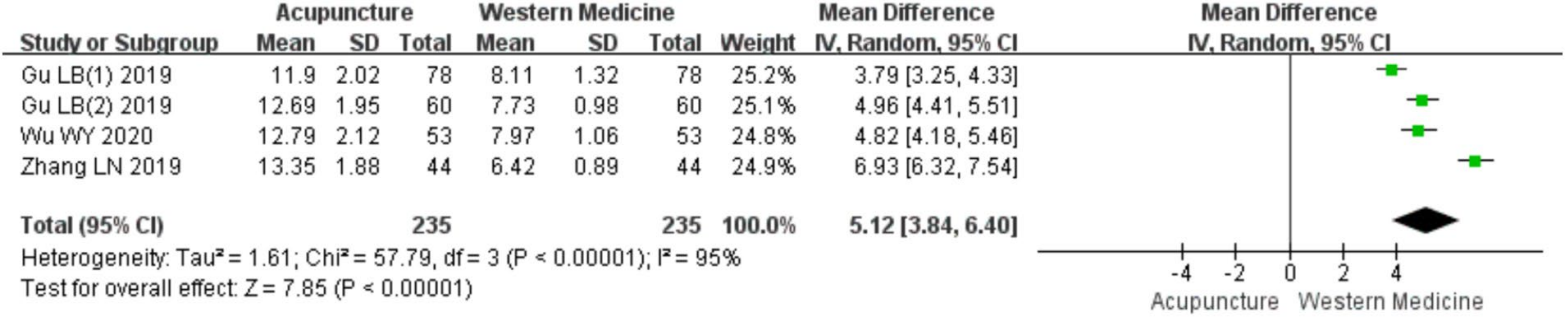
Forest plot of IFN-*γ* levels.

A total of four RCTs (10, 18–20) involving 470 patients were included in the analysis of IFN-γ levels. Heterogeneity testing revealed significant heterogeneity (chi^2^ = 57.79, *p* < 0.001, I^2^ = 95%), so a random-effects model was used for meta-analysis. The combined results showed a statistically significant increase in IFN-γ levels favoring acupuncture over loratadine [MD = 5.12, 95% CI (3.84, 6.40), *p* < 0.001] (Figure 19), indicating that acupuncture was more effective in increasing IFN-γ levels in patients with CSU. Due to the limited number of included studies (only four), a funnel plot and Egger’s test for publication bias were not conducted.

#### 3.3.13 IL-4 levels

**Figure 20:**
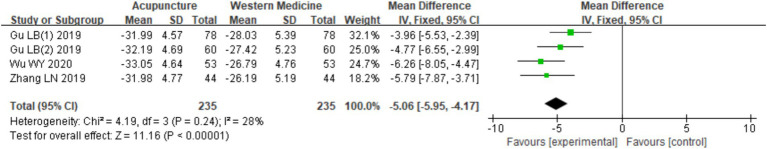
Forest plot of IL-4 levels.

A total of four RCTs (10, 18–20) involving 470 patients were included in the analysis of IL-4 levels. Heterogeneity testing showed low heterogeneity (chi^2^ = 4.19, *p* = 0.24, I^2^ = 28%). A fixed-effects model was therefore used for meta-analysis. The combined results indicated a statistically significant reduction in IL-4 levels favoring acupuncture over loratadine [MD = −5.06, 95% CI (−5.95, −4.17), *p* < 0.001], indicating that acupuncture was more effective in reducing IL-4 levels in patients with CSU (Figure 20). Due to the limited number of studies (only four), a funnel plot and Egger’s test for publication bias were not conducted.

#### 3.3.14 TCM syndrome score

**Figure 21:**
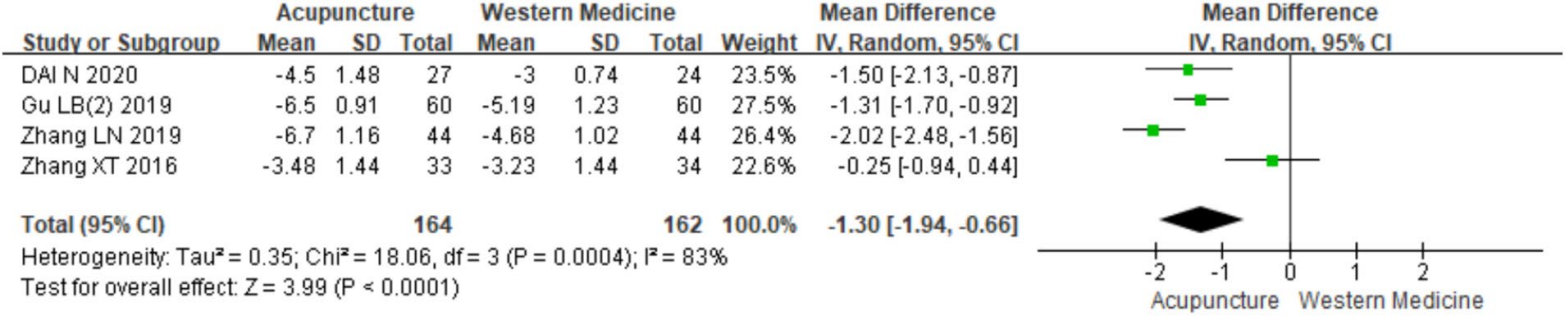
Forest plot of TCM score.

A total of four RCTs (9, 11, 18, 20) involving 326 patients were included in the analysis of TCM syndrome scores. Heterogeneity testing revealed significant heterogeneity (chi^2^ = 18.06, *p* < 0.001, I^2^ = 83%), so a random-effects model was applied for meta-analysis. Since all four studies used the “Guidelines for Clinical Research of New Chinese Medicine” (14) for assessment, MD was used in the meta-analysis. The results showed a statistically significant difference favoring acupuncture over loratadine in improving TCM syndrome scores [MD = −1.30, 95% CI (−1.94, −0.66), *p* < 0.001] (Figure 21), indicating that acupuncture was more effective in improving TCM syndrome scores in patients with CSU. Due to the limited number of studies (only four), a funnel plot and Egger’s test for publication bias were not conducted.

### 3.4 Safety indicators

**Figure 22:**
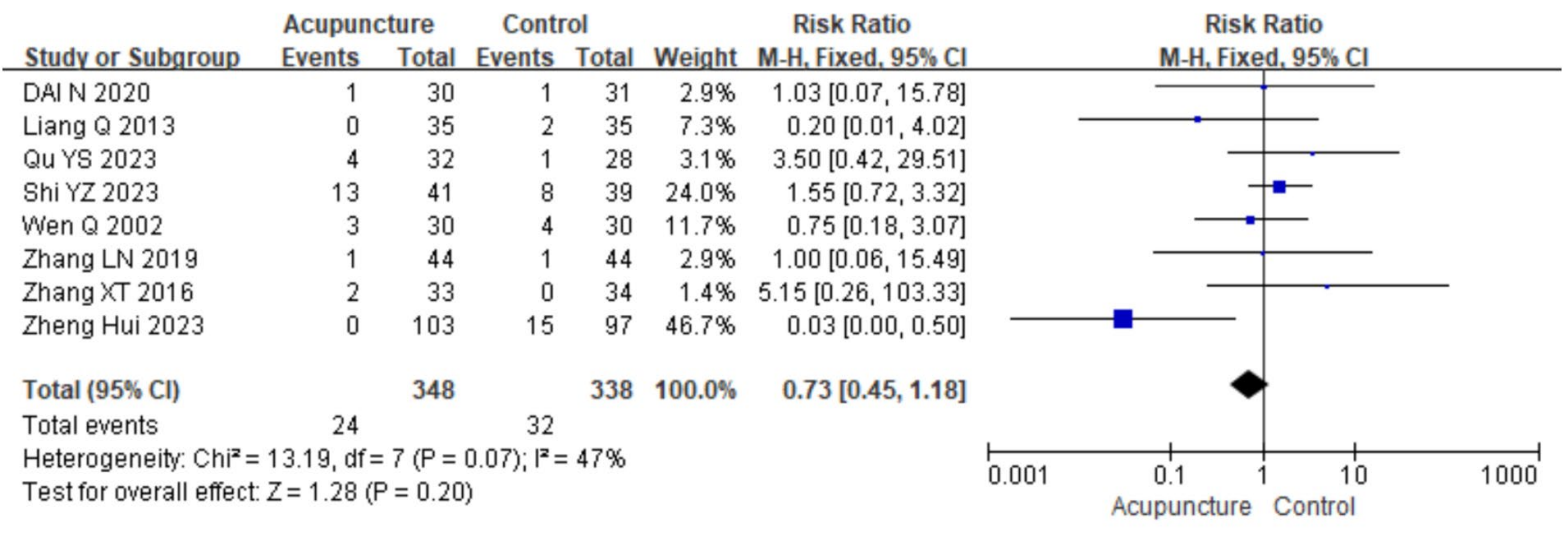
Forest plot of security indicators.

A total of 10 studies (9, 11, 14, 16, 18, 21, 23, 26–28) evaluated the safety of acupuncture, with all reporting no serious adverse events related to acupuncture. Eight studies (9, 11, 14, 16, 18, 23, 27, 28) involving 686 participants reported mild adverse events, primarily bruising, bleeding, and discomfort related to the “Deqi” sensation, such as distension and numbness. A meta-analysis was conducted on these eight studies. Heterogeneity testing showed low heterogeneity (chi^2^ = 13.19, *p* = 0.07, I^2^ = 47%), so a fixed-effects model was used in the meta-analysis. The results showed no statistically significant difference in the occurrences of adverse events between the acupuncture group and control group [RR = 0.73, 95% CI (0.45, 1.18), *p* = 0.20], indicating that acupuncture does not increase the risk of adverse events in the treatment for CSU (Figure 22). A funnel plot assessment for publication bias showed symmetry, and Egger’s test indicated no significant publication bias (t = −0.156, *p* = 0.163) (refer to Supplementary material 3 for the funnel plot and Egger’s test results).

**Figure 23:**
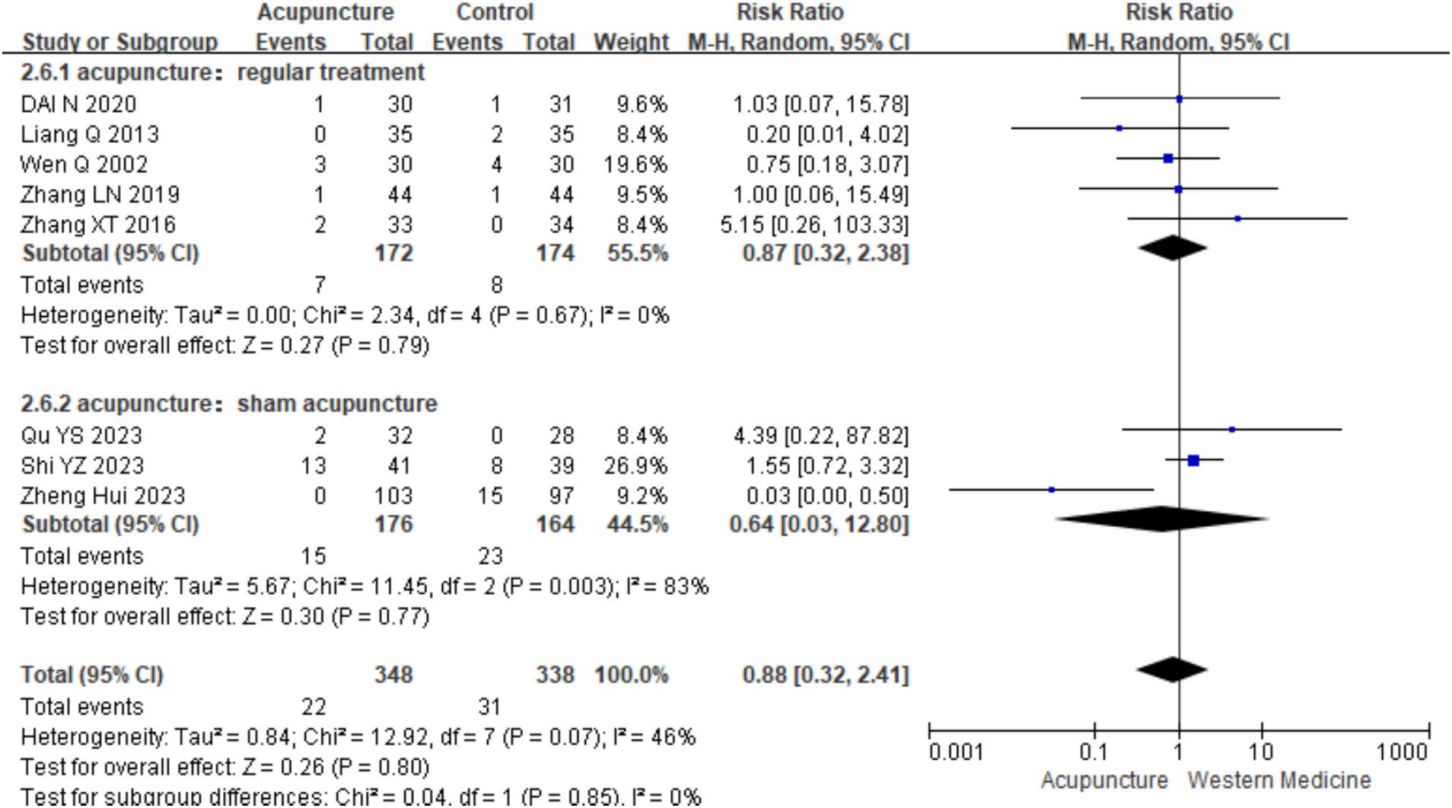
Forest plot of subgroup analyses of security indicators of different intervention methods.

In the subgroup analysis of adverse events based on different control interventions, the studies were categorized into two subgroups: acupuncture vs. loratadine and acupuncture vs. sham acupuncture. Acupuncture vs. loratadine: five studies (9, 11, 14, 16, 18) were included. The combined analysis showed no statistically significant difference in the occurrence of adverse events between acupuncture and loratadine (*p* = 0.79). Acupuncture vs. sham acupuncture: Three studies (23, 27, 28) were included. The combined analysis also showed no statistically significant difference in adverse events between acupuncture and sham acupuncture (*p* = 0.77). This suggests that acupuncture does not increase the risk of adverse events compared with loratadine or sham acupuncture in the treatment for CSU. Regarding publication bias, the funnel plot appeared symmetrical, and Egger’s test further indicated no significant publication bias (*t* = −0.96, *p* = 0.375) (Figure 23). However, there was still significant heterogeneity in the acupuncture vs. sham acupuncture subgroup, which may be attributable to other factors (refer to Supplementary material 3 for the funnel plot related to adverse events, Funnel plot 2).

### 3.5 Sensitivity analysis

A sensitivity analysis was conducted using the stepwise exclusion method for different intervention outcome indicators, including efficacy rate, recurrence rate, UAS7, number of wheals, itch severity, DLQI, and HAMD. The results indicated that excluding any individual study had little effect on the effect size of meta-analysis results. This suggests low sensitivity, indicating that the conclusions are stable and reliable (refer to Supplementary material 3 for the sensitivity analysis table).

### 3.6 Quality of evidence assessment

The quality of the evidence was assessed considering five downgrading factors: risk of bias, inconsistency, indirectness, imprecision, and publication bias. Among the 22 studies, four used random envelope allocation, eight used random number tables to generate random sequences, nine only mentioned randomization without specifying the method, and one did not mention randomization. Only five studies mentioned blinding of assessors, which lowered the risk of bias by one level. The funnel plot and statistical tests for the outcome measures showed no publication bias, so no downgrading occurred. Of the 14 outcome measures, only five had I^2^ < 50%, whereas the remainder had high heterogeneity, leading to a downgrade of one level for inconsistency. Regarding safety outcomes, ten studies reported on safety, with eight reporting mild adverse events but no serious adverse events, so no downgrade was applied for publication bias. In summary, the quality of evidence for efficacy rate, recurrence rate, CU-Q2oL, duration of flare-ups, serum IgE levels, and IL-4 levels was rated as moderate. The quality of evidence for UAS7, number of wheals, wheal size, itch severity, DLQI, HAMD, IFN-*γ* levels, and TCM syndrome scores was rated as low. Detailed results are presented in Table 4.

**Table 4:** Overall evidence for GRADE quality rating of seven included studies.

Outcomes	Number of studies	Study design	Sample size TC	Effect size	Risk of Bias	Inconsistency	Indirect	Imprecision	Publication Bias	Evidence Grade
Efficacy rate	17	RCT	T 681 C 663	1.19,(1.12,1.25)	−1a					Middle
Recurrence rate	5	RCT	T 135 C 112	0.34,(0.21,0.55)	−1a					Middle
UAS		RCT	T 380 C 348	−0.84,(−1.43,-0.25)	−1a	−1b				Low
DLQI	6	RCT	T 254 C 250	−0.85,(−1.35.-0.35)	−1a	−1b				Low
HAMD	5	RCT	T 196 C 192	−1.72, (−2.77, −0.68)	−1a	−1b				Low
CU-Q2oL	3	RCT	T 92 C 89	−2.54, (−4.49, −0.58)	−1a					Middle
Number of wheals	5	RCT	T 246 C 247	−0.82, (−1.29, −0.35)	−1a	−1b				Low
Size of wheals	3	RCT	T 138 C 138	−0.75, (−1.35, −0.14)	−1a	−1b				Low
Itching severity	9	RCT	T 410 C 406	−1.35, (−2.02, −0.68)	−1a	−1b				Low
Seizure duration	3	RCT	T 138 C 138	−0.94, (−1.28, −0.60)	−1a					Middle
IgE	4	RCT	T 235 C 235	−13.72, (−16.12, −11.33)	−1a					Middle
IFN-γ	4	RCT	T 235 C 236	5.12, (3.84, 6.40)	−1a	−1b				Low
IL-4	4	RCT	T 235 C 237	−5.10, (−6.16, −4.05)	−1a					Middle
TCM syndrome score	4	RCT	T 164 C 162	−1.11, (−1.78, −0.43)	−1a	−1b				Low

T, treatment; C, control; RCT, randomized controlled trial; RR, risk ratio; CI, confidence interval. a The design of the trial has a large bias in randomization, allocation concealment, or blinding. b The credible interval overlaps less, the *P*-value of the heterogeneity test is small, and the I of the combined results is large.

## 4 Discussion and conclusion

### 4.1 Mechanisms of acupuncture in the treatment for chronic spontaneous urticaria

Urticaria is a common dermatological condition that tends to recur, causing significant disruptions in patients’ daily lives (3). The exact pathogenesis of urticaria remains unclear, but it is generally believed to involve the activation and degranulation of MCs through both immune and non-immune pathways, leading to the release of histamine, prostaglandins, leukotrienes, and cytokines, which contribute to the development of CSU (32). Recent research has delved into the mechanisms by which acupuncture treats CSU and found that acupuncture can modulate humoral immunity and cellular immunity and inhibit MC degranulation through multiple pathways, including the regulation of central nervous system pathways.

#### 4.1.1 Acupuncture modulates humoral immunity and reduces inflammatory mediators

##### 4.1.1.1 Reduction in serum IgE levels

While a subset of CSU patients showed higher serum IgE levels compared with healthy individuals, existing evidence regarding its universal correlation with disease severity remains inconclusive (33). IgE, produced by allergen-specific B cells, binds to high-affinity receptors on MCs and basophils. Upon re-exposure to inflammatory triggers, IgE binds to receptors on sensitized cells, activating MCs and basophils and contributing to CSU pathogenesis (34). Clinical studies (35) indicate that acupuncture reduces serum IgE levels in CSU patients. This reduction may involve the suppression of IgE production (e.g., via downregulating Th2 cytokines such as IL-4/IL-13) and/or enhanced IFN-*γ* activity, which antagonizes IgE synthesis. Acupuncture simultaneously reduces inflammatory mediator release and stabilizes target cell membranes, synergistically regulating humoral immunity (35).

##### 4.1.1.2 Regulation of cytokine and chemokine levels

In addition to the central role of MCs in CSU pathogenesis, various cytokines such as interleukins and transforming growth factor (TGF) are involved in the immune-inflammatory response (36–38). MC-derived mediators, including histamine, tryptase, serotonin (5-HT), prostaglandins, and leukotrienes, along with platelet activation, synergistically exacerbate allergic reactions. An over-release of these components leads to clinical manifestations such as severe itching, wheals, edema, and erythema. By suppressing the release of histamine and other MC-derived mediators (e.g., proteases and prostaglandins) that directly drive vascular hyperpermeability and sensory nerve activation, acupuncture mitigates localized inflammatory responses, thereby alleviating hallmark urticaria symptoms, including pruritus, wheal formation, and angioedema. Animal studies (39) have shown that by inhibiting the release of histamine, 5-HT, TNF-*α*, and other inflammatory mediators, acupuncture can significantly reduce inflammation and improve urticaria symptoms. In addition, acupuncture decreases IL-33 and ST2 expression at urticarial lesion sites (40), which further confirms its role in modulating humoral immunity.

#### 4.1.2 Acupuncture regulates cellular immunity

##### 4.1.2.1 Increase in CD4+/CD8 + ratio

CD4 + and CD8 + cells are critical T lymphocyte subsets in cellular immunity. Studies (29) have shown that the balance of the CD4+/CD8 + ratio reflects immune homeostasis, and its disruption is a key factor in urticaria. Emerging evidence suggests acupuncture may ameliorate CSU through multitarget immunomodulation. Preliminary studies indicate potential regulatory effects on T-cell homeostasis (e.g., helper T cell (Th)1/Th2 rebalancing and regulatory T cell (Treg) induction), coupled with the suppression of MC degranulation via STAT3-dependent pathways. These coordinated actions are correlated with clinical improvements, as evidenced by low UAS7 scores and decreased serum levels of histamine, IL-6, and other effector molecules in responders (41, 42).

##### 4.1.2.2 Th1/Th2 balance

CD4 + T lymphocytes can differentiate into helper T (Th) cells under environmental stimuli, with Th1 and Th2 being two subgroups. Th1 secretes IL-12 and IFN-*γ*, mediating cellular immunity, whereas Th2 primarily secretes IL-4, IL-5, and IL-13, mediating humoral immunity and allergic responses (43). IL-10 is primarily produced by Treg, whereas IL-8 is predominantly secreted by neutrophils and epithelial cells during inflammatory responses. A disrupted Th1/Th2 balance can lead to urticaria (43). Acupuncture has been shown to restore this balance by reducing Th2 cytokine secretion and modulating Treg/Th17 homeostasis, alleviating CSU symptoms.

##### 4.1.2.3 Th17/Treg balance

Th17 and Treg maintain immune balance, and an increased Th17/Treg ratio may induce IgE production, triggering urticaria (44, 45). Acupuncture can regulate this balance by increasing the expression of TGF-*β* and forkhead box P3 (Foxp3), reducing IL-17 expression (46).

#### 4.1.3 Acupuncture inhibits mast cell degranulation via multiple pathways

##### 4.1.3.1 Downregulation of the Lyn-Syk/MAPK/NF-KB signaling pathway

Lyn and Syk tyrosine kinases are key proteins in the activation of MCs through the FcεRI receptor. Inhibition of these proteins can reduce MC activation and allergic reactions (47, 48). Studies also reported that acupuncture at specific points reduces serum p-Lyn and p-Syk concentrations, alleviating edema in “allergen-induced chronic urticaria rat models” (it is worth noting that current animal models rely on pharmacologically induced approaches as spontaneous CSU models are unavailable). Animal studies (39) further demonstrated that acupuncture reduces the expression of phosphorylated Lyn and Syk kinases, which are critical components of FcεRI-mediated MC activation. This suppression is correlated with decreased histamine and 5-HT levels, linking pathway inhibition to clinical symptom relief.

##### 4.1.3.2 Inhibition of Ca^2+^ influx

Ca^2+^ influx plays a crucial role in MC degranulation (49). Acupuncture regulates the PIP2/IP3/Ca^2+^ signaling pathway, inhibiting MC degranulation and inflammation (50).

##### 4.1.3.3 Inhibition of the JAK1/STA1, PI3K/AKT signaling pathway

The PI3K/AKT pathway mediates cytokine and growth factor responses and is involved in MC maturation (51). Acupuncture has been shown to regulate this pathway, inhibiting MC degranulation (52–54).

##### 4.1.3.4 Acupuncture regulates the central nervous system

Inflammatory mediators released by immune cells act on neurons, releasing neuropeptides such as substance P and CGRP, leading to neurogenic inflammation and itching (55, 56). Acupuncture can modulate the nervous system, reducing pruritus and improving urticaria symptoms (57, 58).

### 4.2 Analysis of study results

This meta-analysis included 22 RCTs involving 1,867 patients. Acupuncture showed a higher efficacy than medication (loratadine, cetirizine, cimetidine, and chlorpheniramine) and sham acupuncture in reducing UAS7 scores, recurrence rates, wheal size and number, itching and symptom duration; improving quality of life scores (DLQI, HAMD, and CU-Q2oL); and regulating serum IgE, IFN-*γ* and IL-4 levels and TCM syndrome scores. No serious adverse events were reported. Despite these promising results, the included RCTs showed moderate to high risk of bias and heterogeneity, likely due to differences in patient characteristics, disease severity, acupuncture points, and treatment duration. Based on GRADE assessments, the evidence supports a low to moderate recommendation for acupuncture in treating CSU although the results remain positive and effective.

Antihistamine-refractory CSU refers to urticaria that remains unresponsive to standard or double-dose antihistamine treatment for 2–4 weeks, with a disease duration of ≥6 weeks, and is associated with non-histamine pathways or autoimmune mechanisms. Available targeted therapies for this condition include Bruton tyrosine kinase inhibitors (e.g., remibrutinib and rilzabrutinib), anti-KIT (barzolvolimab and briquilimab), anti-IL-4Rα (dupilumab), anti-thymic stromal lymphopoietin (tezepelumab), and MRGPRX2 antagonists, to name a few (59, 60). Although these targeted drugs show significant efficacy, they carry risks such as infection, bleeding, and abnormal liver function and are expensive.

Acupuncture, on the other hand, can modulate the immune system through multiple mechanisms, offering better safety at lower costs, which makes it suitable for patients requiring long-term management or those who are intolerant to medications. Future clinical studies should explore the use of acupuncture in treating patients with antihistamine-refractory CSU.

### 4.3 Limitations

This study has several limitations. First, the search was limited to Chinese and English literature, which may have resulted in missing relevant studies. Second, the outcome measures varied across studies, with some indicators supported by only a few studies, which reduced the robustness of the evidence. Third, some studies had small sample sizes, lacked proper randomization and blinding, and did not register clinical protocols, which affected the overall quality. Finally, variations in acupuncture points, frequency (ranging from twice weekly to daily sessions), and treatment duration (from 2 to 12 weeks) across studies contributed to heterogeneity. For instance, some studies focused on traditional acupoints like SP10 (Xuehai) and LI11 (Quchi), whereas others incorporated additional points based on syndrome differentiation. This variability reflects diverse theoretical frameworks and clinical practices, which limits the generalizability of our findings and makes it challenging to establish standardized protocols for CSU. Future studies should aim to standardize acupuncture regimens and conduct subgroup analyses to explore the effects of frequency, duration, and techniques on efficacy and safety.

## 5 Conclusion

In conclusion, this study provides evidence that acupuncture can be an effective treatment for CSU and can improve various clinical outcomes with high safety. However, due to the limitations and heterogeneity observed, future studies should focus on larger, multicenter trials with low risk of bias, standardized acupuncture protocols, and robust methodology to validate these findings. Researchers should also prioritize objective, reproducible outcome measures for inclusion in future meta-analyses to better clarify the relationship between acupuncture and CSU.

## Data Availability

The original contributions presented in the study are included in the article/Supplementary material; further inquiries can be directed to the corresponding author.

## References

[1] Zuberbier T Abdul Latiff AH Abuzakouk M Aquilina S Asero R Baker D . The international Eaaci/Ga^2^Len/EuroGuiDerm/Apaaaci guideline for the definition, classification, diagnosis, and management of urticaria. Allergy. (2022) 77:734–66. doi: 10.1111/all.15090, PMID: 34536239

[2] Li J Mao D Liu S Liu P Tian J Xue C . Epidemiology of urticaria in China: a population-based study. Chin Med J. (2022) 135:1369–75. doi: 10.1097/Cm9.0000000000002172, PMID: 35830258 PMC9433071

[3] Gonçalo M Gimenéz-Arnau A Al-Ahmad M Ben-Shoshan M Bernstein JA Ensina L . The global burden of chronic urticaria for the patient and society. Br J Dermatol. (2021) 184:226–36. doi: 10.1111/bjd.19561, PMID: 32956489

[4] Chaichan W Ruengorn C Thavorn K Hutton B Szepietowski JC Bernstein JA . Comparative safety profiles of individual second-generation H1-antihistamines for the treatment of chronic Urticaria: a systematic review and network Meta-analysis of randomized controlled trials. J Allergy Clin Immunol Pract. (2023) 11:2365–81. doi: 10.1016/j.jaip.2023.03.058, PMID: 37088368

[5] Tang Y Cheng S Wang J Jin Y Yang H Lin Q . Acupuncture for the treatment of itch: peripheral and central mechanisms. Front Neurosci. (2021) 15:786892. doi: 10.3389/fnins.2021.786892, PMID: 35431769 PMC9005788

[6] Shengyuan Q Deqiang G Chenchen X Bingyuan C. Advances in the mechanism of acupuncture in the treatment of chronic Urticaria. Global Chinese Med. (2024) 17:947–52. doi: 10.3969/j.issn.1674-1749.2024.05.037

[7] Liyi C Yuanqi G. Observations on the short-term efficacy of Bo’s abdominal acupuncture for the treatment of chronic urticaria. Chin Acup Moxibustion. (2005) 25:6. doi: 10.3321/j.issn:0255-2930.2005.11.006

[8] Zhongxun W Lin G Xinyi S Xianming L. Treatment of chronic urticaria with the regulating Spirit acupuncture method: 34 cases. Zhejiang. Clin Med. (2021) 23:197–201. doi: 10.3969/j.issn.1008-7664.2021.02.016

[9] Xiaoting Z. Clinical observation of the Dong's wooden points treatment for chronic urticaria of blood deficiency and wind dryness type. Guangzhou Univ Trad Chin Med. (2016).

[10] Gu Libin W Wenyin YL Shuqing Z Xiaofeng K. Treatment of 78 cases of refractory chronic urticaria with the Yin-Yang hidden method on shoulder and Yangxi points. Sichuan. Chin Med. (2019) 37:191–193.

[11] Na Dai. Clinical study on the root-cutting method in treating chronic urticaria of blood deficiency and wind dryness type. Chengdu Univ Trad Chin Med. (2020). doi: 10.26988/d.cnki.gcdzu.2020.000401

[12] Lingqing J Boxu L. Observational study on the efficacy of four-position navel acupuncture combined with abdominal acupuncture in 50 cases of chronic urticaria. Zhejiang. J Chin Med. (2018) 53:29. doi: 10.13633/j.cnki.zjtcm.2018.04.029

[13] Chunhua S Guirong D Suqing Y Lin M Yingqi L Yueze X . Clinical observation on the treatment of chronic urticaria with acupuncture at Quchi point. Shanghai J Acup Moxibustion. (2005) 8:17–8. doi: 10.13460/j.issn.1005-0957.2005.08.009

[14] Qin W. Clinical observation of Tongyuan acupuncture for chronic urticaria. Guangzhou Univ Trad Chin Med. (2022). doi: 10.27044/d.cnki.ggzzu.2022.000372

[15] Jieru H Jinfeng D. Treatment of 30 cases of chronic urticaria with surrounding acupuncture. J Anhui Univ Trad Chin Med. (2009) 28:51–2. doi: 10.3969/j.issn.1000-2219.2009.04.024

[16] Liang J. Clinical comparative study of the "Qingxue Antiallergy" acupuncture method in treating chronic urticaria. Guangzhou University of Traditional Chinese Medicine (2013).

[17] Xiaowei Chen. Clinical study on the "four Acupoints for Urticaria" acupuncture method in treating chronic urticaria. Guangzhou Univ Trad Chin Med. (2009).

[18] Zhang Liangnan G Libin YL. The efficacy of the "six points for wind treatment" acupuncture method in chronic urticaria and its effects on serum immune-inflammatory factors. Clin Acup J. (2019) 35:29–32. doi: 10.3969/j.issn.1005-0779.2019.11.009

[19] Wu Wenyin G Libin YL. Observation on the efficacy of the "six points for wind treatment" acupuncture method in chronic urticaria. Shanghai J Acup Moxibustion. (2020) 39:551–4. doi: 10.13460/j.issn.1005-0957.2020.05.0551

[20] Gu Libin W Wenyin YL Shuqing Z Xiaofeng K. Clinical observation of the "three points of the divine Acupoints" acupuncture method in treating chronic urticaria. Shanghai J Acup Moxibustion. (2019) 38:1136–9. doi: 10.13460/j.issn.1005-0957.2019.10.1136

[21] Yuming W Bingnan C Ping S Dong S. Clinical observation of treating chronic spontaneous urticaria with the theory of acupuncture based on the heart. Chin Clin Doc J. (2018) 46:110–2. doi: 10.3969/j.issn.2095-8552.2018.01.041

[22] Chunlei Tang. Clinical observation and preliminary exploration of the mechanism of acupuncture in the treatment of chronic urticaria. Dalian Medical University. (2006).

[23] Shengyuan Qu. Randomized controlled trial of acupuncture for chronic spontaneous urticaria and fmri brain function imaging study. China Academy of Chinese Medical Sciences. (2023).

[24] Zhongzhi Z Ye M. Clinical observation of acupuncture for urticaria. Heilongjiang Med Sci. (2003) 5:51. doi: 10.3969/j.issn.1008-0104.2003.05.042

[25] Wang Lu. Clinical efficacy and quality of life evaluation of acupuncture for chronic urticaria. Hunan Univ Trad Chin Med. (2021). doi: 10.27138/d.cnki.ghuzc.2021.000138

[26] Xianjun X. Randomized controlled trial of acupuncture for chronic spontaneous urticaria. Chengdu Univ of Trad Chin Med. (2021).

[27] Zheng H Xiao XJ Shi YZ Zhang LX Cao W Zheng QH . Efficacy of acupuncture for chronic spontaneous Urticaria: a randomized controlled trial. Ann Intern Med. (2023) 176:1617–24. doi: 10.7326/M23-1043, PMID: 37956431

[28] Shi YZ Yu SG Zheng H Zheng QH Zhou SY Huang Y . Acupuncture for patients with chronic spontaneous Urticaria: a randomized, sham-controlled pilot trial. Chin J Integr Med. (2023) 29:924–31. doi: 10.1007/s11655-023-3741-x, PMID: 37561282

[29] Chunhua S. Clinical comparative study on the function and efficacy of acupuncture at Quchi point for the treatment of chronic Urticaria. Heilongjiang: Heilongjiang University of Chinese Medicine (2009-09-28).

[30] Chinese Medical Association Dermatology and Venereology Branch. Guidelines for the diagnosis and treatment of urticaria (2007) Chin J Dermatol, 2007, (10): 591–593.

[31] Sterne JAC Savović J Page MJ Elbers RG Blencowe NS Boutron I . RoB 2: a revised tool for assessing risk of bias in randomised trials. BMJ. (2019) 366:l4898. doi: 10.1136/bmj.l4898 31462531

[32] Jiayi W Jie L. Research progress on the pathogenesis of chronic urticaria. J Cent South Univ. (2023) 48:1602–10. doi: 10.11817/j.issn.1672-7347.2023.230037, PMID: 38432889 PMC10929888

[33] Worm M Vieths S Mahler V. An update on anaphylaxis and urticaria. J Allergy Clin Immunol. (2022) 150:1265–78. doi: 10.1016/j.jaci.2022.10.014, PMID: 36481047

[34] Kessel A Yaacoby-Bianu K Vadasz Z Peri R Halazs K Toubi E. Elevated serum B-cell activating factor in patients with chronic urticaria. Hum Immunol. (2012) 73:620–2. doi: 10.1016/j.humimm.2012.03.016, PMID: 22504411

[35] Frossi B De Carli S Bossi F Pucillo C De Carli M. Co-occurrence of chronic spontaneous Urticaria with immunoglobulin a deficiency and autoimmune diseases. Int Arch Allergy Immunol. (2016) 169:130–4. doi: 10.1159/000445058, PMID: 27073906

[36] Hori H Fukuchi T Sugawara H. Chronic urticaria with inflammation. Eur J Intern Med. (2021) 83:84–5. doi: 10.1016/j.ejim.2020.11.006, PMID: 33208298

[37] Cugno M Asero R Tedeschi A Lazzari R Marzano A. Inflammation and coagulation in urticaria and angioedema. Curr Vasc Pharmacol. (2012) 10:653–8. doi: 10.2174/157016112801784558 22272913

[38] Varghese R Rajappa M Chandrashekar L Kattimani S Archana M Munisamy M . Association among stress, hypocortisolism, systemic inflammation, and disease severity in chronic urticaria. Ann Allergy Asthma Immunol. (2016) 116:344–8.e1. doi: 10.1016/j.anai.2016.01.016, PMID: 26905640

[39] Yueming W Tieming M. Effects of acupuncture pretreatment on serum IgE and expression of phosphorylated tyrosine protein kinases in the skin of urticaria rats. Acupunct Res. (2020) 45:111–6. doi: 10.13702/j.1000-0607.190188 32144920

[40] Sijia L Juntong L Jiquan L Chengcheng W Miao Y Zhiqiang G . The effects of acupuncture pretreatment at "Quchi" and "Xuehai" on mast cells and interleukin-33, tumor Suppressor-2 in urticaria rats. Acupunct Res. (2023) 48:311–24. doi: 10.13702/j.1000-0607.2021128

[41] Zou Y Xintong L Qingti T. Effects of acupuncture combined with autohemotherapy on chronic urticaria of blood deficiency and wind dryness type and its impact on Uas scores, peripheral blood T lymphocyte Stat3 mrna expression levels. J Chin Med. (2019) 37:1781–4. doi: 10.13193/j.issn.1673-7717.2019.07.061

[42] Johnson DE O'keefe RA Grandis JR. Targeting the Il-6/Jak/Stat3 signalling axis in cancer. Nat Rev Clin Oncol. (2018) 15:234–48. doi: 10.1038/nrclinonc.2018.8, PMID: 29405201 PMC5858971

[43] Giménez-Arnau AM Demontojoye L Asero R Cugno M Kulthanan K . The pathogenesis of chronic spontaneous Urticaria: the role of infiltrating cells. J Allergy Clin Immunol Pract. (2021) 9:2195–208. doi: 10.1016/j.jaip.2021.03.033, PMID: 33823316

[44] Dos Santos JC Azor MH Nojima VY Lourenço FD Prearo E Maruta CW . Increased circulating pro-inflammatory cytokines and imbalanced regulatory T-cell cytokines production in chronic idiopathic urticaria. Int Immunopharmacol. (2008) 8:1433–40. doi: 10.1016/j.intimp.2008.05.016, PMID: 18586117

[45] Fasching P Stradner M Graninger W Dejaco C Fessler J. Therapeutic potential of targeting the Th17/Treg Axis in autoimmune disorders. Molecules. (2017) 22. doi: 10.3390/molecules22010134, PMID: 28098832 PMC6155880

[46] He Qingxuan. Effects of acupuncture on Th17/Treg-related transcription factors in urticaria mice. Liaoning Univ Trad Chin Med. (2020). doi: 10.27213/d.cnki.glnzc.2020.000393

[47] Jie L Hongwen L. Research progress on the immunopathogenesis of chronic urticaria. Clin Dermatology J. (2020) 49:313–6. doi: 10.16761/j.cnki.1000-4963.2020.05.019

[48] Xiaohong Z. Experimental study on the anti-allergic effect of electroacupuncture in urticaria rats via Mapk/Nf-κB signaling pathway regulation of mast cells. Liaoning Univ Trad Chin Med. (2020). doi: 10.27213/d.cnki.glnzc.2020.000023

[49] Ying P Xue Z Zhongzheng L Xue Z. Study on the role of calcium ions and mast cells in acupuncture effects. Clin Exp Med J. (2017) 16:1145–8. doi: 10.3969/j.issn.1671-4695.2017.12.001

[50] Jinzhuo T. Study on the mechanism of acupuncture at Xuehai and Quchi points based on Pip2/Ip3/Ca2+ signaling pathway in the treatment of chronic urticaria. Liaoning Univ Trad Chin Med. (2019). doi: 10.27213/d.cnki.glnzc.2019.000434

[51] Lijuan J Yuguo L Wei C Miao C Wenfeng Z. Pi3K/Akt signaling pathway in the prevention and treatment of type 2 diabetes mellitus with traditional Chinese medicine. Chin Med J. (2021) 36:5405–8.

[52] Nakajima S Ishimaru K Kobayashi A Yu G Nakamura Y Oh-oka K . Resveratrol inhibits Il-33-mediated mast cell activation by targeting the Mk2/3-Pi3K/Akt axis. Sci Rep. (2019) 9:18423. doi: 10.1038/s41598-019-54878-5, PMID: 31804564 PMC6895112

[53] Zhao JW Ping JD Wang YF Liu XN Li N Hu ZL . Vitamin D suppress the production of vascular endothelial growth factor in mast cells by inhibiting the Pi3K/Akt/p38 Mapk/Hif-1α pathway in chronic spontaneous urticaria. Clin Immunol. (2020) 215:108444. doi: 10.1016/j.clim.2020.108444, PMID: 32339669

[54] Baixue L Jiquan L Lie W Yiyan H Sijia L. Study on the effect of Electroacupuncture on mast cell degranulation in Urticaria rats based on the Pi3K/Pdk1/Akt signaling pathway. Chinese J Trad Chinese Med. (2022) 37:2866–70.

[55] Ruppenstein A Limberg MM Loser K Kremer AE Homey B Raap U. Involvement of neuro-immune interactions in pruritus with special focus on receptor expressions. Front Med. (2021) 8:627985. doi: 10.3389/fmed.2021.627985, PMID: 33681256 PMC7930738

[56] Kabata H Artis D. Neuro-immune crosstalk and allergic inflammation. J Clin Invest. (2019) 129:1475–82. doi: 10.1172/Jci124609, PMID: 30829650 PMC6436850

[57] Snyder AZ Raichle ME. A brief history of the resting state: the Washington university perspective. NeuroImage. (2012) 62:902–10. doi: 10.1016/j.neuroimage.2012.01.044, PMID: 22266172 PMC3342417

[58] Wang Y Fang JL Cui B Liu J Song P Lang C . The functional and structural alterations of the striatum in chronic spontaneous urticaria. Sci Rep. (2018) 8:1725. doi: 10.1038/s41598-018-19962-2, PMID: 29379058 PMC5789061

[59] Metz M Sussman G Gagnon R Staubach P Tanus T Yang W . Fenebrutinib in H1 antihistamine-refractory chronic spontaneous urticaria: a randomized phase 2 trial. Nat Med. (2021) 27:1961–9. doi: 10.1038/s41591-021-01537-w, PMID: 34750553 PMC8604722

[60] Kolkhir P Bonnekoh H Metz M Maurer M. Chronic spontaneous Urticaria: a review. JAMA. (2024) 332:1464–77. doi: 10.1001/jama.2024.15568, PMID: 39325444

[61] Xiaoyu Z ed. Guidelines for clinical research of new Chinese medicine. Beijing: China Medical Science Tech Press (2002).

[62] Jianli C. The effect of acupuncture on serum IgE level in patients with chronic urticaria. J Tradit Chin Med. (2006) 26:189–90. PMID: 17078446

[63] Sánchez-Machín I Iglesias-Souto J Franco A Barrios Y Gonzalez R Matheu V. T cell activity in successful treatment of chronic urticaria with omalizumab. Clin Mol Allergy. (2011) 9:11. doi: 10.1186/1476-7961-9-11, PMID: 21791043 PMC3159131

