# Supplementary figures and images for: Clinical efficacy and safety of acupuncture in the treatment for chronic spontaneous urticaria: a systematic review and meta-analysis
Source: Front Med (Lausanne). 2025 May 30;12:1498795. doi: 10.3389/fmed.2025.1498795 (PMC12164643; doi:10.3389/fmed.2025.1498795)

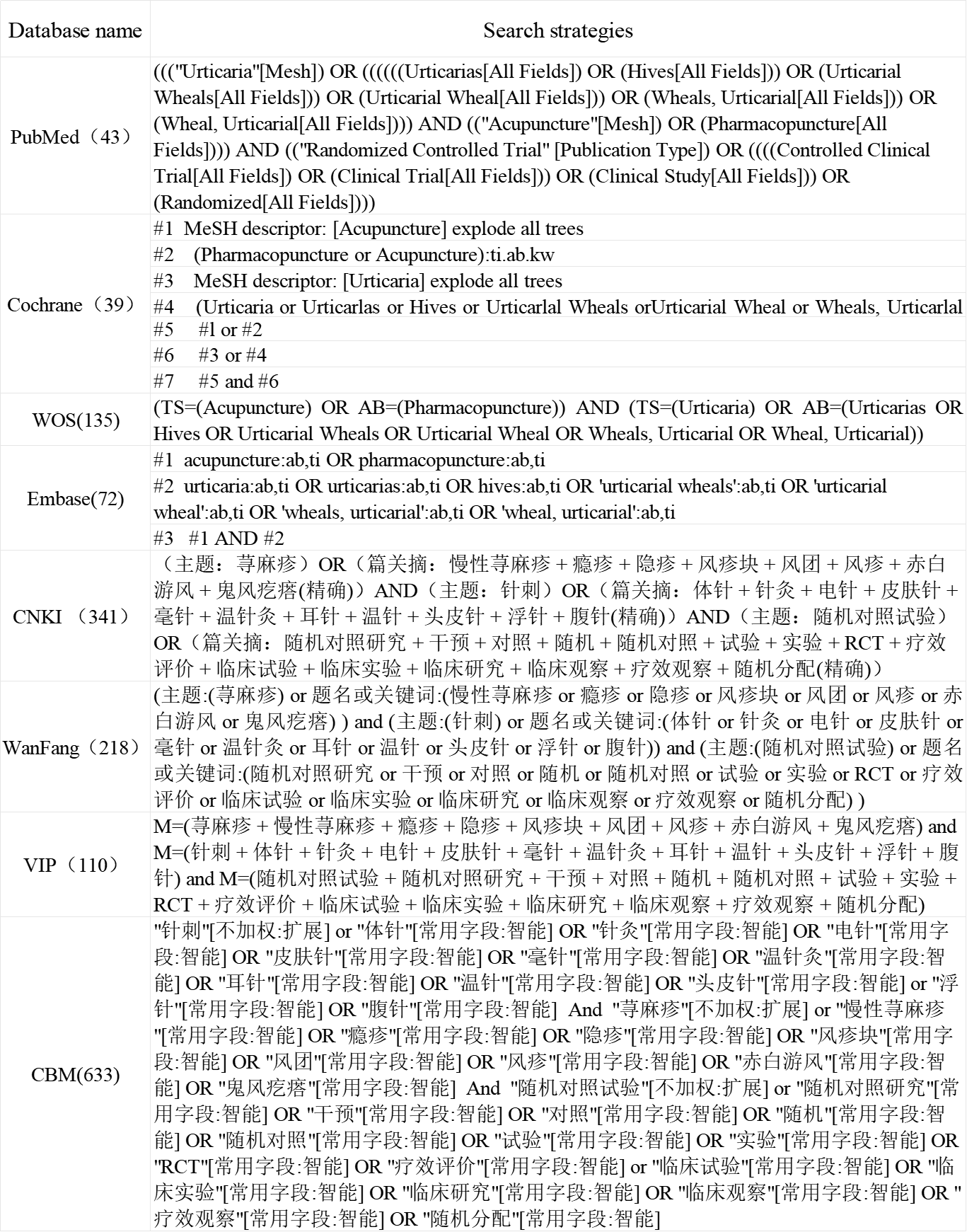

Supplement: Supplementary file 1 [file Supplementary_file_1.docx]
