# Supplementary material for: Clinical efficacy and safety of acupuncture in the treatment for chronic spontaneous urticaria: a systematic review and meta-analysis
Source: Front Med (Lausanne). 2025 May 30;12:1498795. doi: 10.3389/fmed.2025.1498795 (PMC12164643; doi:10.3389/fmed.2025.1498795)
Supplement: Supplementary file 2 [file Supplementary_file_2.docx]

**Identification of studies via databases and registers**

Articles identified through database search(n=1591)

PubMed (n=43）

Embase (n=72)

Web of Science (n=135)

Cochrane CENTRAL (n=39)

Wan Fang (n =218 )

VIP(n =110)

CNKI (n =341)

CMB(n =633)

**Identification**

**Screening**

Excluded due to duplicate (n=449)

Articles screened by title and abstract (n=1142)

Records excluded after reading titles and abstracts.(n=1067)

Research Mechanisms(n=13)

Summarize (n=56)

Animal Experimentation(n=20)

Experience(n=30)

Conference Papers(n=4)

Non-RCTs(n=357)

Irrelevant RCTs(n=587)

Non-RCTs(n=11)

Incomplete Text（n=8）

Data duplication(n=1)

Intervention incompatibility(n=33)

**Included**

Final inclusion of literature(n =22)

Number of documents read in full(n=75）
