# Supplementary material for: Clinical efficacy and safety of acupuncture in the treatment for chronic spontaneous urticaria: a systematic review and meta-analysis
Source: Front Med (Lausanne). 2025 May 30;12:1498795. doi: 10.3389/fmed.2025.1498795 (PMC12164643; doi:10.3389/fmed.2025.1498795)
Supplement: Supplementary file 3 [file Supplementary_file_3.docx]

1.Efficacy Rate


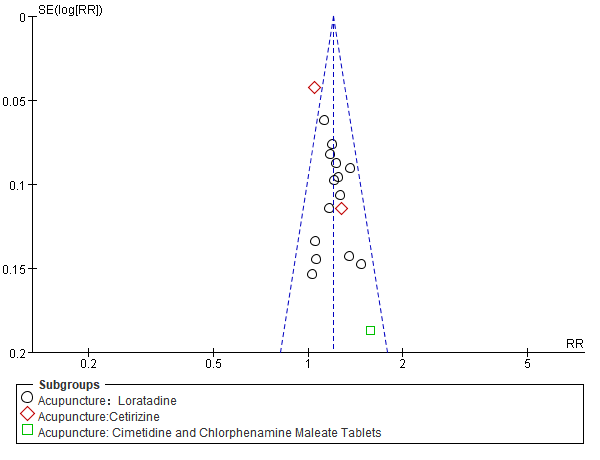


Funnel plots of subgroup analyses of effectiveness of different intervention methods


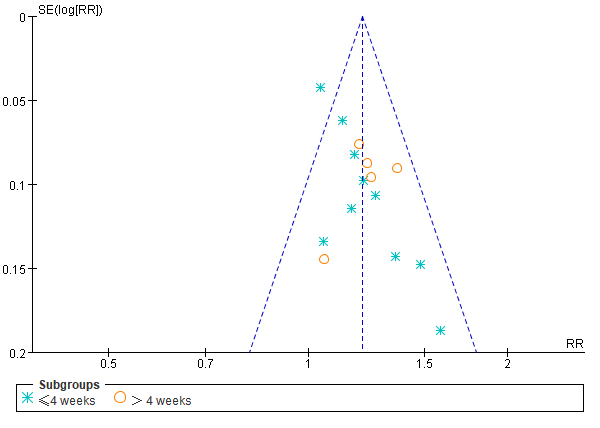


Funnel plots of subgroup analyses of effectiveness across treatment cycles.


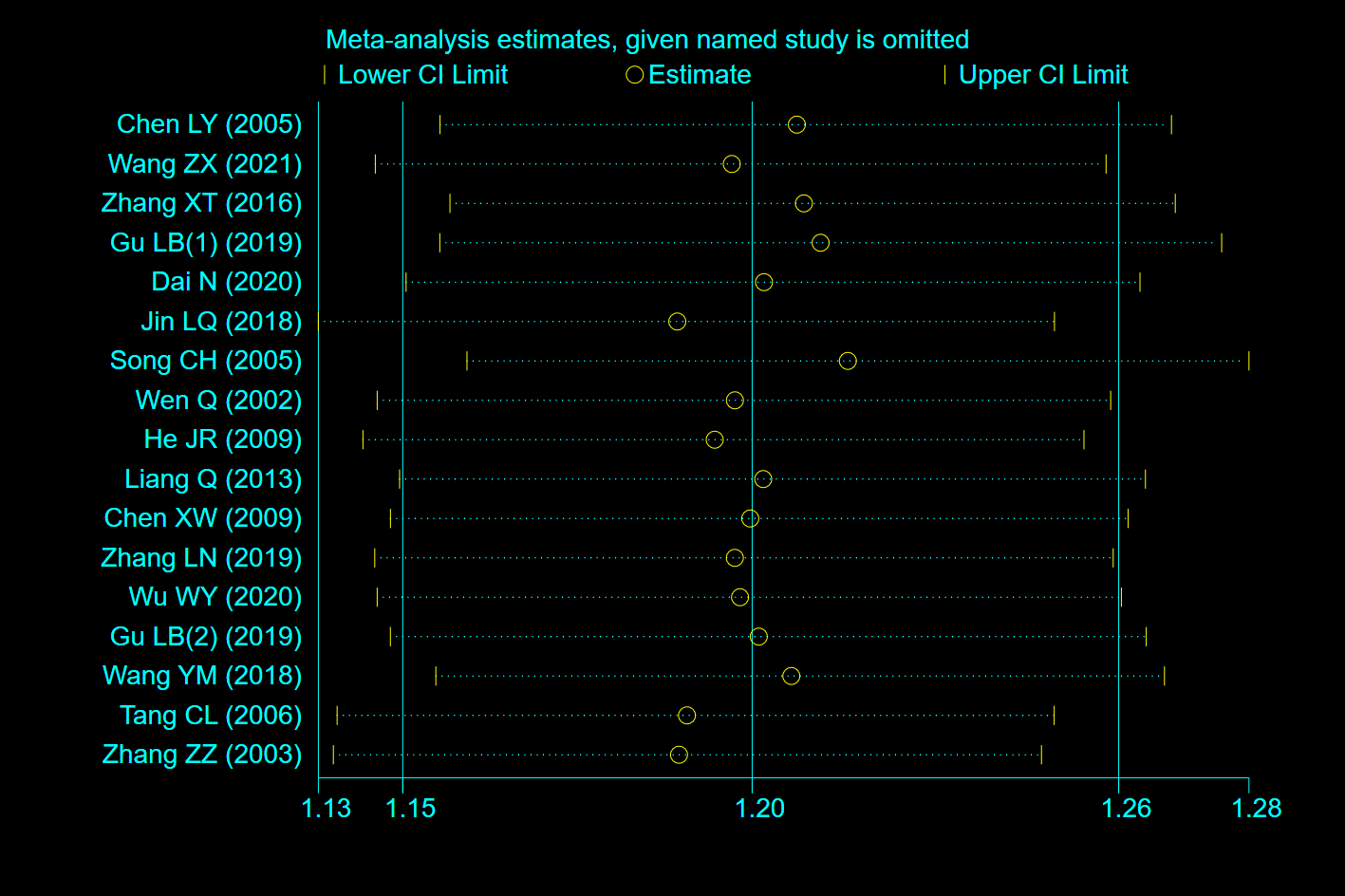


Sensitivity analysis


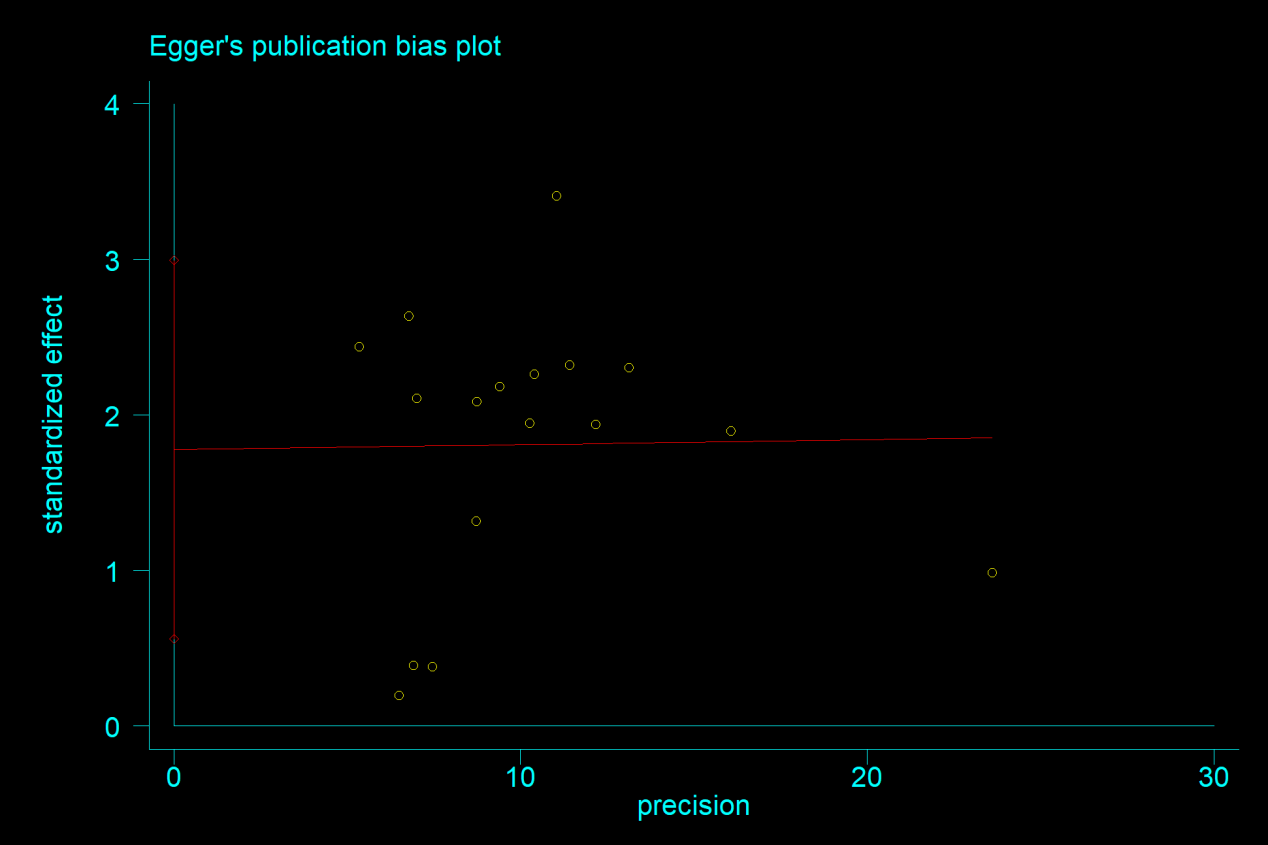


Egger's test1.


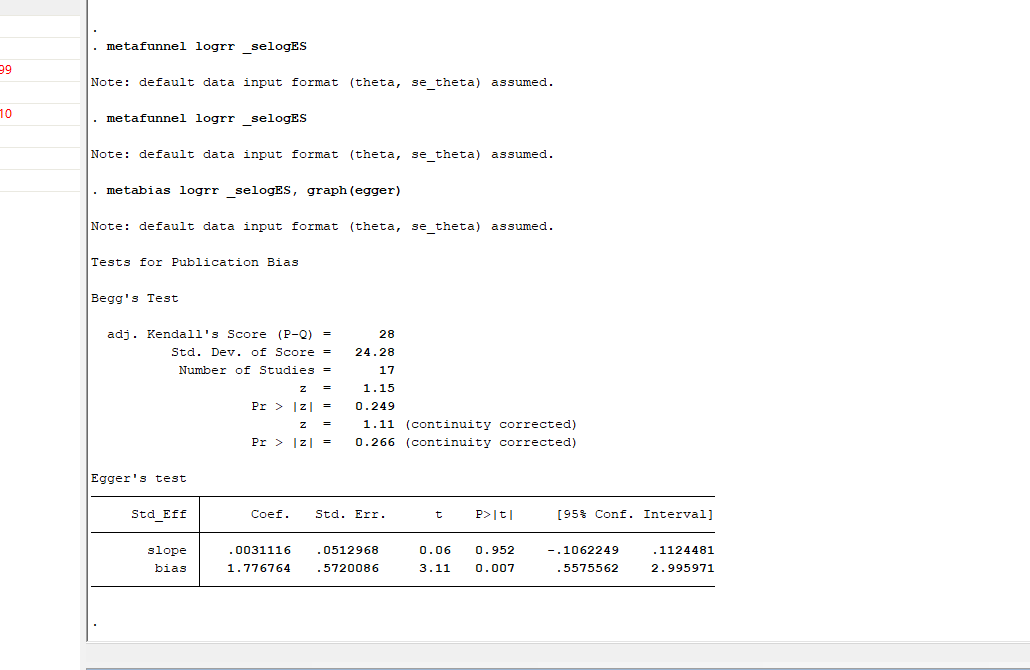
Egger's test1.

2.


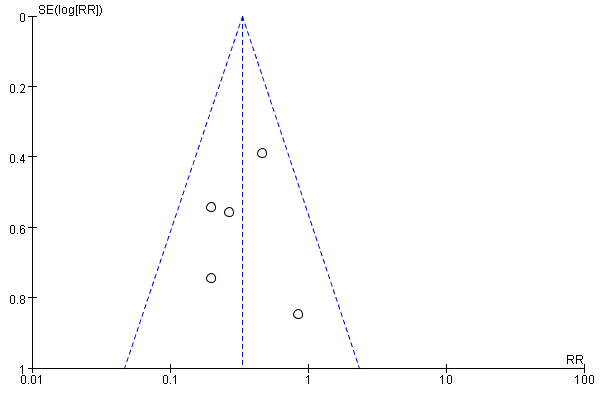


Funnel plot


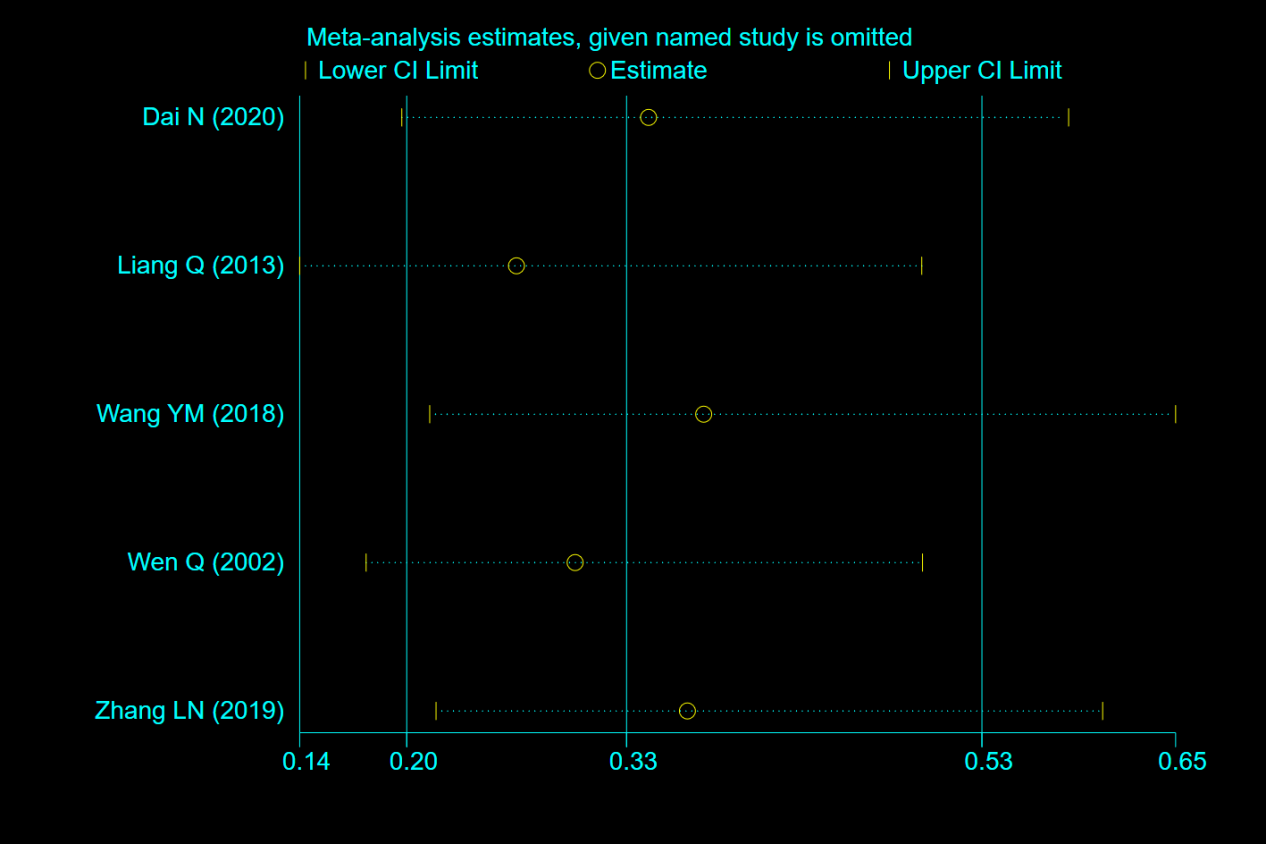


Sensitivity analysis


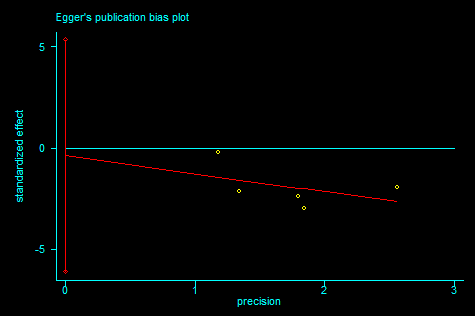


Egger's test1


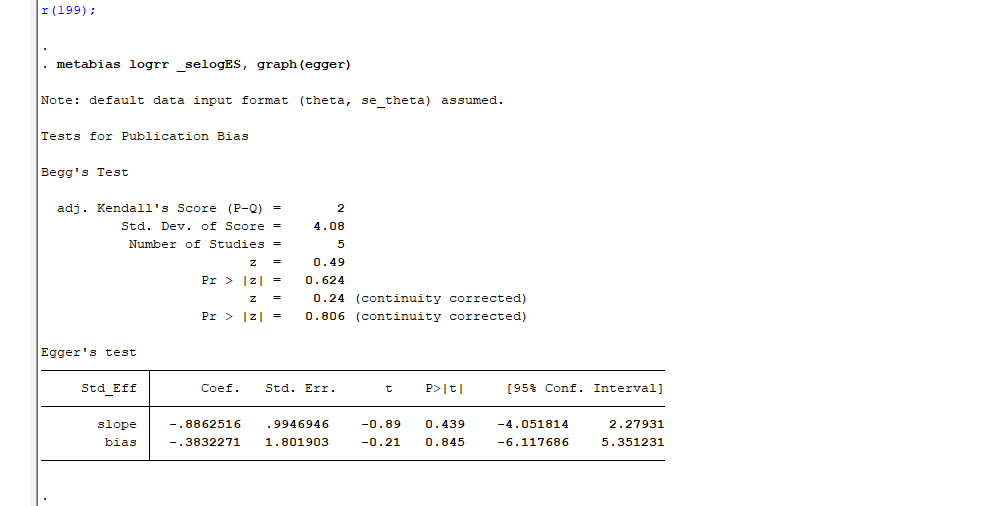


Egger's test 2

1. UAS7


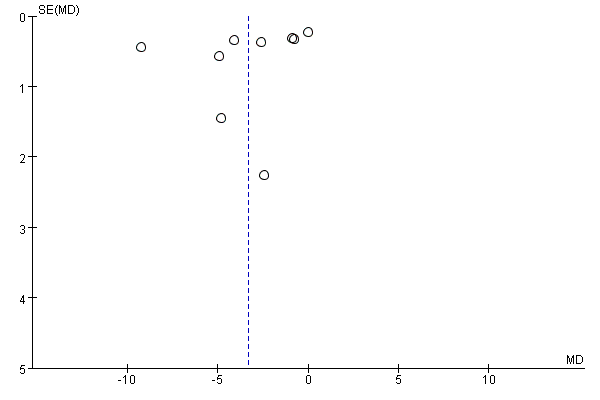
Funnel plot1


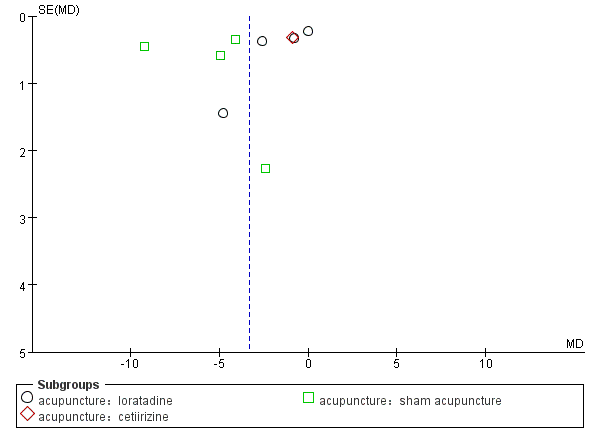


Funnel plot 2


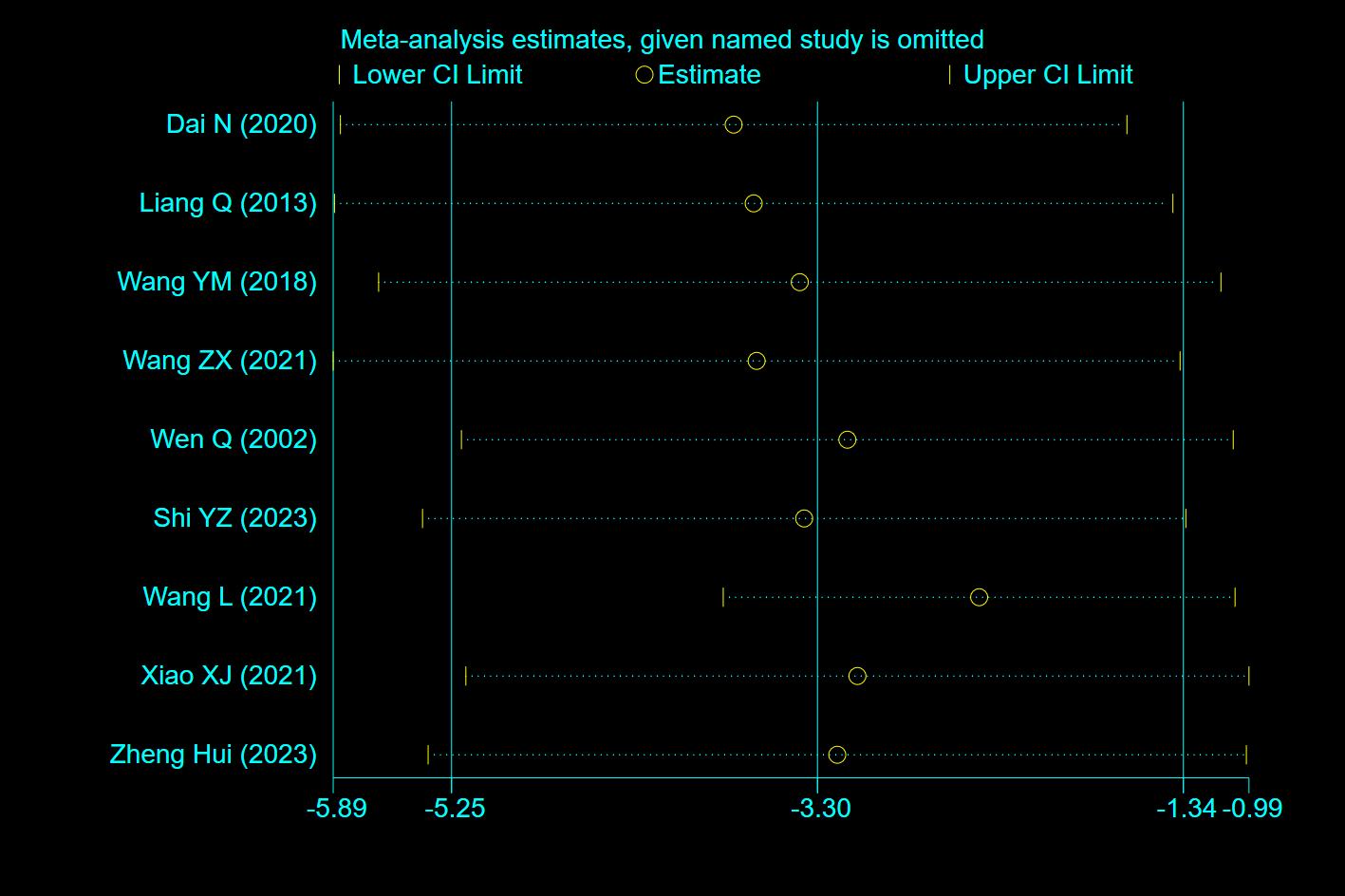
Sensitivity analysis


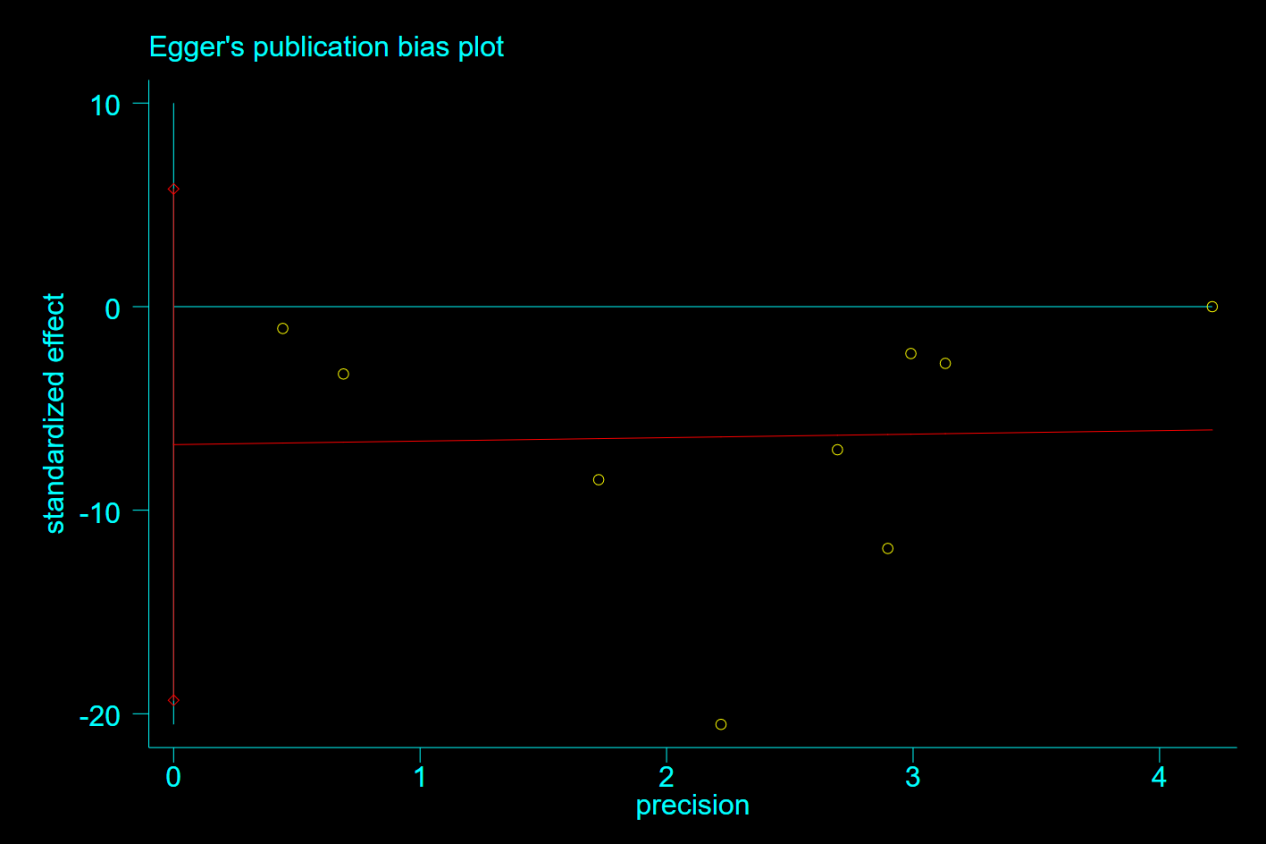


Egger's test1


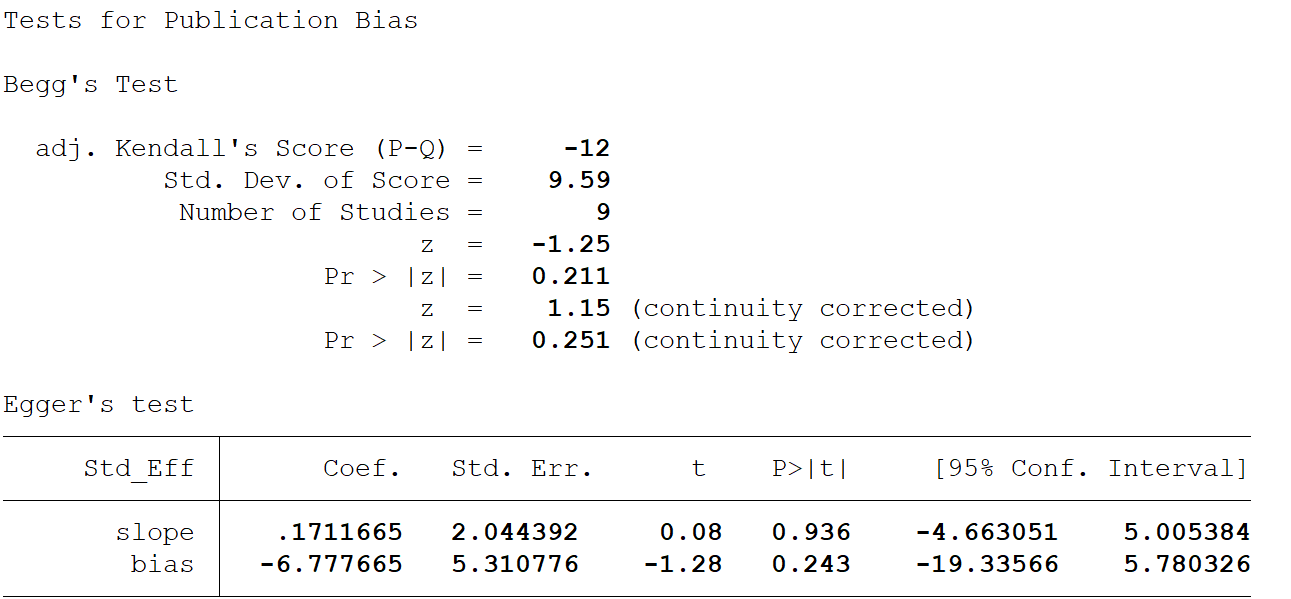


Egger's test2

1. DLOI


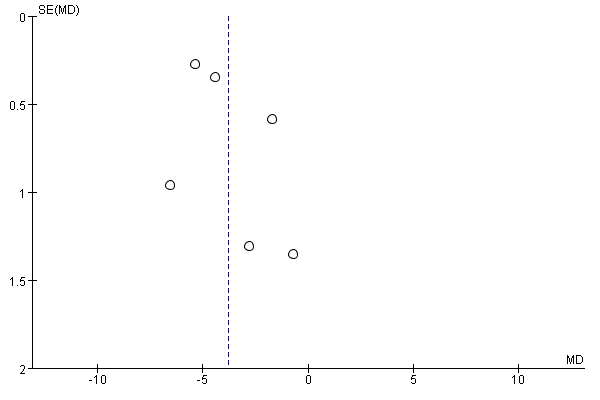


Funnel plot1


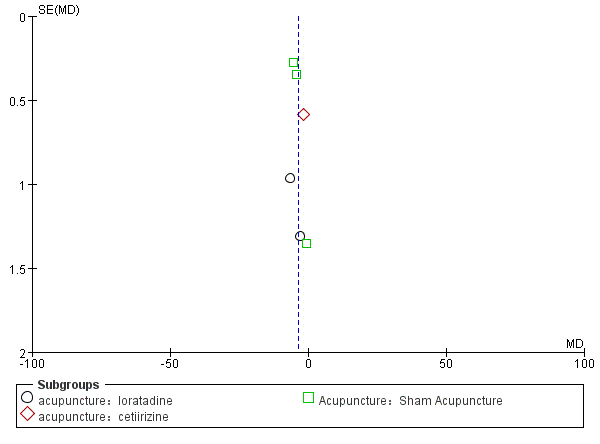


Funnel plot 2


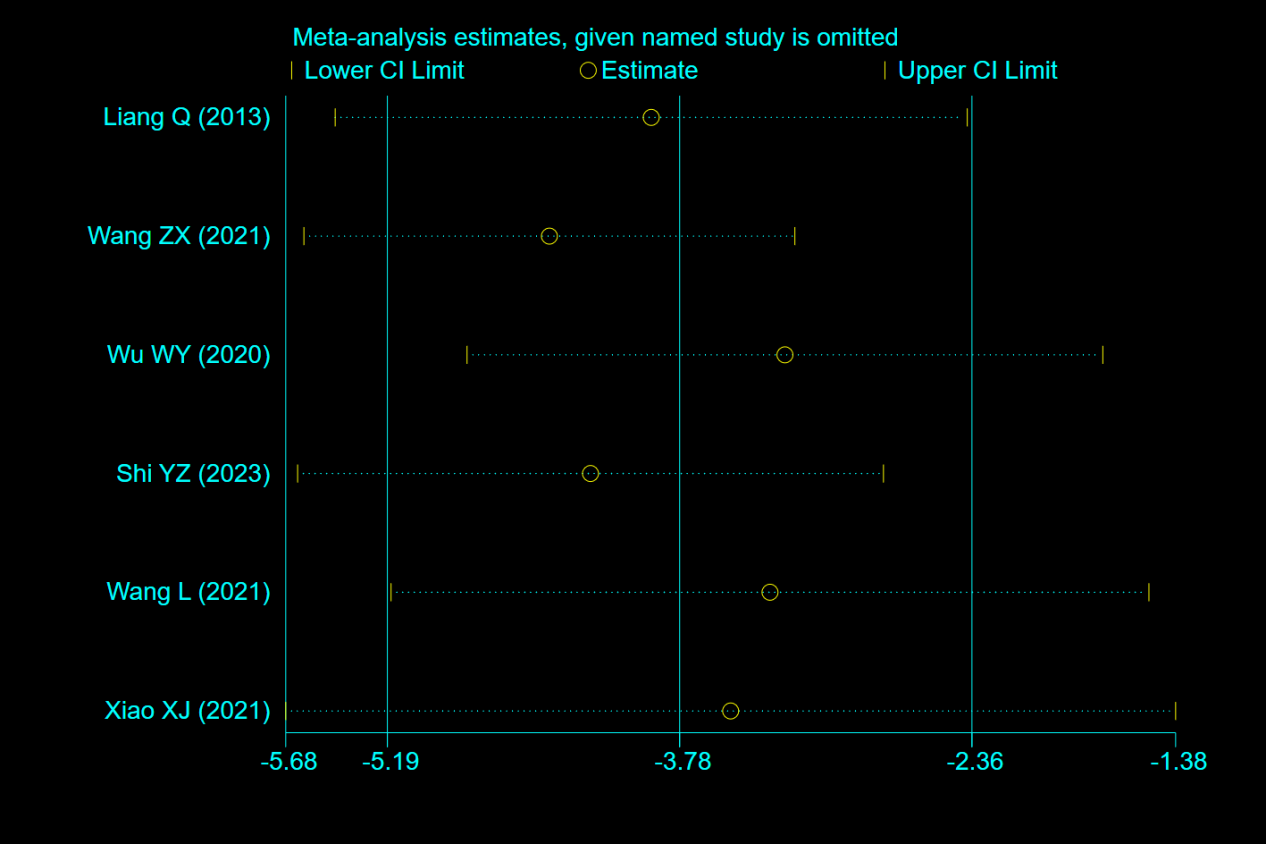


Sensitivity analysis


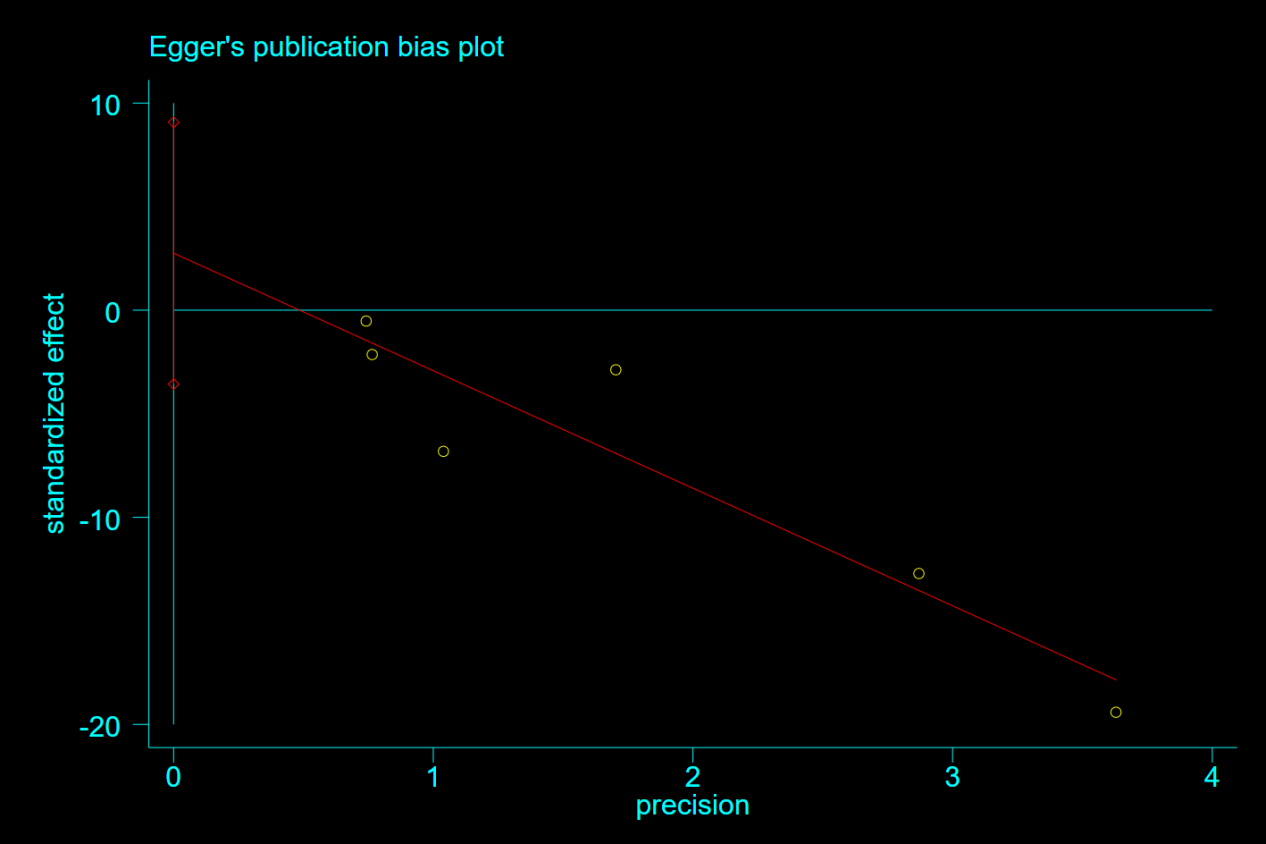


Egger's test1


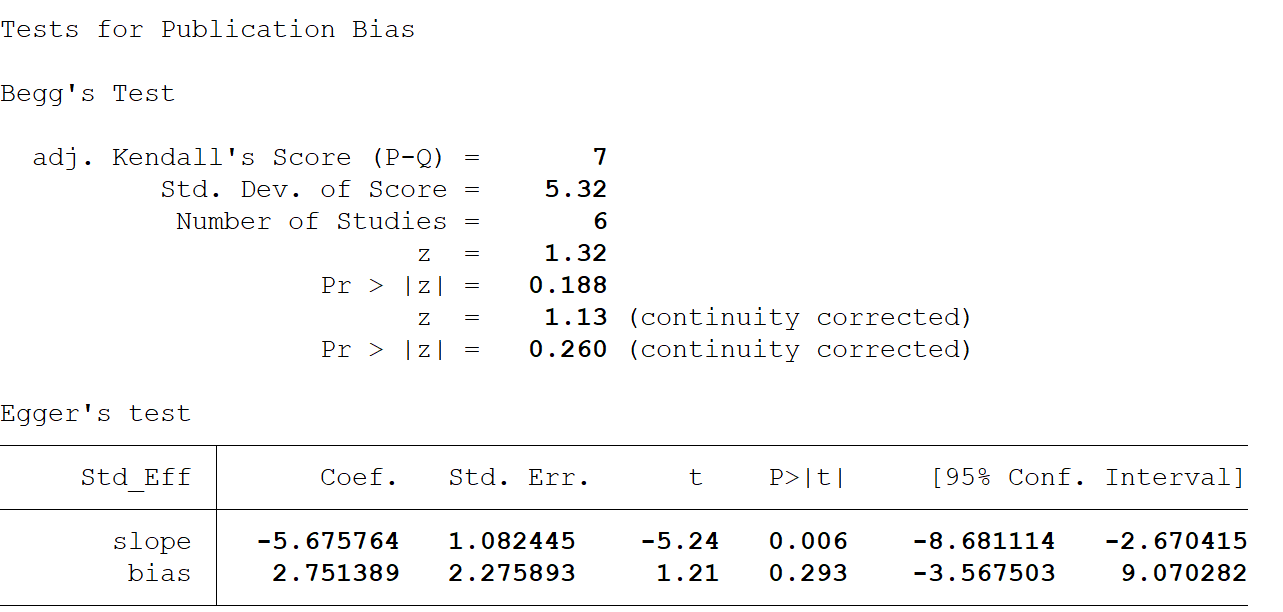


Egger's test 2

1. HADM


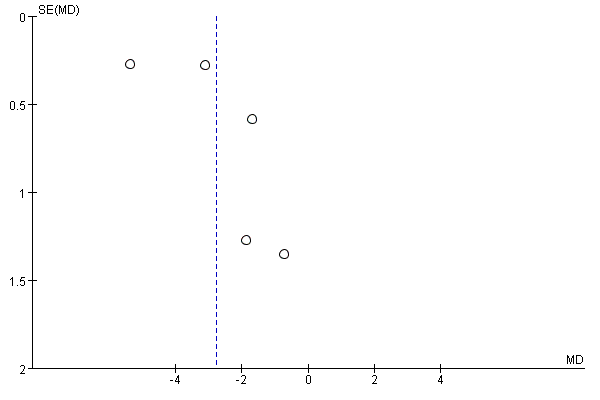


Funnel plot1


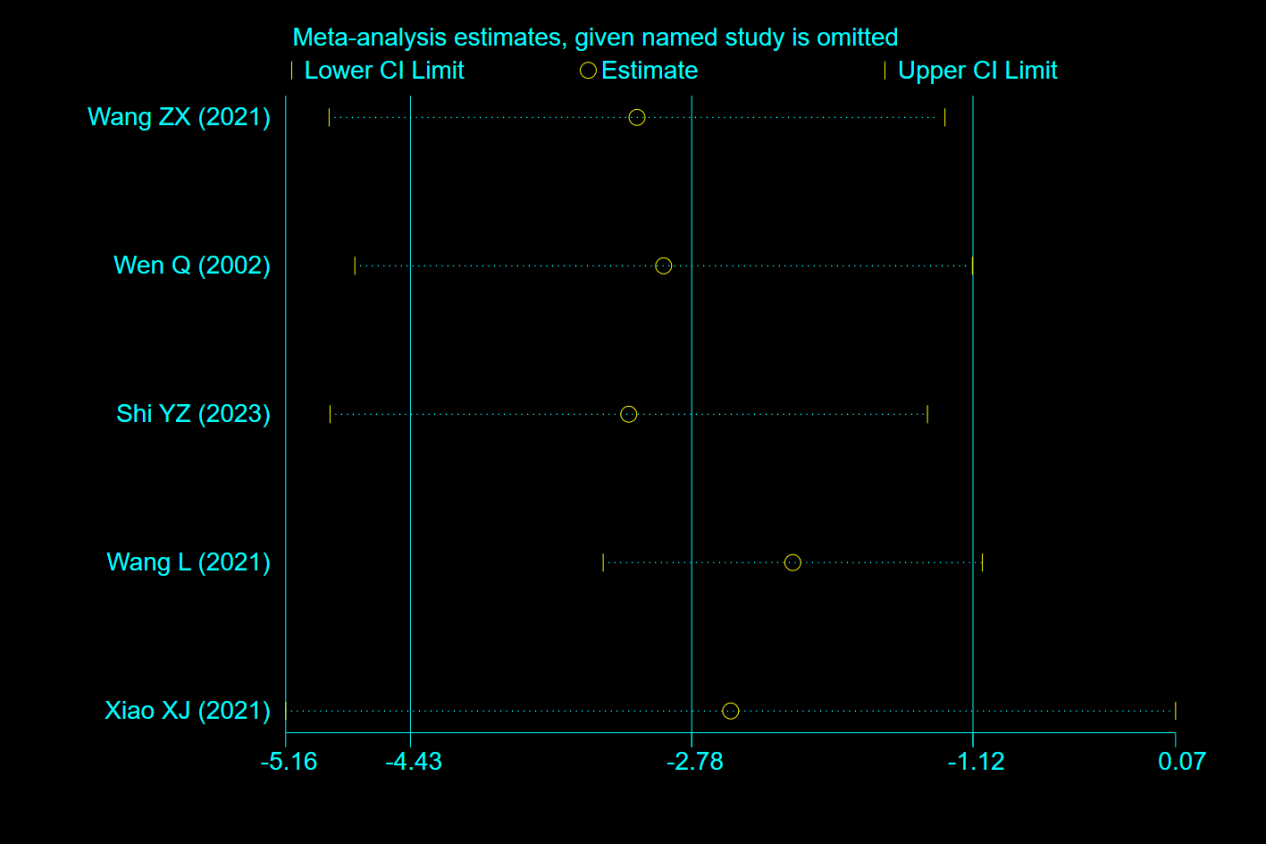


Sensitivity analysis


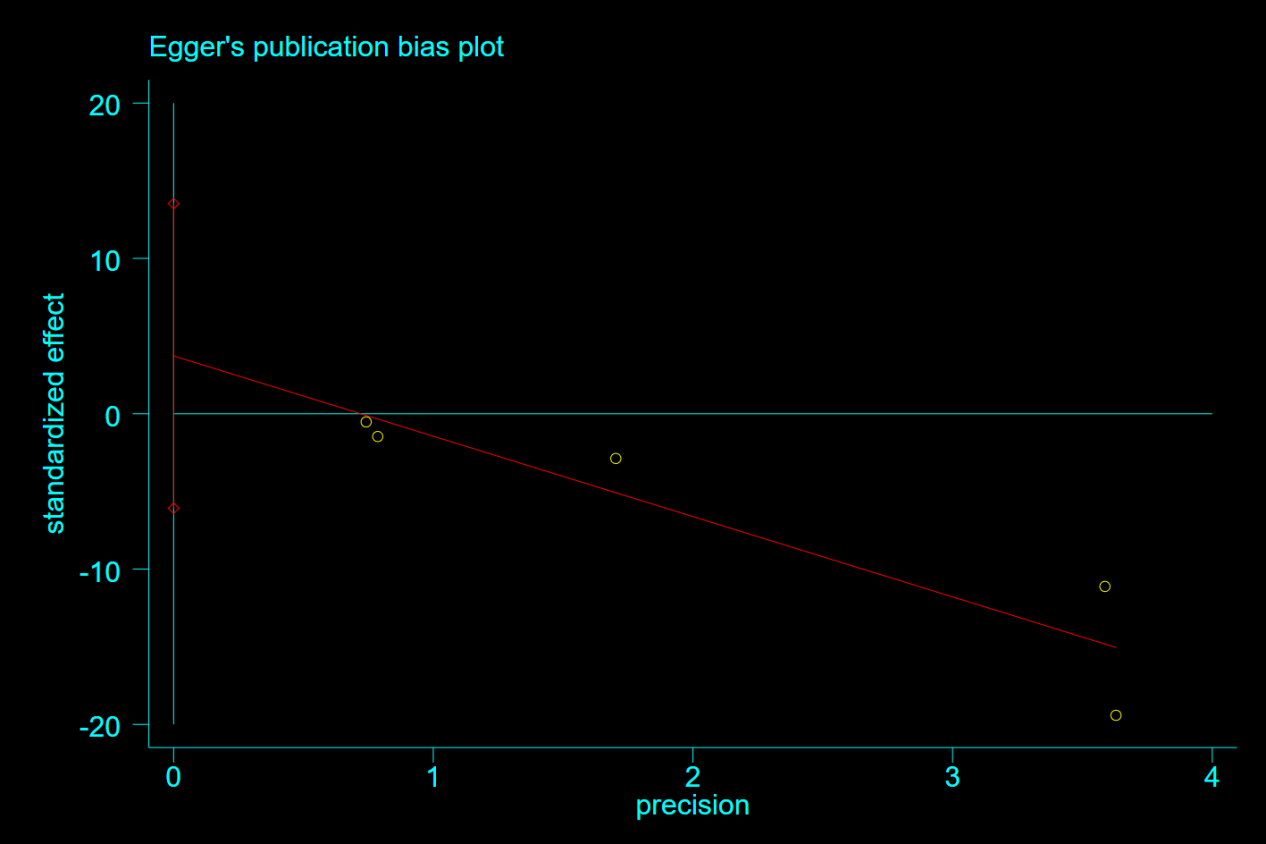


Egger’S test1


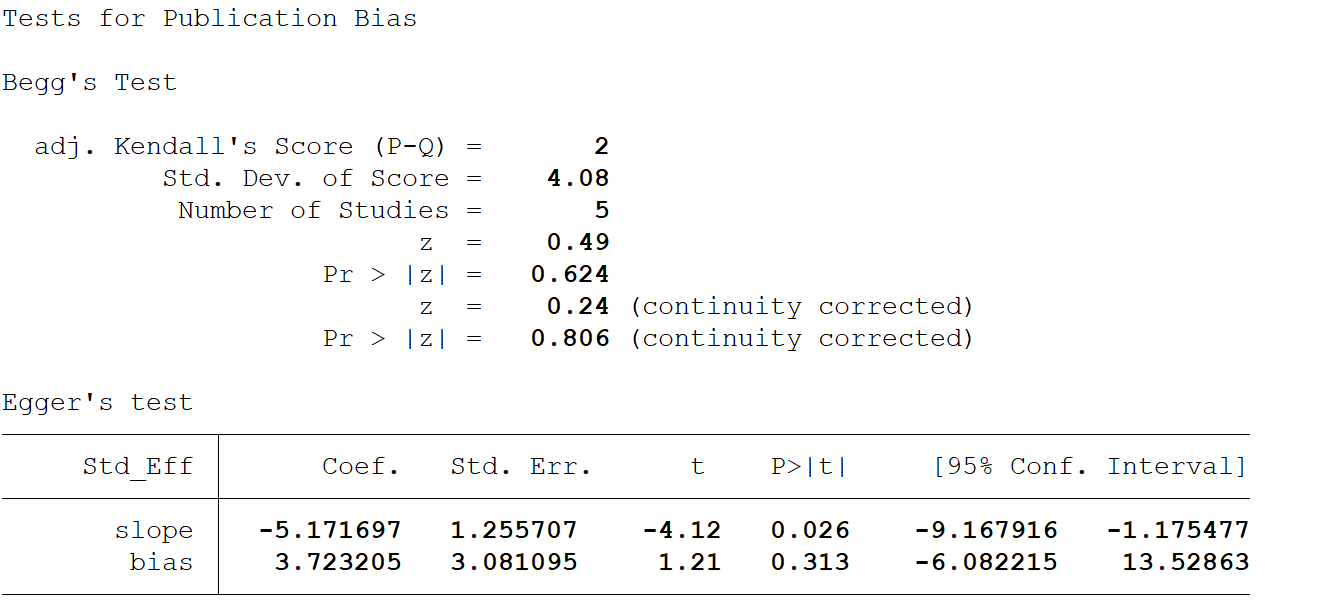


Egger’S test 2

1. Number of Urticaria Wheals


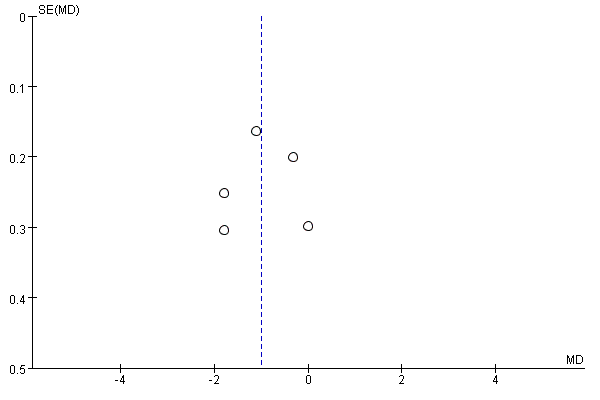


Funnel plot


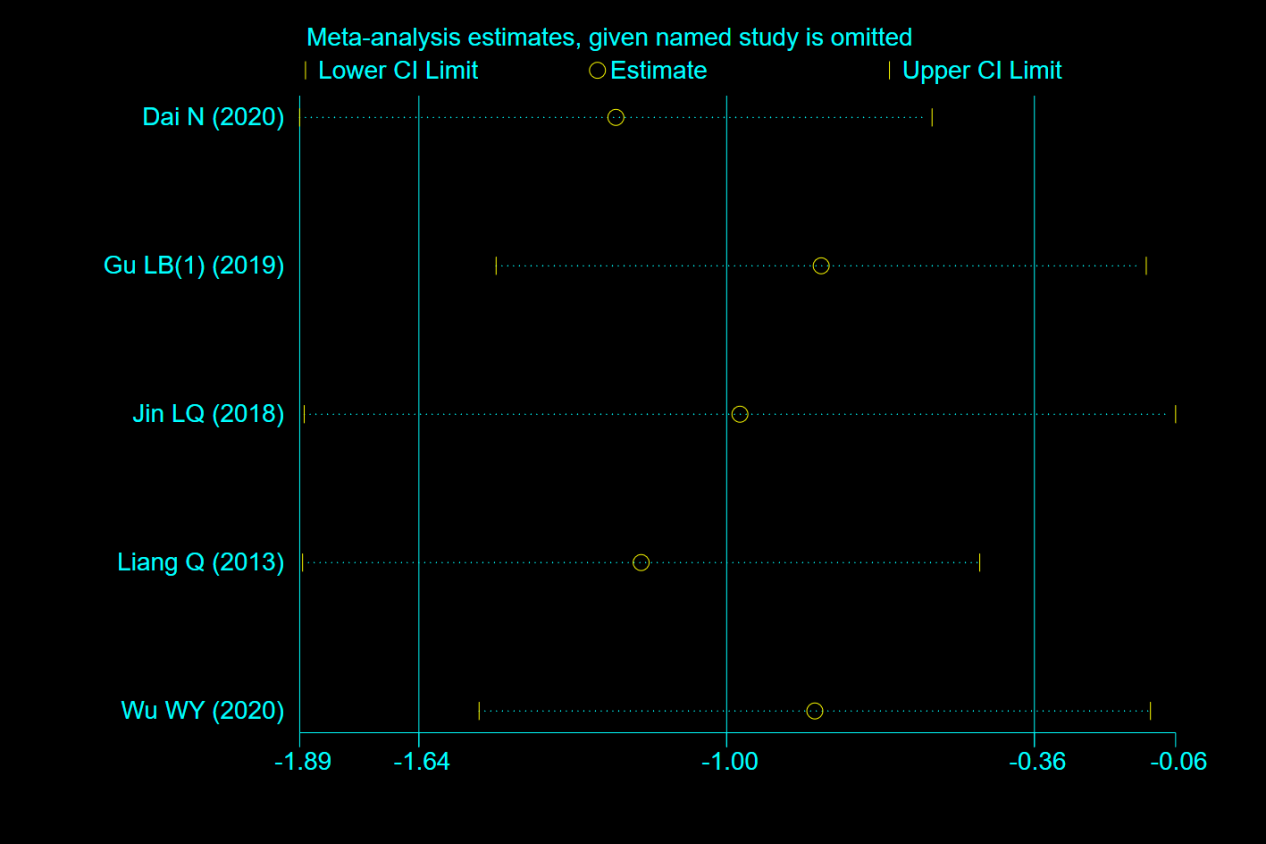


Sensitivity analysis


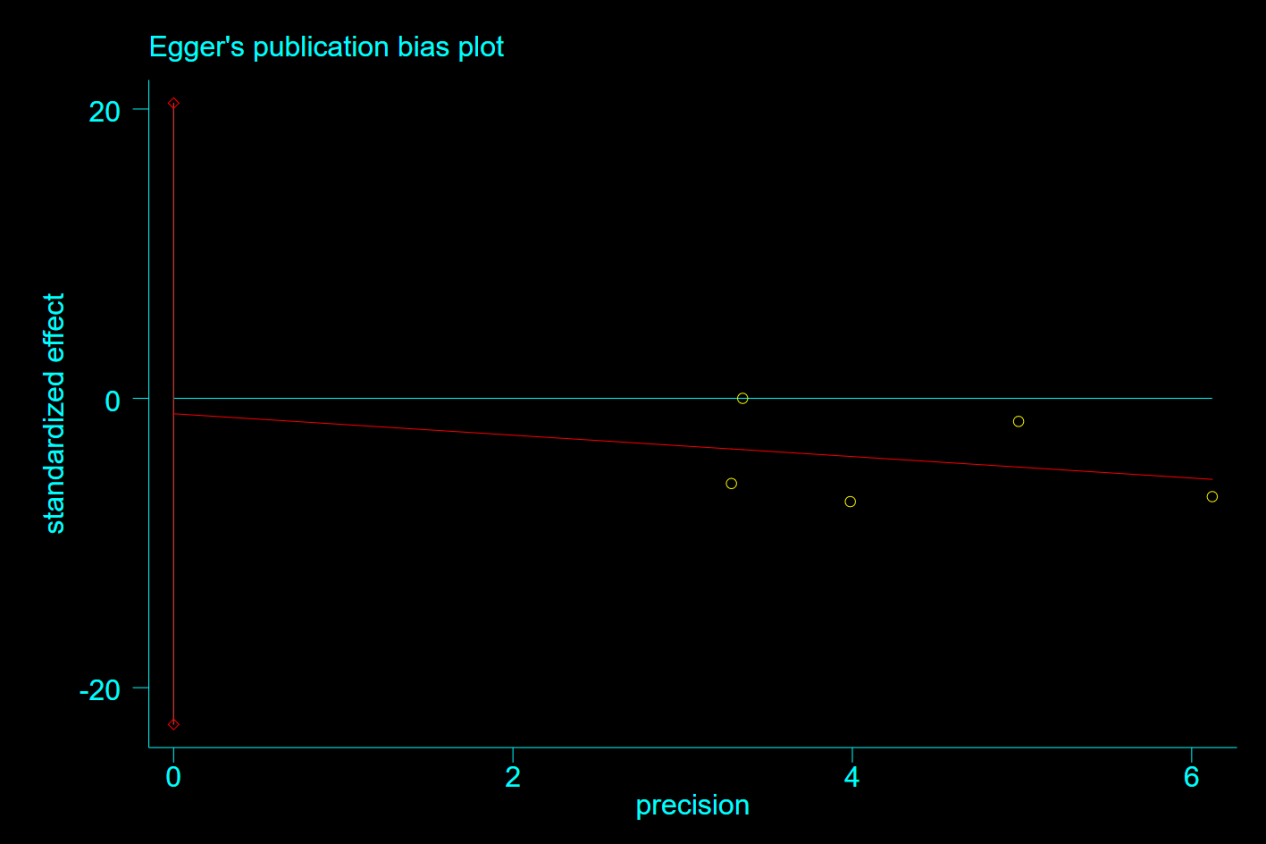
Egger's test1


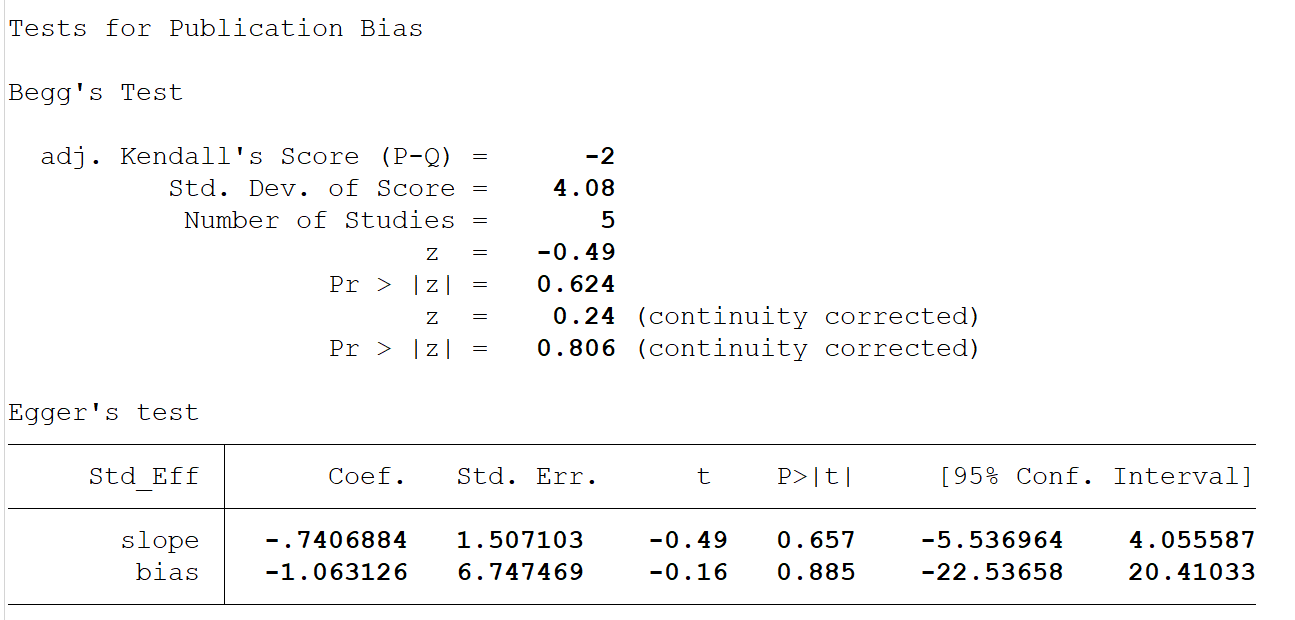


Egger's test 2

1. Itch Severity


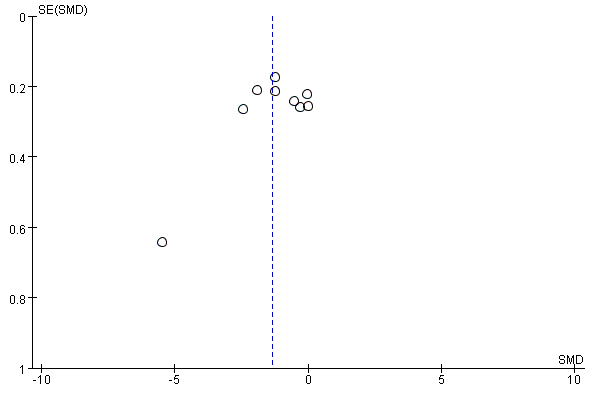


Funnel plot1


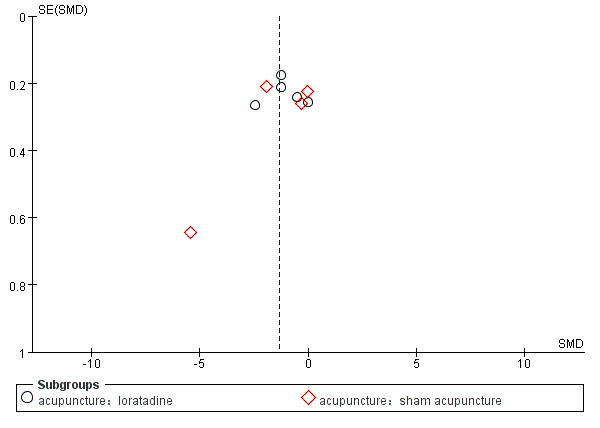


Funnel plot 2


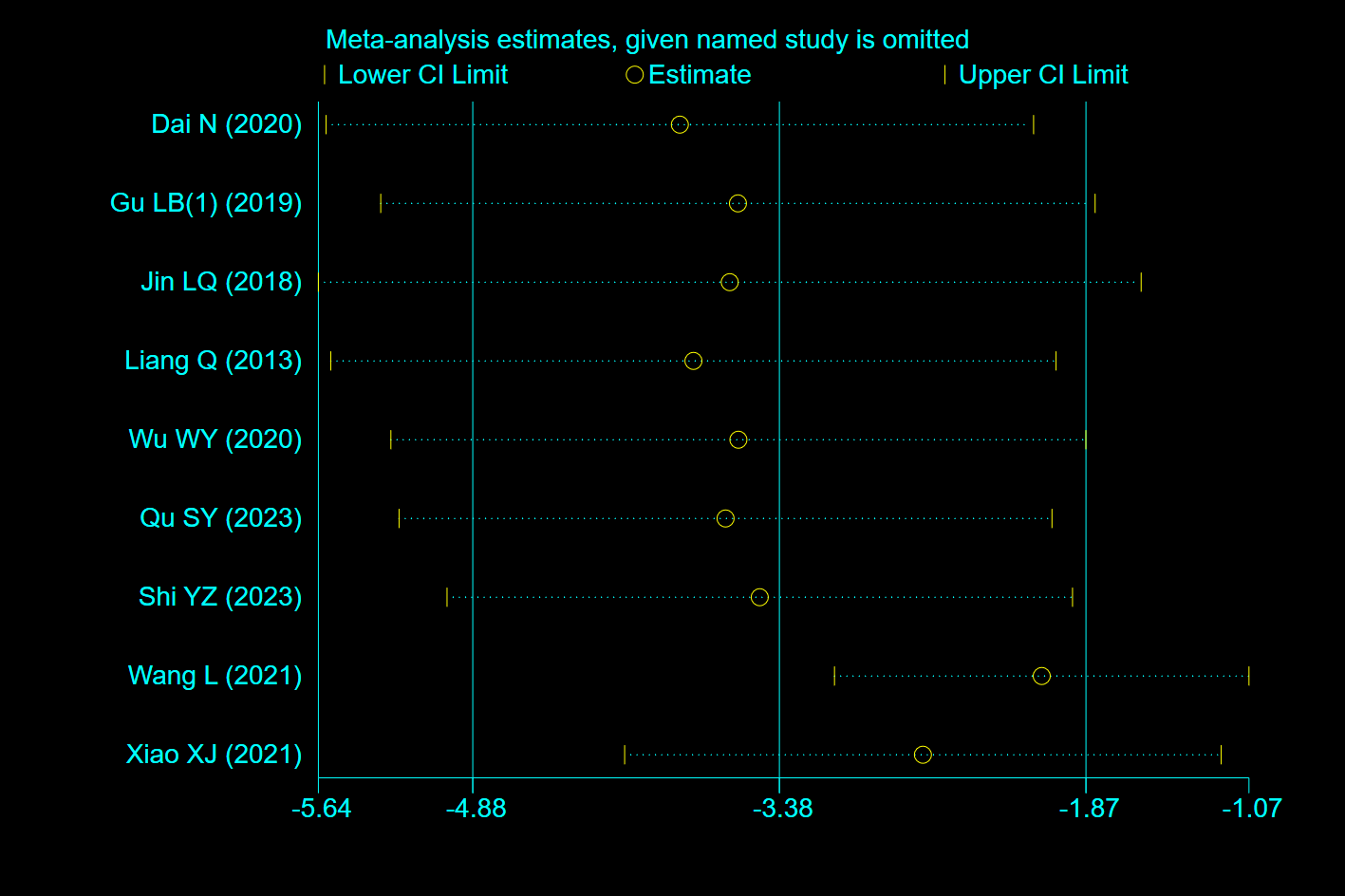


Sensitivity analysis


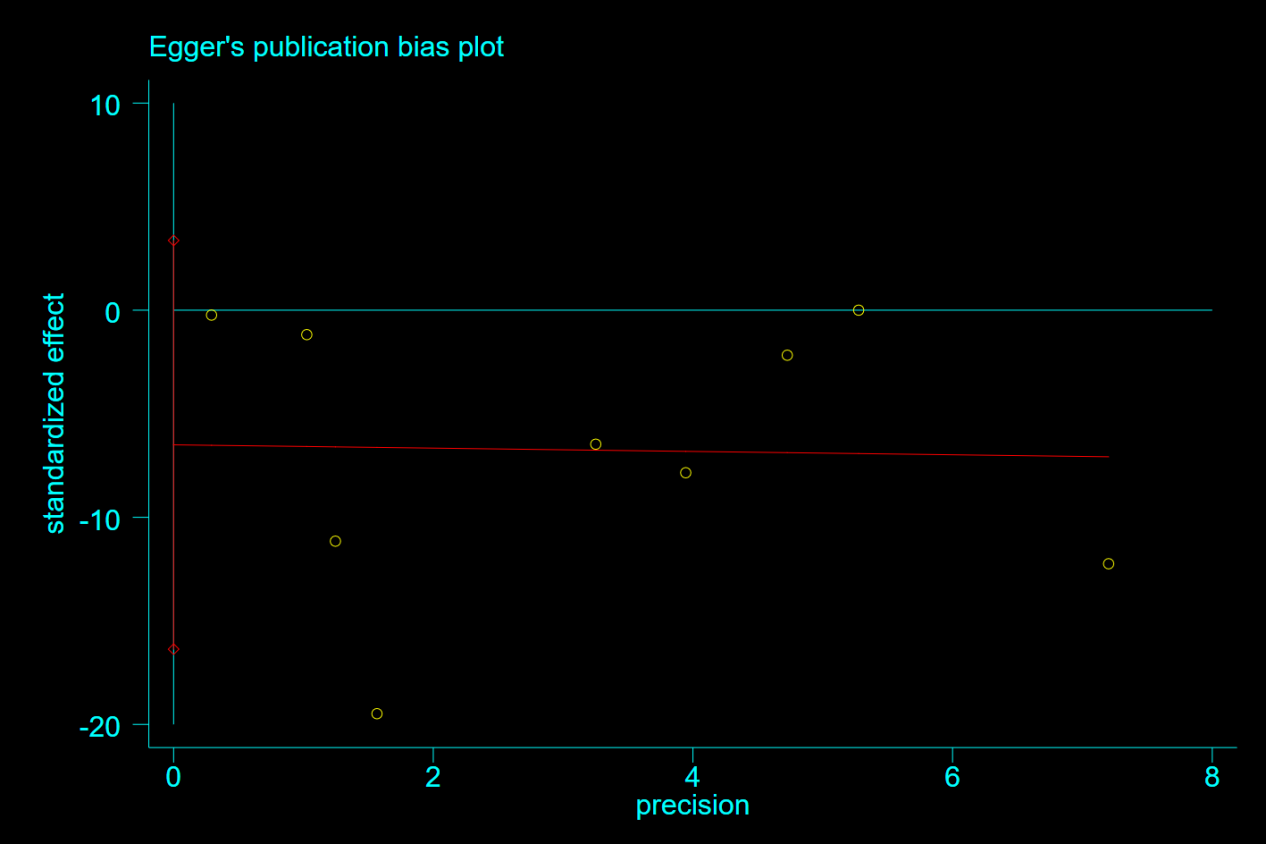
Egger's test1


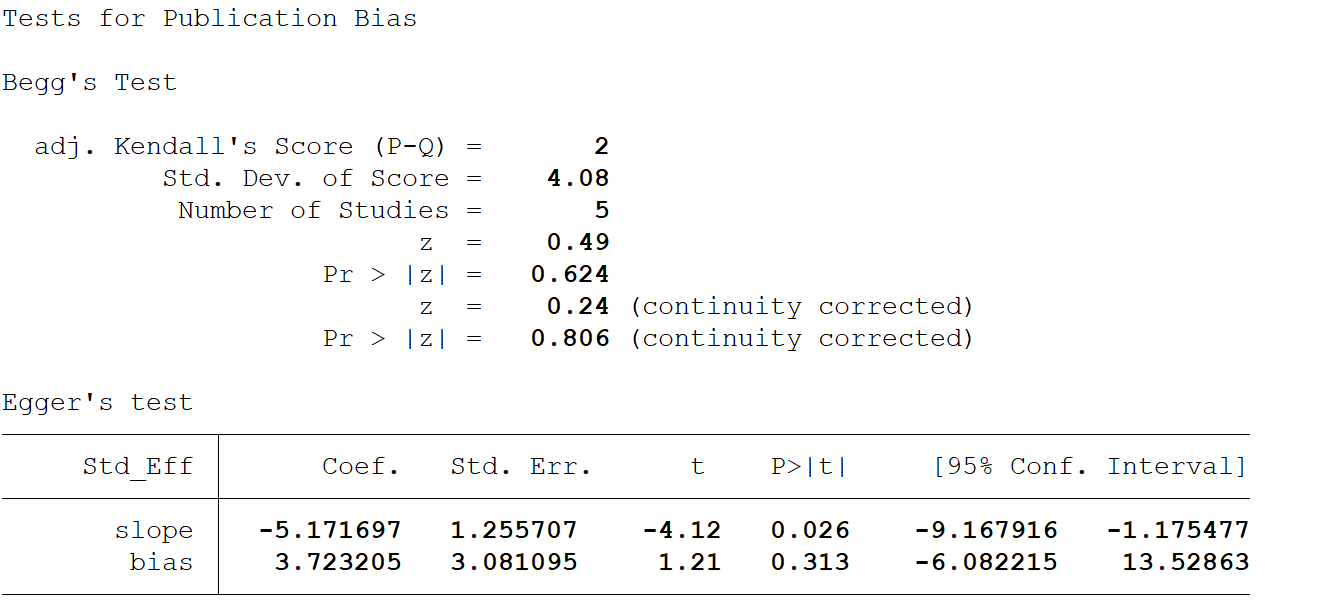


Egger's test 2

1. Safety Indicators


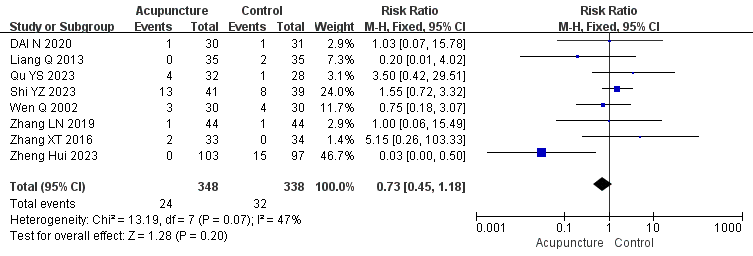


Forest plot1


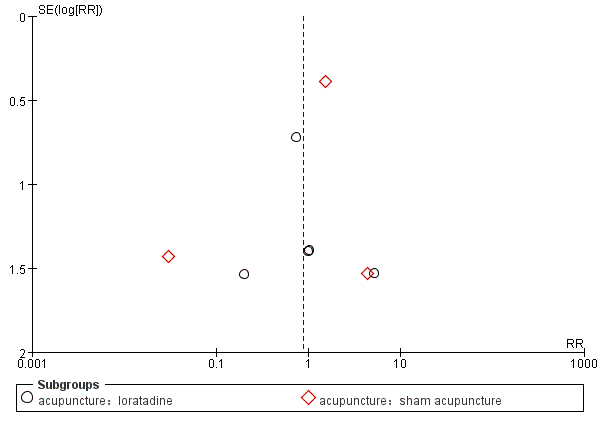


Forest plot 2


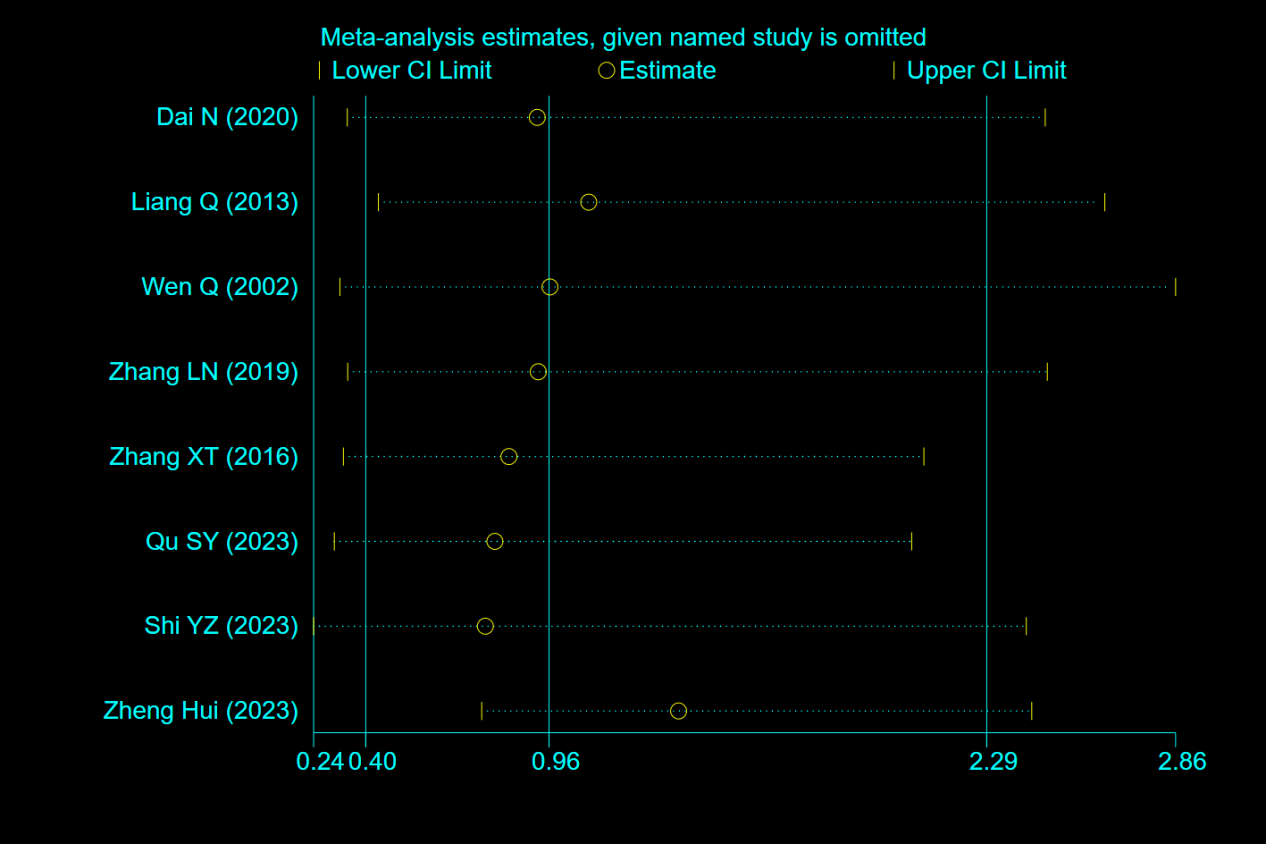


Sensitivity analysis


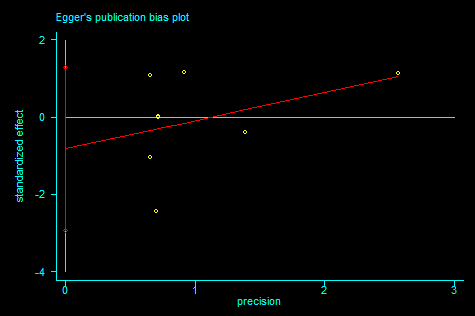


Egger's test1


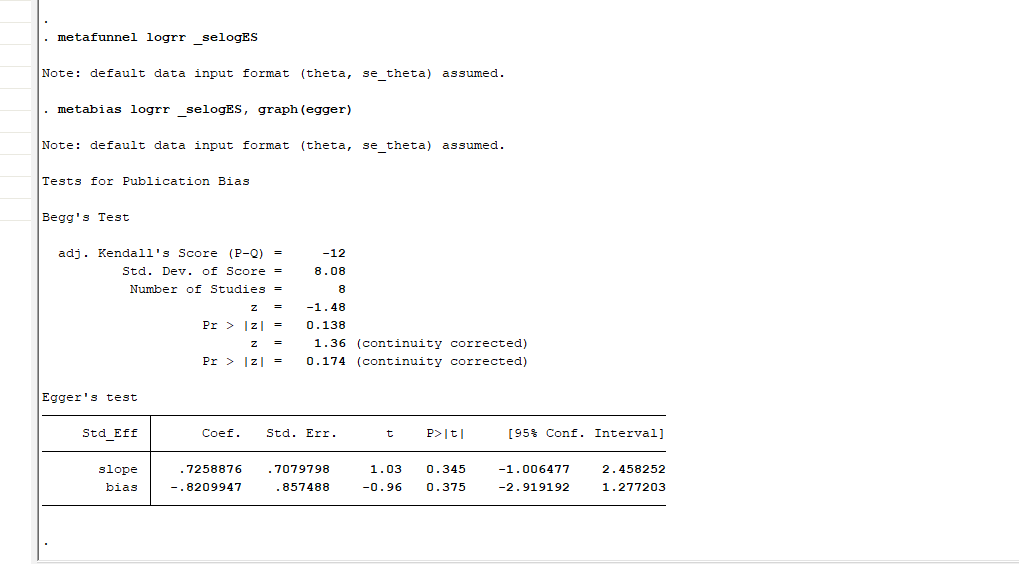


Egger's test 2
