# Supplementary material for: Clinical efficacy and safety of acupuncture in the treatment for chronic spontaneous urticaria: a systematic review and meta-analysis
Source: Front Med (Lausanne). 2025 May 30;12:1498795. doi: 10.3389/fmed.2025.1498795 (PMC12164643; doi:10.3389/fmed.2025.1498795)
Supplement: Supplementary file 4 [file Supplementary_file_4.docx]

**Hamilton Depression Scale (HAMD) Score**

A total of five studies [9, 15, 26, 27, 29] involving 388 patients reported HAMD scores in patients with CSU. Heterogeneity testing revealed significant heterogeneity (chi² = 58.22, p < 0.001, I² = 93%). Therefore, a random-effects model was used for the meta-analysis. The combined results showed a statistically significant improvement in HAMD scoresfor patients treated with acupuncture compared to loratadine, cetirizine, and sham acupuncture [MD = -2.78, 95% CI (-4.43, -1.12), P = 0.001], indicating that acupuncture more effectively reduces depression symptoms in CSU patients (Fig.13). A funnel plot assessment for publication bias showed symmetry on both sides. Further analysis using Egger’s test indicated no significant publicationbias (t = 1.12, p = 0.293). (Refer to Supplementary Material 3 for DLQI Funnel plot 1 and Egger’s test 1.2).
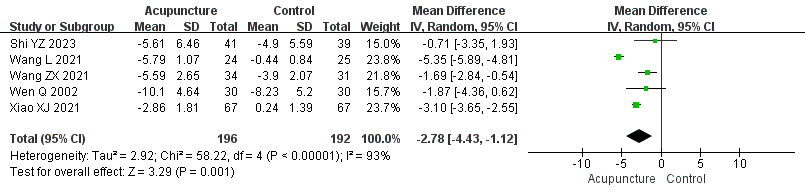
 Fig.1. Forest plot of HAMD.

In the subgroup analysis based on different control interventions (acupuncture vs loratadine, acupuncture vs cetirizine, and acupuncture vs sham acupuncture): Acupuncture vs Loratadine: Only one study [15] was included in this subgroup. The qualitative analysis showed [MD = -1.87, 95% CI (-4.36, -0.62), P = 0.14], indicating no statistically significant difference. Acupuncture vs Cetirizine: One study [9] was included in this subgroup. The qualitative analysis showed [MD = -1.69, 95% CI (-2.84, -0.54), P = 0.004], indicating a statistically significant difference favoring acupuncture. Acupuncture vs Sham Acupuncture: Three studies [26, 27, 29] were included in this subgroup, showing significant heterogeneity (chi² = 39.65, p < 0.0001, I² = 95%). The combined analysis showed [MD = -3.40, 95% CI (-5.42, -1.37), P = 0.001], indicating a statisticallysignificant difference favoring acupuncture (Fig.14). After the subgroup analysis,high heterogeneity persisted, suggesting that other factors may contribute to thevariability. Further investigation into potential sources of heterogeneity is warranted.


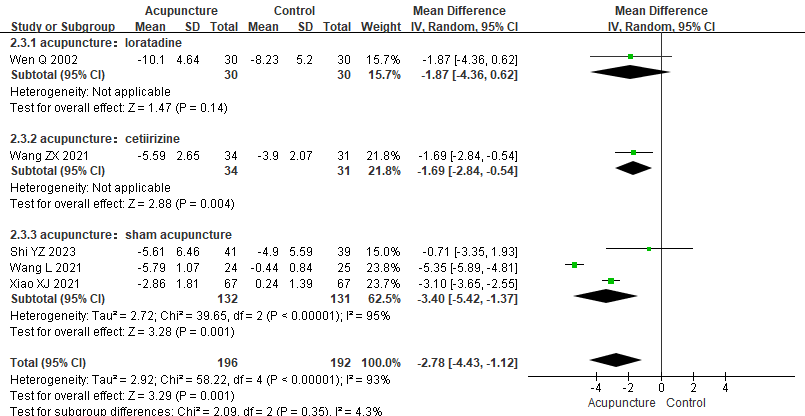


Fig.2. Forest plot of subgroup analyses of HADM of

different intervention methods.

**Size of Urticaria Wheals**

A total of three RCTs [13, 17, 20] involving 276 patients were included in the analysis of wheal size. Heterogeneity testing showed significant heterogeneity (chi² = 11.85, p = 0.003, I² = 83%). A random-effects model was used for the meta-analysis. Due to the different measurement methods—two studies using manual measurement and one using image analysis—SMD was chosen for the meta-analysis. The combined results showed a statistically significant difference favoring acupuncture over

cetirizine in reducing wheal size [SMD = -0.75,95% CI (-1.35, -0.14), P = 0.02] (Fig. 15),indicating that acupuncture is effective in reducing the size of wheals in chronic spontaneous urticaria. Because only three studies were included, a funnel plot and Egger’s test for publication bias were not performed.


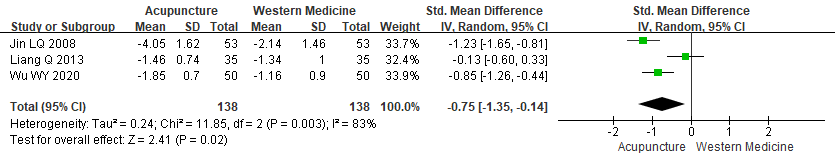


Fig.3. Forest plot of size of urticaria dermatitis.
